# Capsular management strategies in hip arthroscopy for femoroacetabular impingement syndrome: A multilevel meta‐analysis

**DOI:** 10.1002/ksa.70094

**Published:** 2025-10-17

**Authors:** Nikolai Ramadanov, Maximilian Voss, Maximilian Heinz, Robert Hable, Robert Prill, Roland Becker, Ingo J. Banke

**Affiliations:** ^1^ Center of Orthopaedics and Traumatology, Brandenburg Medical School University Hospital Brandenburg an der Havel Brandenburg an der Havel Germany; ^2^ Faculty of Health Science Brandenburg, Brandenburg Medical School Theodor Fontane Brandenburg an der Havel Germany; ^3^ Faculty of Applied Computer Science, Deggendorf Institute of Technology Deggendorf Germany; ^4^ Clinic of Orthopaedics and Sports Orthopaedics, School of Medicine and Health TUM University Hospital, Technical University of Munich Munich Germany

**Keywords:** capsule preservation, capsule repair, femoroacetabular impingement syndrome, hip arthroscopy, capsular management, meta‐analysis

## Abstract

**Purpose:**

To compare three capsular management strategies in hip arthroscopy (capsule preservation [CP], capsule repair [CR] and capsule unrepaired [CU]) for femoroacetabular impingement syndrome (FAIS). We hypothesized that CP and CR would provide superior outcomes compared with CU.

**Methods:**

A systematic search of PubMed, Embase, CENTRAL and Epistemonikos was conducted up to 31 May 2025. Outcomes of CP, CR and CU were compared using a frequentist multilevel random‐effects meta‐analysis with restricted maximum likelihood estimation and Hartung–Knapp adjustment.

**Results:**

Ultimately, 47 primary studies met the inclusion criteria and were included in the meta‐analysis. A total of 7366 hips (7276 patients) were included across the 47 studies. These were distributed into three capsular management groups: (I) CP: 1352 hips, (II) CR: 5043 hips and (III) CU: 971 hips. CR showed the greatest improvement in modified Harris Hip Score with a mean change of 24.00 (95% confidence interval [CI]: 20.86–27.14), while CP achieved the highest MCID rate at 12 months post‐operatively with a mean of 9.30 (95% CI: 7.47–11.14). No other consistent statistically significant differences were observed between groups across post‐operative scores, change scores or complication rates. CP and CR both demonstrated superior outcomes compared to CU in selected functional parameters. All three strategies showed comparable results in pain reduction, revision rate, conversion to total hip arthroplasty and overall complication incidence.

**Conclusion:**

CR and CP yield superior outcomes compared to leaving the CU. Surgeons should close or preserve the capsule, while future trials will clarify the optimal strategy.

**Level of Evidence:**

Level II, systematic review and meta‐analysis of predominantly Level III studies, with additional contributions from Levels I and II studies.

**Abbreviations**
ADL: activities of daily living
BDDH: borderline developmental dysplasia of the hip
BMI: body mass index
CI: confidence interval
CP: capsule preserved
CR: capsule repaired
CU: capsule unrepaired
DAA: direct anterior approach
FAI: femoroacetabular impingement
HAGOS: Copenhagen Hip and Groin Outcome Score
HAS: hip arthroscopy
HOOS: Hip Disability and Osteoarthritis Outcome Score
HOS: Hip Outcome Score
iHOT: International Hip Outcome Tool
MCID: minimal clinically important difference
mHHS: modified Harris Hip Score
NAHS: Non‐Arthritic Hip Score
NRS: Numeric Rating Scale
PRISMA: Preferred Reporting Items for Systematic Reviews and Meta‐Analyses
PROM: patient‐reported outcome measure
PROSPERO: International Prospective Register of Systematic Reviews
RCT: randomized controlled trial
RoB: risk of bias
ROBINS: Risk Of Bias In Non‐randomized Studies of Interventions
SSS: Sports Subscale
THA: total hip arthroplasty
VAS: visual analogue scale

## INTRODUCTION

Hip arthroscopy (HAS) often requires capsular management to access and treat labral, ligamentum teres, acetabular rim, or femoral head–neck pathology. Optimal handling of the capsule is important for joint stability, outcomes, and complication prevention [45]. Current surgical practice varies, with three main strategies: (i) Capsule preserved (CP)—no formal capsulotomy, only periportal access through dilatation, widening, or puncture capsulotomy; (ii) Capsule repaired (CR)—a formal interportal or T‐shaped capsulotomy followed by closure with sutures or plication; and (iii) Capsule unrepaired (CU)—a formal capsulotomy of similar type and size left open [45]. Periportal approaches may preserve stability without formal repair, while full repair can reduce post‐operative microinstability [45]. Because the capsule is highly innervated, extensive procedures may cause pain or sensory disturbance [97]. The role of capsular leakage in fluid extravasation and loss of joint lubrication remains unclear.

Several meta‐analyses have evaluated these strategies in femoroacetabular impingement syndrome (FAIS). Ortiz‐Declet et al. reviewed 34 studies and suggested that closure or plication may improve short‐term outcomes in instability‐prone patients [75]. However, they did not report pooled effect sizes or heterogeneity. Liu et al. found no significant differences between closure and non‐closure in modified Harris Hip Score (mHHS), Hip Outcome Score—Sports Subscale (HOS‐SSS), revisions, or complications, while Hip Outcome Score—Activities Of Daily Living (HOS‐ADL) scores were slightly better in the non‐closure group despite high heterogeneity [59]. Lin et al. included 12 comparative studies (1185 hips; 5 in meta‐analysis) and reported no differences in function, revision risk or instability, arguing against routine repair [58]. Looney et al. pooled 36 studies (5132 hips) and found that repair led to higher post‐operative mHHS, HOS‐ADL and HOS‐SSS and greater score improvements [61]. Cohen et al. included 36 studies (4744 patients) and reported improved mHHS and higher minimal clinically important difference (MCID) achievement with repair, although statistical significance was inconsistent [18]. Dasari et al. analyzed 11 studies (1897 patients) and showed that complete repair improved function, reduced revisions and lowered total hip arthroplasty (THA) conversion, but follow‐up was short [25]. Carbone et al. reported improved outcomes and survivorship with capsular repair compared to non‐repair, although only three studies met the inclusion criteria, and sample sizes were small [12]. Shen et al. confirmed superior functional outcomes with repair in patients without dysplasia or generalized ligamentous laxity, but their analysis was limited to randomized controlled trials (RCTs) and did not include capsule preservation (CP) [90]. Phillips et al. compared routine repair, partial repair and non‐repair but did not include capsule‐preserving techniques [84].

Given these limitations and the absence of direct comparisons across all three strategies, a more rigorous meta‐analysis is needed. The present multilevel analysis includes CP as a distinct strategy alongside CR and CU. This allows all options to be systematically compared and ranked for effectiveness. Clinically, this is relevant because surgeons must decide whether to preserve, repair or leave the capsule open, yet prior reviews only compared repair versus non‐repair. By adding CP and using a multilevel, MCID‐based approach, this study addresses that gap and provides more practical guidance.

The aim of this study was to systematically review and perform a multilevel meta‐analysis of CP, CR and CU in HAS, based on patient‐reported outcomes. We hypothesized that CP and CR would yield superior outcomes compared with CU.

## METHODS

### Reporting standards and protocol registration

This review was registered in PROSPERO on 10 May 2025 (CRD420251050197). It followed PRISMA guidelines [79], and the checklist is provided as Table S1.

### Information sources and search strategy

We searched PubMed, Embase, CENTRAL (Cochrane Library) and Epistemonikos for studies published up to 31 May 2025. Search strings combined capsule terms (e.g., capsule, capsulotomy, repair and closure) with HAS terms. Strategies were tailored for each database. No limits were applied for language or publication year.

### Eligibility criteria

We included RCTs, observational studies and case series on specific capsular techniques. Case reports, reviews and editorials were excluded. Eligible approaches were: (i) Periportal capsulotomy or puncture/dilatation, typically creating sub‐centimetre openings; (ii) Interportal or T‐shaped capsulotomy (2–6 cm) with repair, usually by side‐to‐side sutures or plication; (iii) Interportal or T‐shaped capsulotomy without repair. Studies were excluded if they: combined groups without separate reporting, lacked clear descriptions, omitted hip function or pain outcomes, had overlapping cohorts (most complete data set chosen), or focused on dysplasia treated with capsular plication.

### Study selection process

Two reviewers screened titles/abstracts and then full texts (M.V. and M.H.). Discrepancies were resolved with a third reviewer (N.R.). Agreement was measured with kappa (*κ*).

### Data extraction

Two reviewers extracted study data independently (M.V. and M.H.). Variables included author, year, country, sample size, demographics, design, risk of bias (RoB), follow‐up and outcomes. Disagreements were resolved by consensus.

### Outcome measures

Primary outcomes were patient‐reported outcome measures (PROMs): mHHS, International Hip Outcome Tool (iHOT), HOS, Hip Disability and Osteoarthritis Outcome Score (HOOS), Non‐Arthritic Hip Score (NAHS) and Copenhagen Hip and Groin Outcome Score (HAGOS). Secondary outcomes were hip pain visual analogue scale (VAS) and the Numeric Rating Scale (NRS), complications (e.g., DVT, PE, nerve injury, infection and haematoma), revisions and THA conversions.

### MCID

If multiple PROMs were reported, they were prioritized: HHS > iHOT > HOS > HOOS > NAHS > HAGOS. The latest follow‐up values were used. PROMs were normalized using conservative published MCID thresholds (Table 1) [48, 51, 85, 86]. The same method was applied to pain outcomes, prioritizing VAS over NRS [4, 24].

**Table 1:** Included PROMs with conservative MCID value.

PROM	MCID value	Reference
mHHS	8.20	[85]
iHOT	12.0	[85]
HOS‐ADL	9.00	[51]
HOS‐SSS	14.50	[85]
HOOS pain	9.00	[51]
HOOS symptoms	9.00	[51]
HOOS ADL	6.00	[51]
HOOS sport and recreation	10.00	[51]
HOOS QoL	11.00	[51]
NAHS	10.00	[86]
HAGOS	12.50	[48]
NRS	1.65	[4]
VAS	1.86	[24]

Abbreviations: ADL, activities of daily living; HAGOS, Copenhagen Hip and Groin Outcome Score; HOS, Hip Outcome Score; HOOS, Hip Disability and Osteoarthritis Outcome Score; iHOT, International Hip Outcome Tool; MCID, minimal clinically important difference; mHHS, modified Harris Hip Score; NAHS, Non‐Arthritic Hip Score; NRS, numeric rating scale; PROM, patient‐reported outcome measure; QoL, quality of life; SSS, Sports Subscale; VAS, visual analogue scale.

### Change in outcome measures

Improvement was calculated as the difference between pre‐ and post‐operative scores. This delta quantified change in function and pain.

### Study quality and bias assessment

RoB was assessed independently by two reviewers (M.V. and N.R.). RoB 2 was applied to RCTs, ROBINS‐I to non‐RCTs [92, 93]. Publication bias was assessed by Begg's test or funnel plots.

### Statistical analysis

We performed a frequentist multilevel meta‐analysis with random effects (inverse variance, REML and Hartung–Knapp adjustment). Means with 95% confidence intervals (CIs) were calculated for CP, CR and CU. Subgroup differences were tested to identify significant contrasts. Heterogeneity was assessed with Higgins' *I* ^2^ (<25% low, 25%–75% moderate and >75% high). Results are shown in forest plots. *p* < 0.05 was considered significant. Analyses were conducted in R (meta, metafor packages) by an experienced statistician (R.H.).

## RESULTS

### Systematic literature search

**Figure 1:**
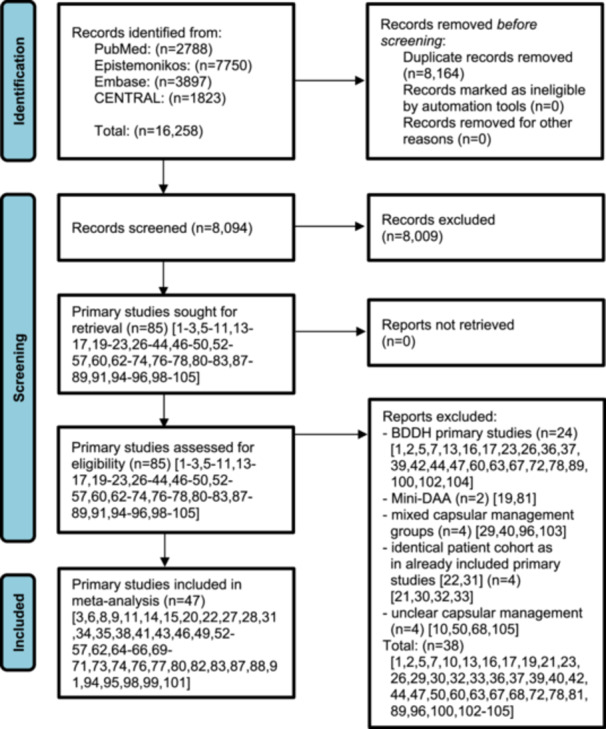
PRISMA chart flow diagram. BDDH, borderline developmental dysplasia of the hip; DAA, direct anterior approach; PRISMA, Preferred Reporting Items for Systematic Reviews and Meta‐Analyses.

The database search yielded 16,258 records. After removing 8164 duplicates, 8094 titles and abstracts were screened. Of these, 8009 were excluded, leaving 85 full‐texts. Thirty‐eight [1, 2, 5, 7, 10, 13, 16, 17, 19, 21, 23, 26, 29, 30, 32, 33, 36, 37, 39, 40, 42, 44, 47, 50, 60, 63, 67, 68, 72, 78, 81, 89, 96, 100, 102, 103, 104, 105] were excluded for the following reasons: 24 focused on BDDH [1, 2, 5, 7, 13, 16, 17, 23, 26, 36, 37, 39, 42, 44, 47, 60, 63, 67, 72, 78, 89, 100, 102, 104], 2 involved mini‐DAA [19, 81], 4 had mixed groups [29, 40, 96, 103], 4 were duplicate cohorts [21, 30, 32, 33] and 4 had unclear capsular management [10, 50, 68, 105]. Ultimately, 47 studies [21, 22, 23, 24, 25, 26, 27, 28, 29, 30, 31, 32, 33, 34, 35, 36, 37, 38, 39, 40, 41, 42, 43, 44, 45, 46, 47, 48, 49, 50, 51, 52, 53, 54, 55, 56, 57, 58, 59, 60, 61, 62, 63, 64, 65, 66, 67] were included (Figure 1).

### Study characteristics

The 47 studies represented diverse countries and designs. Most originated from the United States (*n* = 32), followed by China (*n* = 5), and others from Israel, Netherlands, France, Italy, Denmark, Turkey, Spain and Australia. Among them, 5 were RCTs (Level I), 4 prospective cohorts (Level II), 27 retrospective cohorts (Level III), and 11 case series (Level IV). Thus, the evidence base was dominated by retrospective designs with relatively few RCTs. Most studies addressed FAI as the main indication, while 21 also focused on labral lesions. Less common indications included adhesions (1 study) and hip micro‐instability (1 study).

### Patient characteristics

In total, 7366 hips from 7276 patients were analyzed. The mean age was 34 years (range: 17–50) and 45% were male. The mean body mass index (BMI) was 25 kg/m^2^ (range: 18–40). By group: CP included 1,352 hips, mean age 36, 56% male, BMI 26; CR included 5043 hips, mean age 33, 42% male, BMI 25; CU included 971 hips, mean age 35, 46% male, BMI 24. More details on study and patient characteristics are presented in Tables 2 and 3.

**Table 2:** Study and patient characteristics.

Author	Origin	Journal	Study design	Level of evidence	Indication for HAS
Atzmon et al. [3]	Israel	*Journal of Hip Preservation Surgery*	Retrospective cohort study	III	FAI, labral lesion
Bech et al. [6]	Netherlands	*Hip International*	RCT	I	FAI, labral lesion
Bloom et al. [8]	USA	*Arthroscopy: The Journal of Arthroscopic and Related Surgery*	RCT	I	FAI
Bolia et al. [9]	USA	*The Journal of Arthroscopic and Related Surgery*	Retrospective cohort study	III	FAI, labral lesion
Bonin et al. [11]	France	*The Journal of Arthroscopic and Related Surgery*	Prospective cohort study	II	FAI
Chahla et al. [14]	USA	*American Journal of Sports Medicine*	Retrospective cohort study	III	FAI
Chambers et al. [15]	USA	*The Journal of Arthroscopic and Related Surgery*	Retrospective case series	IV	FAI
Cong et al. [20]	China	*Journal of Clinical Medicine*	Retrospective cohort study	III	FAI, labral lesion
Cvetanovich et al. [22]	USA	*American Journal of Sports Medicine*	Retrospective cohort study	III	FAI
Di Benedetto et al. [27]	Italy	*Acta Biomedica*	Retrospective cohort study	III	FAI
Dippmann et al. [28]	Denmark	*Knee Surg Sports Traumatol Arthrosc*	Prospective case series	IV	FAI
Domb et al. [31]	USA	*Arthroscopy: The Journal of Arthroscopic and Related Surgery*	Retrospective cohort study	III	FAI
Eberlin et al. [34]	USA	*The Orthopaedic Journal of Sports Medicine*	Retrospective cohort study	III	FAI, labral lesion
Economopoulos et al. [35]	USA	*The American Journal of Sports Medicine*	RCT	I	FAI, labral lesion
Firat et al. [38]	Turkey	*Medicina*	Retrospective cohort study	III	FAI
Frank et al. [41]	USA	*The American Journal of Sports Medicine*	Retrospective cohort study	III	FAI
Gao et al. [43]	China	*Journal of Orthopaedic Surgery and Research*	Retrospective cohort study	III	FAI
Hasselbrock et al. [46]	USA	*The American Journal of Sports Medicine*	Retrospective cohort study	III	FAI, labral lesion
Hunter et al. [49]	Australia	*BMJ Open*	RCT	I	FAI, labral lesion
Krych et al. [52]	USA	*Arthroscopy: The Journal of Arthroscopic and Related Surgery*	RCT	I	FAI, labral lesion
Larson et al. [53]	USA	*The American Journal of Sports Medicine*	Prospective case series	IV	FAI
Larson et al. [54]	USA	*Arthroscopy: The Journal of Arthroscopic and Related Surgery*	Retrospective cohort study	III	FAI
Levy et al. [55]	USA	*The American Journal of Sports Medicine*	Retrospective case series	IV	FAI
Li et al. [56]	China	*China J Ortop Trauma*	Retrospective cohort study	III	FAI, labral lesion
Li et al. [57]	China	*Orthopaedic Surgery*	Retrospective cohort study	III	FAI, labral lesion
Lv et al. [62]	China	*BMC Musculoskeletal Disorders*	Retrospective cohort study	III	FAI
Marland et al. [64]	USA	*The American Journal of Sports Medicine*	Retrospective case series	IV	FAI
Martin et al. [65]	USA	*The American Journal of Sports Medicine*	Retrospective case series	IV	Labral lesion
Matsuda et al. [66]	USA	*The American Journal of Orthopedics*	Retrospective cohort study	III	FAI, labral lesion
McGovern et al. [69]	USA	*Arthroscopy: The Journal of Arthroscopic and Related Surgery*	Retrospective cohort study	III	FAI, labral lesion
Morris et al. [70]	USA	*Arthroscopy, Sports Medicine, and Rehabilitation*	Retrospective cohort study	III	FAI
Mygind‐Klavsen et al. [71]	Denmark	*Journal of Hip Preservation Surgery*	Retrospective cohort study	III	FAI
Nguyen et al. [73]	USA	*Arthroscopy*	Prospective cohort study	II	FAI
Nho et al. [74]	USA	*The American Journal of Sports Medicine*	Prospective case series	IV	FAI
Özbek et al. [76]	Turkey	*Acta orthopaedica et traumatologica turcica*	Retrospective cohort study	III	FAI, labral lesion
Palmer et al. [77]	USA	*Arthroscopy: The Journal of Arthroscopic and Related Surgery*	Prospective case series	IV	FAI, labral lesion
Parvaresh et al. [80]	USA	*The American Journal of Sports Medicine*	Retrospective cohort study	III	FAI
Philippon et al. [82]	USA	*The Journal of Bone and Joint Surgery*	Prospective case series	IV	FAI
Philippon et al. [83]	USA	*The American Journal of Sports Medicine*	Retrospective cohort study	III	FAI, labral lesion
Ruzbarsky et al. [87]	USA	*The American Journal of Sports Medicine*	Prospective cohort study	II	Adhesions
Saltzman et al. [88]	USA	*The American Journal of Sports Medicine*	Retrospective case series	IV	FAI, labral lesion
Skendzel et al. [91]	USA	*The American Journal of Sports Medicine*	Retrospective cohort study	III	FAI
Sugarman et al. [94]	USA	*The Orthopaedic Journal of Sports Medicine*	Prospective cohort study	II	FAI, labral lesion
Tahoun et al. [95]	Spain	*KSSTA*	Retrospective cohort study	III	FAI, labral lesion
Umeh et al. [98]	USA	*Arthroscopy: The Journal of Arthroscopic and Related Surgery*	Retrospective cohort study	III	FAI, labral lesion
Vera et al. [99]	USA	*The Orthopaedic Journal of Sports Medicine*	Retrospective cohort study	III	FAI
Wylie et al. [101]	USA	*The American Journal of Sports Medicine*	Retrospective case series	IV	Micro‐instability

Abbreviations: FAI, femoroacetabular impingement; HAS, hip arthroscopy; RCT, randomized controlled trial.

**Table 3:** Patient characteristics.

Author	Capsular management	Description of capsule opening	Description of capsule repair	Patients (hips), *N*	Age, years ± SD (range)	Male sex (%)	BMI, kg/m^2^ ± SD (range)
Atzmon et al. [3]	CR	Linear interportal incision	Side‐to‐side suture‐closure	35 (35)	38 ± 2	60	NR
	CU	Linear interportal incision	–	29 (29)	38 ± 3	55	NR
Bech et al. [6]	CU	Linear interportal incision	–	58 (58)	35 ± 10 (18–65)	40	23.1 ± 2.7
	CR	Linear interportal incision	Side‐to‐side suture‐closure	58 (58)	33 ± 8 (18–65)	33	24.2 ± 2.9
Bloom et al. [8]	CR	Linear interportal incision	Side‐to‐side suture‐closure	40 (40)	40 ± 16 (35–45)	45	25.9 ± 4.0 (24.6–27.1)
	CR	Linear interportal incision	Side‐to‐side suture‐closure	40 (40)	39 ± 13 (35–44)	40	26.5 ± 5.1 (24.9–28.1)
Bolia et al. [9]	CR	Linear interportal incision	Side‐to‐side suture‐closure	84 (84)	38 ± 15	57	NR
	CU	Linear interportal incision	–	42 (42)	38 ± 15	57	NR
Bonin et al. [11]	CR	Linear interportal incision	Side‐to‐side suture‐closure	45 (49)	28 ± 7 (14–50)	90	23.5 ± 3.0 (19.6–31.9)
	CU	Linear interportal incision	–	45 (51)	30 ± 8 (14–57)	86	23.4 ± 1.9 (19.8–29.2)
Chahla et al. [14]	CR	T‐shaped capsulotomy	Side‐to‐side suture‐closure	267 (267)	32 ± 12	18	25.0 ± 12.5
	CR	T‐shaped capsulotomy	Side‐to‐side suture‐closure	333 (333)	35 ± 12	50	25.7 ± 5.1
Chambers et al. [15]	CP	Portal dilation	–	142 (142)	35 ± 12	51	25.3 ± 4.1
Cong et al. [20]	CR	T‐shaped capsulotomy	Side‐to‐side/shoelace sutures	22 (22)	38 ± 13	36	NR
Cvetanovich et al. [22]	CR	T‐shaped capsulotomy	Side‐to‐side suture‐closure	386 (414)	33 ± 12	42	25.3 ± 4.9
Di Benedetto et al. [27]	CU	Linear incision femoral neck	–	25 (25)	26 (15–35)	48	NR
	CR	Linear incision femoral neck	Side‐to‐side suture‐closure	25 (25)	26 (15–35)	48	NR
Dippmann et al. [28]	CP	–	–	87 (87)	38 (15–63)	37	NR
Domb et al. [31]	CR	Linear interportal incision/T‐shaped capsulotomy	Side‐to‐side suture‐plication	65 (65)	38 ± 13	28	24.4 ± 3.8
	CU	Linear interportal incision/T‐shaped capsulotomy	–	65 (65)	37 ± 12	28	24.1 ± 3.3
Eberlin et al. [34]	CP	Puncture capsulotomy	–	163 (163)	38 (36–40)	48	25.9 (25.2–26.5)
Economopoulos et al. [35]	CU	Linear interportal incision	–	50 (50)	39 ± 13	60	26.5 ± 4.2
	CU	T‐shaped capsulotomy	–	50 (50)	36 ± 13	56	25.8 ± 4.3
	CR	Linear interportal incision	Side‐to‐side suture‐closure	50 (50)	35 ± 11	62	24.8 ± 3.9
Firat et al. [38]	CU	Linear interportal incision	–	90 (90)	38 ± 10 (17‐60)	59	25.2 ± 2.9 (17.0–32.0)
Frank et al. [41]	CR	Linear interportal incision and T‐shaped capsulotomy	Side‐to‐side suture‐closure	32 (32)	33 ± 10	37	25.0 ± 3.2 (16.5–62.7)
	CU	Linear interportal incision and T‐shaped capsulotomy	Only partial repair	32 (32)	33 ± 10	37	24.7 ± 3.8 (16.5–62.7)
Gao et al. [43]	CR	Linear interportal incision	Side‐to‐side suture‐closure	194 (194)	37 (15–65)	45	23.1 (16.0–35.7)
Hasselbrock et al. [46]	CR	Linear interportal incision	Side‐to‐side suture‐closure	62 (62)	19 ± 4	71	24.6 ± 4.3
	CU	Linear interportal incision	–	49 (49)	19 ± 3	65	23.2 ± 3.1
Hunter et al. [49]	CP	–	–	49 (49)	33 ± 12	63	NR
Krych et al. [52]	CR	T‐shaped capsulotomy	Side‐to‐side suture‐closure	18 (18)	38 (20–59)	0	NR
	CR	T‐shaped capsulotomy	Side‐to‐side suture‐closure	18 (18)	39 (19–55)	0	NR
Larson et al. [53]	CR	Linear interportal incision	Side‐to‐side suture‐plication	79 (85)	29 (16–59)	44	NR
	CR	Linear interportal incision	Side‐to‐side suture‐plication	220 (237)	31	48	NR
Larson et al. [54]	CR	T‐shaped capsulotomy	Side‐to‐side suture‐closure	122 (122)	36 ± 12	0	24.9 ± 3.8
	CR	T‐shaped capsulotomy	Side‐to‐side suture‐closure	122 (122)	36 ± 11	100	25.7 ± 3.6
Levy et al. [55]	CR	T‐shaped capsulotomy	Side‐to‐side suture‐plication	51 (57)	26 ± 8	43	23.7 ± 3.3
Li et al. [56]	CR	Linear interportal incision	Side‐to‐side suture‐closure	17 (17)	32 ± 8 (20‐49)	59	NR
	CU	Linear interportal incision	–	17 (17)	31 ± 6 (20–49)	65	NR
Li et al. [57]	CP	Periportal capsulotomy	–	23 (23)	41 (27–67)	NR	NR
	CU	Linear interportal incision	–	10 (10)	41 (27–67)	NR	NR
Lv et al. [62]	CR	Linear interportal incision	Modified shoelace continuous suture	50 (50)	41 ± 2	38	23.1 ± 4.2
	CU	Linear interportal incision	–	50 (50)	45 ± 2	36	22.6 ± 4.7
Marland et al. [64]	CR	T‐shaped capsulotomy	Side‐to‐side suture‐closure	249 (249)	33 ± 11 (14–59)	0	25.0 ± 5.8 (15.0–58.0)
Martin et al. [65]	CP	Puncture capsulotomy	–	46 (46)	50 (48–51)	50	27.1 (25.8–28.4)
Matsuda et al. [66]	CU	T‐shaped capsulotomy	–	69 (69)	39	54	NR
	CU	T‐shaped capsulotomy	–	8 (8)	50	37	NR
McGovern et al. [69]	CP	Portal dilation	–	60 (60)	33 ± 14	17	25.2 ± 4.2
	CR	Linear interportal incision	Side‐to‐side suture‐closure	68 (68)	29 ± 9	62	26.1 ± 4.0
Morris et al. [70]	CR	Linear interportal incision	Side‐to‐side suture‐closure	557 (557)	34	53	NR
	CR	Linear interportal incision	Side‐to‐side suture‐closure	180 (180)	35	59	NR
Mygind‐Klavsen et al. [71]	CR	Linear interportal incision	Side‐to‐side suture‐closure	189 (189)	39 ± 12	53	NR
	CU	Linear interportal incision	–	189 (189)	39 ± 11	47	NR
Nguyen et al. [73]	CP	Portal dilation	–	28 (28)	30 ± 6	47	24.7 ± 3.1
Nho et al. [74]	CR	T‐shaped capsulotomy	Side‐to‐side suture‐closure	47 (47)	23 ± 6 (17–56)	72	NR
Özbek et al. [76]	CP	Portal widening	–	34 (34)	32 ± 12	41	24.9 ± 4.2
Palmer et al. [77]	CP	–	–	185 (201)	40 (14–87)	54	NR
Parvaresh et al. [80]	CR	T‐shaped capsulotomy	Side‐to‐side suture‐closure	329 (329)	33 ± 12	34	24.0 ± 3.7
	CR	Linear interportal incision	Side‐to‐side suture‐closure	329 (329)	32 ± 12	34	24.0 ± 3.7
Philippon et al. [82]	CP	–	–	112 (112)	41 (38–43)	21	(23.2–25.0)
Philippon et al. [83]	CP	–	–	28 (28)	27 (18–37)	100	NR
Rubarsky et al. [87]	CP	–	–	53 (56)	32 ± 11	45	NR
Saltzman et al. [88]	CR	Linear interportal incision/T‐shaped capsulotomy	Side‐to‐side suture‐closure	7 (7)	25 ± 14	71	17.9 ± 0.7
	CR	Linear interportal incision/T‐shaped capsulotomy	Side‐to‐side suture‐closure	197 (197)	30 ± 11	28	22.1 ± 1.7
	CR	Linear interportal incision/T‐shaped capsulotomy	Side‐to‐side suture‐closure	130 (130)	35 ± 12	57	26.9 ± 1.4
	CR	Linear interportal incision/T‐shaped capsulotomy	Side‐to‐side suture‐closure	31 (31)	42 ± 11	39	32.0 ± 1.1
	CR	Linear interportal incision/T‐shaped capsulotomy	Side‐to‐side suture‐closure	16 (16)	37 ± 10	31	39.9 ± 3.6
Skendzel et al. [91]	CP	–	–	323 (323)	37 (18–70)	70	NR
Sugarman et al. [94]	CR	Linear interportal incision/T‐shaped capsulotomy	Side‐to‐side suture‐closure	28 (28)	32 ± 9	21	24.4 ± 4.9
	CU	Linear interportal incision/T‐shaped capsulotomy	–	28 (28)	34 ± 10	71	25.7 ± 4.1
Tahoun et al. [95]	CR	Linear interportal incision and T‐shaped capsulotomy	Side‐to‐side suture‐closure	44 (44)	NR	NR	NR
	CU	Linear interportal incision and T‐shaped capsulotomy	–	42 (42)	NR	NR	NR
Umeh et al. [98]	CR	Linear interportal incision/T‐shaped capsulotomy	Side‐to‐side suture‐plication	51 (51)	36 ± 10	37	26.2 ± 5.2
	CR	Linear interportal incision/T‐shaped capsulotomy	Side‐to‐side suture‐plication	52 (52)	36 ± 11	40	26.4 ± 5.1
Vera et al. [99]	CR	Linear interportal incision	Side‐to‐side suture‐closure	20 (23)	18 ± 2 (16–19)	0	20.7 ± 3.3
	CU	Linear interportal incision	–	16 (17)	17 ± 2 (15–20)	0	22.4 ± 3.6
Wylie et al. [101]	CR	Linear interportal incision	Side‐to‐side suture‐closure	20 (20)	30 (15–56)	10	NR

Abbreviations: BMI, body mass index; CP, capsule preserved; CR, capsule repaired; CU, capsule unrepaired; NR, not reported; SD, standard deviation.

### Study quality and bias assessment

Fifteen studies were rated low RoB, while 32 were moderate (Table 4). Non‐RCTs most often showed concerns for confounding and reporting, but classification of interventions and outcome measures was usually reliable. All RCTs were low risk across domains, supporting internal validity (Table 4, Figure 2). Publication bias varied across outcomes (Figures S1–S24). Several funnel plots appeared symmetrical (e.g., post‐operative iHOT, NAHS and MCID at 12 months), but others showed clear asymmetry (e.g., HOS‐SSS, VAS change, complications, revisions and THA conversion). Many plots showed mild to moderate asymmetry, suggesting possible small‐study effects or selective reporting. Overall, functional benefits may be overestimated, and complications underreported.

**Table 4:** Risk of bias (ROB) assessment using the ROBINS‐I tool for non‐RCTs and the RoB‐2 tool for RCTs.

Study	Bias due to confounding	Bias in selection of participants	Bias in classification of interventions	Bias due to deviations from intended interventions	Bias due to missing data	Bias in measurement of outcomes	Bias in selection of the reported result	Overall RoB
Atzmon et al. [3]	Moderate	Low	Low	Low	Moderate	Low	Moderate	Moderate
Bolia et al. [9]	Moderate	Low	Low	Low	Low	Low	Moderate	Moderate
Bonin et al. [11]	Low	Low	Low	Low	Low	Low	Low	Low
Chahla et al. [14]	Moderate	Low	Low	Low	Low	Low	Moderate	Moderate
Chambers et al. [15]	Moderate	Low	Low	Low	Moderate	Low	Moderate	Moderate
Cong et al. [20]	Serious	Moderate	Low	Low	Moderate	Low	Moderate	Moderate
Cvetanovich et al. [22]	Moderate	Low	Low	Low	Low	Low	Moderate	Moderate
Di Benedetto et al. [27]	Moderate	Low	Low	Low	Low	Low	Moderate	Moderate
Dippmann et al. [28]	Moderate	Low	Low	Low	Moderate	Low	Moderate	Moderate
Domb et al. [31]	Moderate	Low	Low	Low	Low	Low	Moderate	Moderate
Eberlin et al. [34]	Moderate	Low	Low	Low	Low	Low	Moderate	Moderate
Firat et al. [38]	Moderate	Low	Low	Low	Low	Low	Moderate	Moderate
Frank et al. [41]	Moderate	Low	Low	Low	Moderate	Low	Moderate	Moderate
Gao et al. [43]	Moderate	Low	Low	Low	Low	Low	Moderate	Moderate
Hasselbrock et al. [46]	Moderate	Low	Low	Low	Low	Low	Moderate	Moderate
Larson et al. [53]	Low	Low	Moderate	Low	Low	Low	Low	Low
Larson et al. [54]	Low	Low	Low	Low	Low	Low	Low	Low
Levy et al. [55]	Low	Low	Moderate	Low	Low	Low	Low	Low
Li et al. [56]	Low	Low	Low	Moderate	Low	Low	Low	Low
Li et al. [57]	Low	Low	Low	Moderate	Low	Low	Low	Low
Lv et al. [62]	Low	Low	Low	Low	Low	Low	Low	Low
Marland et al. [64]	Low	Low	Low	Low	Low	Low	Low	Low
Martin et al. [65]	Moderate	Low	Low	Moderate	Low	Low	Low	Moderate
Matsuda et al. [66]	Moderate	Low	Low	Moderate	Low	Low	Moderate	Moderate
McGovern et al. [69]	Moderate	Low	Low	Moderate	Low	Low	Moderate	Moderate
Morris et al. [70]	Moderate	Low	Low	Moderate	Low	Low	Moderate	Moderate
Mygind‐Klavsen et al. [71]	Moderate	Low	Low	Moderate	Low	Low	Moderate	Moderate
Nguyen et al. [73]	Moderate	Low	Low	Low	Low	Low	Moderate	Moderate
Nho et al. [74]	Moderate	Low	Low	Low	Low	Low	Moderate	Moderate
Özbek et al. [76]	Moderate	Low	Low	Low	Moderate	Low	Moderate	Moderate
Palmer et al. [77]	Moderate	Low	Low	Low	Moderate	Low	Moderate	Moderate
Parvaresh et al. [80]	Moderate	Low	Low	Low	Moderate	Low	Moderate	Moderate
Philippon et al. [82]	Moderate	Low	Low	Low	Moderate	Low	Moderate	Moderate
Philippon et al. [83]	Moderate	Low	Low	Low	Moderate	Low	Moderate	Moderate
Ruzbarsky et al. [87]	Moderate	Low	Low	Moderate	Low	Low	Moderate	Moderate
Saltzman et al. [88]	Moderate	Low	Low	Low	Moderate	Low	Moderate	Moderate
Skendzel et al. [91]	Moderate	Low	Low	Moderate	Moderate	Low	Moderate	Moderate
Sugarman et al. [94]	Low	Low	Low	Low	Low	Low	Low	Low
Tahoun et al. [95]	Moderate	Low	Low	Moderate	Low	Low	Low	Moderate
Umeh et al. [98]	Low	Low	Low	Moderate	Low	Low	Low	Moderate
Vera et al. [99]	Low	Low	Low	Low	Low	Low	Low	Low
Wylie et al. [101]	Moderate	Low	Low	Low	Moderate	Low	Moderate	Moderate

Study	Bias arising from the randomization process	Bias due to deviations from intended interventions	Bias due to missing outcome data	Bias in measurement of the outcome	Bias in selection of the reported result	Overall RoB
Bech et al. [6]	Low	Low	Low	Low	Low	Low
Bloom et al. [8]	Low	Low	Low	Low	Low	Low
Economopoulos et al. [35]	Low	Low	Low	Low	Low	Low
Hunter et al. [49]	Low	Low	Low	Low	Low	Low
Krych et al. [52]	Low	Low	Low	Low	Low	Low

Abbreviations: RCT, randomized controlled trial; ROBINS‐I, Risk Of Bias In Non‐randomized Studies of Interventions.

**Figure 2:**
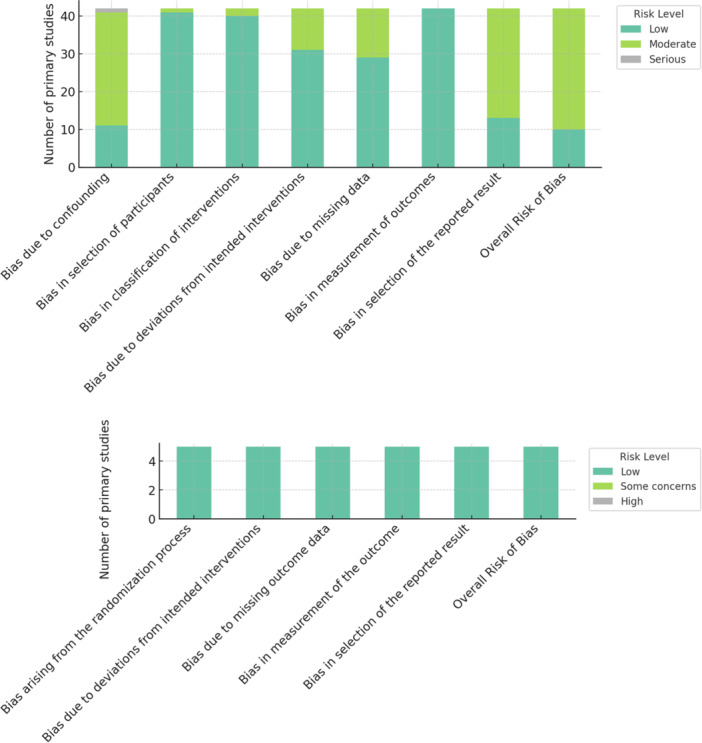
Graphical summary risk of bias assessment.

### Capsular management strategies

- CP (periportal only): 18.3% of patients; small perforations limited to instrument width; included periportal, dilatation, or puncture capsulotomy.
- CR (formal capsulotomy with closure): 68.4% of patients; openings 2–6 cm; interportal or T‐shaped capsulotomy closed with 1–8 sutures.
- CU (formal capsulotomy without closure): 13.2% of patients; openings 2–6 cm left unrepaired.

### Post‐operative total outcome parameters

Post‐operative mHHS total was reported in 50 studies with 4434 hips (Table 5) (Figure S25). The pooled mean was 83.06 (95% CI: 80.87–85.25). The mean in the CP group was 82.85 (95% CI: 78.68–87.03), in the CR group 83.89 (95% CI: 81.18–86.60), and in the CU group 81.35 (95% CI: 78.27–84.44). The difference between groups was not significant (*p* = 0.1617).

**Table 5:** Summary of the post‐operative outcome parameters.

	Primary studies, *N*	Hips, *N*	Mean value	CIs	*τ* ^2^	*I* ^2^	Heterogenity *p*	Difference *p*
Post‐operative overall mHHS								
Total	50	4434	83.06	80.87–85.25	34.92	1.00	<0.0001***	0.1617
CP	9	691	82.85	78.68–87.03	37.07	0.98	<0.0001***	
CR	28	3187	83.89	81.18–86.6	37.07	0.99	<0.0001***	
CU	13	556	81.35	78.27–84.44	37.07	0.99	<0.0001***	
Post‐operative overall iHOT								
Total	14	1693	72.36	68–76.71	31.99	0.88	<0.0001***	0.5643
CP	4	318	71.09	65.02–77.15	30.10	0.00	0.6667	
CR	8	1296	72.61	67.61–77.6	30.10	0.92	<0.0001***	
CU	2	79	75.50	67.77–83.23	30.10	0.47	0.1701	
Post‐operative overall HOS ADL								
Total	38	3181	86.31	84.69–87.92	16.63	0.87	<0.0001***	0.2561
CP	6	460	86.19	82.54–89.84	16.24	0.49	0.0825	
CR	23	2393	87.22	85.16–89.27	16.24	0.84	<0.0001***	
CU	9	328	84.46	81.47–87.45	16.24	0.88	<0.0001***	
Post‐operative overall HOS SSS								
Total	38	3198	76.75	74.31–79.18	28.36	0.93	<0.0001***	0.0598
CP	5	437	72.65	67.66–77.65	24.90	0.66	0.0199*	
CR	24	2418	78.34	75.69–80.99	24.90	0.92	<0.0001***	
CU	9	343	76.06	72.69–79.44	24.90	0.95	<0.0001***	
Post‐operative overall NAHS								
Total	9	616	79.38	72.89–85.86	59.67	0.89	<0.0001***	0.4837
CP	3	359	80.66	68.36–92.97	73.49	0.61	0.0789	
CR	2	90	84.50	69.44–99.56	73.49	0.00	0.7156	
CU	4	167	75.15	64.19–86.11	73.49	0.92	<0.0001***	
Post‐operative overall VAS								
Total	36	4099	1.84	1.62–2.06	0.25	0.96	<0.0001***	0.6459
CP	8	756	1.98	1.61–2.36	0.26	0.99	<0.0001***	
CR	22	3083	1.77	1.49–2.06	0.26	0.91	<0.0001***	
CU	6	260	1.83	1.42–2.24	0.26	0.77	0.0005***	

Abbreviations: CI, confidence interval; CP, capsule preserved; CR, capsule repaired; CU, capsule unrepaired; HOS‐ADL, Hip Outcome Score – Activities of Daily Living; HOS‐SSS, Hip Outcome Score – Sports Subscale; iHOT, International Hip Outcome Tool; mHHS, modified Harris Hip Score; NAHS, Non‐Arthritic Hip Score; VAS, visual analogue score.

* Statistically significant.

** Very statistically significant.

*** Highly statistically significant.

*Post‐operative iHOT total* was reported in 14 studies with 1693 hips (Table 5) (Figure S26). The pooled mean was 72.36 (95% CI: 68.00–76.71). The mean in the CP group was 71.09 (95% CI: 65.02–77.15), in the CR group 72.61 (95% CI: 67.61–77.60), and in the CU group 75.50 (95% CI: 67.77–83.23). The difference between groups was not significant (*p* = 0.5643).

*Post‐operative HOS‐ADL total* was reported in 38 studies with 3181 hips (Table 5) (Figure S27). The pooled mean was 86.31 (95% CI: 84.69–87.92). The mean in the CP group was 86.19 (95% CI: 82.54–89.84), in the CR group 87.22 (95% CI: 85.16–89.27) and in the CU group 84.46 (95% CI: 81.47–87.45). The difference between groups was not significant (*p* = 0.2561).

*Post‐operative HOS‐SSS total* was reported in 38 studies with 3198 hips (Table 5) (Figure S28). The pooled mean was 76.75 (95% CI: 74.31–79.18). The mean in the CP group was 72.65 (95% CI: 67.66–77.65), in the CR group 78.34 (95% CI: 75.69–80.99) and in the CU group 76.06 (95% CI: 72.69–79.44). The difference between groups was not significant (*p* = 0.0598).

*Post‐operative NAHS total* was reported in nine studies with 616 hips (Table 5) (Figure S29). The pooled mean was 79.38 (95% CI: 72.89–85.86). The mean in the CP group was 80.66 (95% CI: 68.36–92.97), in the CR group 84.50 (95% CI: 69.44–99.56) and in the CU group 75.15 (95% CI: 64.19–86.11). The difference between groups was not significant (*p* = 0.4837).

*Post‐operative VAS total* was reported in 36 studies with 4099 hips (Table 5) (Figure S30). The pooled mean was 1.84 (95% CI: 1.62–2.06). The mean in the CP group was 1.98 (95% CI: 1.61–2.36), in the CR group 1.77 (95% CI: 1.49–2.06) and in the CU group 1.83 (95% CI: 1.42–2.24). The difference between groups was not significant (*p* = 0.6459).

### Pre‐ to post‐operative change

**Figure 3:**
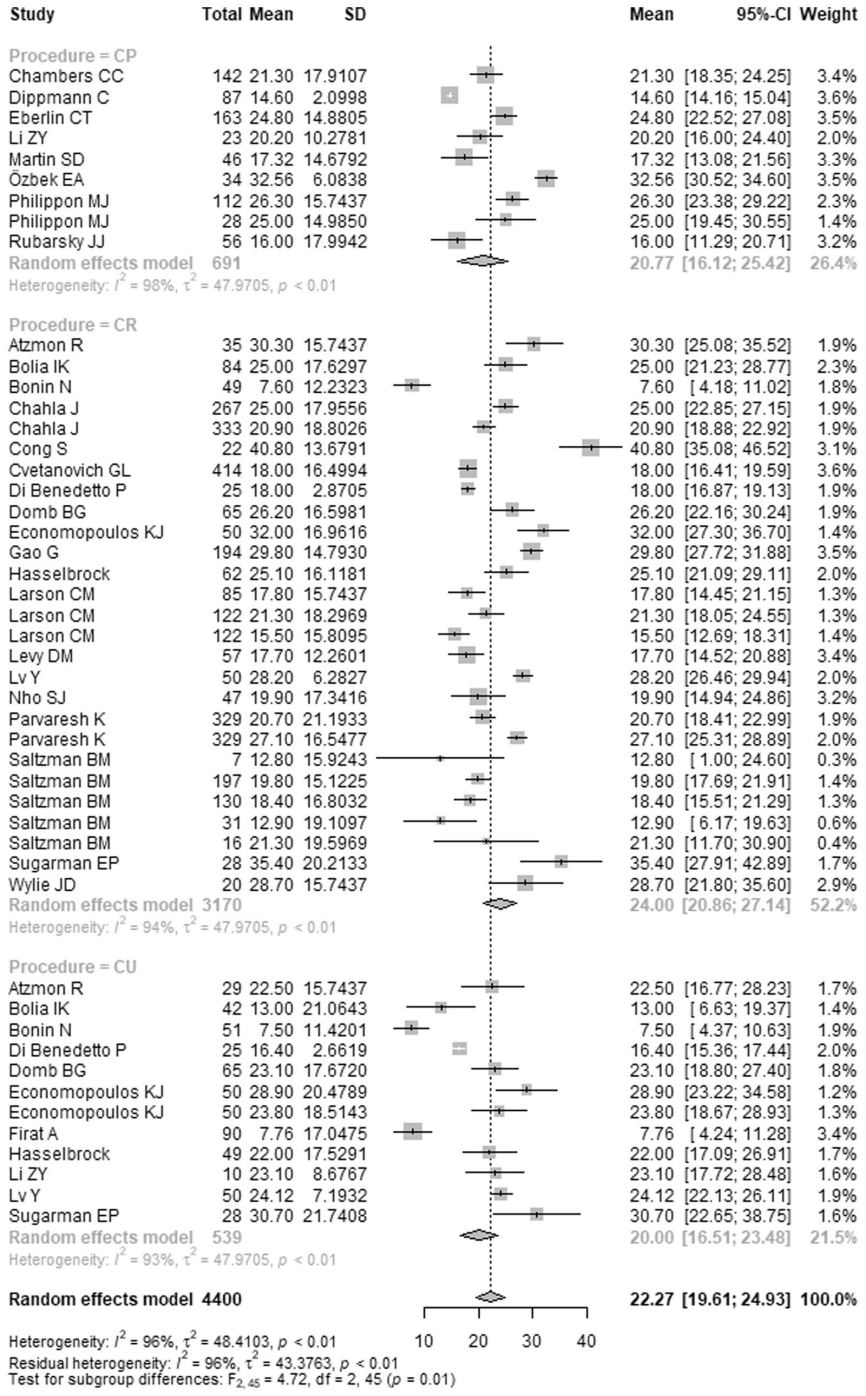
Forest plot of the change in mHHS. CI, confidence interval; CP, capsule preserved; CR, capsule repaired; CU, capsule unrepaired; SD, standard deviation; mHHS, modified Harris Hip Score.

*Change in mHHS* was reported in 48 studies with 4400 hips (Table 6) (Figure 3). The pooled mean difference was 22.27 (95% CI: 19.61–24.93). The mean difference in the CP group was 20.77 (95% CI: 16.12–25.42), in the CR group 24.00 (95% CI: 20.86–27.14) and in the CU group 20.00 (95% CI: 16.51–23.48). The difference between groups was statistically significant (*p* = 0.0137).

**Table 6:** Summary of pre‐ to post‐operative changes in outcome parameters.

	Primary studies, *N*	Hips, *N*	Mean value	CIs	*τ* ^2^	*I* ^2^	Heterogenity *p*	Difference *p*
Change in mHHS								
Total	48	4400	22.27	19.61–24.93	48.41	0.96	<0.0001***	0.0137*
CP	9	691	20.77	16.12–25.42	47.97	0.98	<0.0001***	
CR	27	3170	24.00	20.86–27.14	47.97	0.94	<0.0001***	
CU	12	539	20.00	16.51–23.48	47.97	0.93	<0.0001***	
Change in iHOT								
Total	12	1449	34.21	30.62–37.8	20.41	0.91	<0.0001***	0.664
CP	4	318	33.32	27.83–38.81	23.22	0.91	<0.0001***	
CR	6	1052	34.25	29.4–39.1	23.22	0.93	<0.0001***	
CU	2	79	36.76	29.34–44.17	23.22	0.00	0.3476	
Change in HOS ADL								
Total	36	3079	22.29	19.18–25.4	43.97	0.94	<0.0001***	0.3202
CP	4	358	21.27	14.77–27.77	47.21	0.45	0.1405	
CR	23	2393	23.33	19.7–26.95	47.21	0.95	<0.0001***	
CU	9	328	20.18	15.59–24.77	47.21	0.92	<0.0001***	
Change in HOS SSS								
Total	37	3152	33.43	28.42–38.44	132.99	0.98	<0.0001***	0.2816
CP	4	391	28.89	16.91–40.86	134.92	0.55	0.0807	
CR	24	2418	35.73	29.84–41.62	134.92	0.99	<0.0001***	
CU	9	343	30.42	22.36–38.47	134.92	0.96	<0.0001***	
Change in NAHS								
Total	8	570	18.37	12.56–24.18	45.24	0.92	<0.0001***	0.6745
CP	2	313	18.56	4.08–33.05	62.44	0.96	<0.0001***	
CR	2	90	22.36	7.81–36.91	62.44	0.95	<0.0001***	
CU	4	167	15.92	5.35–26.48	62.44	0.91	<0.0001***	
Change in VAS								
Total	36	4099	−3.90	−4.26 to −3.54	0.86	0.97	<0.0001***	0.2608
CP	8	756	−3.89	−4.56 to −3.21	0.83	0.93	<0.0001***	
CR	22	3083	−4.08	−4.53 to −3.62	0.83	0.97	<0.0001***	
CU	6	260	−3.36	−4.14 to −2.58	0.83	0.83	<0.0001***	

Abbreviations: CI, confidence interval; CP, capsule preserved; CR, capsule repaired; CU, capsule unrepaired; HOS‐ADL, Hip Outcome Score – Activities of Daily Living; HOS‐SSS, Hip Outcome Score – Sports Subscale; iHOT, International Hip Outcome Tool; mHHS, modified Harris Hip Score; NAHS, Non‐Arthritic Hip Score; VAS, visual analogue score.

* Statistically significant.

** Very statistically significant.

*** Highly statistically significant.

*Change in iHOT* was reported in 12 studies with 1449 hips (Table 6) (Figure S31). The pooled mean difference was 34.21 (95% CI: 30.62–37.80). The mean difference in the CP group was 33.32 (95% CI: 27.83–38.81), in the CR group 34.25 (95% CI: 29.40–39.10) and in the CU group 36.76 (95% CI: 29.34–44.17). The difference between groups was not significant (*p* = 0.6640).

*Change in HOS‐ADL* was reported in 36 studies with 3079 hips (Table 6) (Figure S32). The pooled mean difference was 22.29 (95% CI: 19.18–25.40). The mean difference in the CP group was 21.27 (95% CI: 14.77–27.77), in the CR group 23.33 (95% CI: 19.70–26.95) and in the CU group 20.18 (95% CI: 15.59–24.77). The difference between groups was not significant (*p* = 0.3202).

*Change in HOS‐SSS* was reported in 37 studies with 3152 hips (Table 6) (Figure S33). The pooled mean difference was 33.43 (95% CI: 28.42–38.44). The mean difference in the CP group was 28.89 (95% CI: 16.91–40.86), in the CR group 35.73 (95% CI: 29.84–41.62) and in the CU group 30.42 (95% CI: 22.36–38.47). The difference between groups was not significant (*p* = 0.2816).

*Change in NAHS* was reported in eight studies with 570 hips (Table 6) (Figure S34). The pooled mean difference was 18.37 (95% CI: 12.56–24.18). The mean difference in the CP group was 18.56 (95% CI: 4.08–33.05), in the CR group 22.36 (95% CI: 7.81–36.91) and in the CU group 15.92 (95% CI: 5.35–26.48). The difference between groups was not significant (*p* = 0.6745).

*Change in VAS* was reported in 36 studies with 4099 hips (Table 6) (Figure S35). The pooled mean difference was −3.90 (95% CI: −4.26 to −3.54). The mean difference in the CP group was −3.89 (95% CI: −4.56 to −3.21), in the CR group −4.08 (95% CI: −4.53 to −3.62), and in the CU group −3.36 (95% CI: −4.14 to −2.58). The difference between groups was not significant (*p* = 0.2608).

### MCID post‐operative outcome parameters

*Functional MCID at 3 months post‐operatively* was reported in 16 studies with 846 hips (Table 7) (Figure S36). The pooled mean was 7.33 (95% CI: 5.71–8.96). The mean in the CP group was 8.82 (95% CI: 6.49–11.15), in the CR group 6.49 (95% CI: 4.56–8.41), and in the CU group 6.21 (95% CI: 4.28–8.13). The difference between groups was not significant (*p* = 0.1732).

**Table 7:** Summary of the MCID in post‐operative outcome parameters.

	Primary studies, *N*	Hips, *N*	Mean value	CIs	*τ* ^2^	*I* ^2^	Heterogenity *p*	Difference *p*
Functional MCID 3 months post‐operatively								
Total	16	846	7.33	5.71–8.96	5.83	1.00	<0.0001***	0.1732
CP	4	330	8.82	6.49–11.15	4.64	0.98	<0.0001***	
CR	5	215	6.49	4.56–8.41	4.64	0.99	<0.0001***	
CU	7	301	6.21	4.28–8.13	4.64	0.99	<0.0001***	
Functional MCID 6 months post‐operatively								
Total	20	1109	9.24	8.47–10	1.74	0.97	<0.0001***	0.376
CP	6	402	8.92	7.95–9.89	1.72	0.99	<0.0001***	
CR	7	438	9.58	8.66–10.51	1.72	0.23	0.2504	
CU	7	269	9.30	8.39–10.21	1.72	0.97	<0.0001***	
Functional MCID 12 months post‐operatively								
Total	30	2083	8.55	7.52–9.58	4.78	1.00	<0.0001***	0.0428*
CP	6	462	9.30	7.47–11.14	4.76	0.98	<0.0001***	
CR	13	1024	8.35	7.11–9.6	4.76	1.00	<0.0001***	
CU	11	597	7.99	6.73–9.24	4.76	1.00	<0.0001***	
Functional MCID 24 months post‐operatively								
Total	43	3888	9.71	9.22–10.2	1.37	0.96	<0.0001***	0.2007
CP	6	432	9.82	8.96–10.69	1.35	0.99	<0.0001***	
CR	25	2918	9.81	9.28–10.34	1.35	0.95	<0.0001***	
CU	12	538	9.43	8.84–10.03	1.35	0.94	<0.0001***	
Pain MCID last follow‐up								
Total	40	4318	1.02	0.89–1.15	0.09	0.97	<0.0001***	0.8416
CP	8	756	1.07	0.84–1.29	0.10	0.99	<0.0001***	
CR	25	3244	0.99	0.83–1.16	0.10	0.93	<0.0001***	
CU	7	318	1.03	0.8–1.26	0.10	0.79	<0.0001***	

Abbreviations: CI, confidence interval; CP, capsule preserved; CR, capsule repaired; CU, capsule unrepaired; MCID, minimal clinically important difference.

* Statistically significant.

** Very statistically significant.

*** Highly statistically significant.

*Functional MCID at 6 months post‐operatively* was reported in 20 studies with 1109 hips (Table 7) (Figure S37). The pooled mean was 9.24 (95% CI: 8.47–10.00). The mean in the CP group was 8.92 (95% CI: 7.95–9.89), in the CR group 9.58 (95% CI: 8.66–10.51) and in the CU group 9.30 (95% CI: 8.39–10.21). The difference between groups was not significant (*p* = 0.3760).

**Figure 4:**
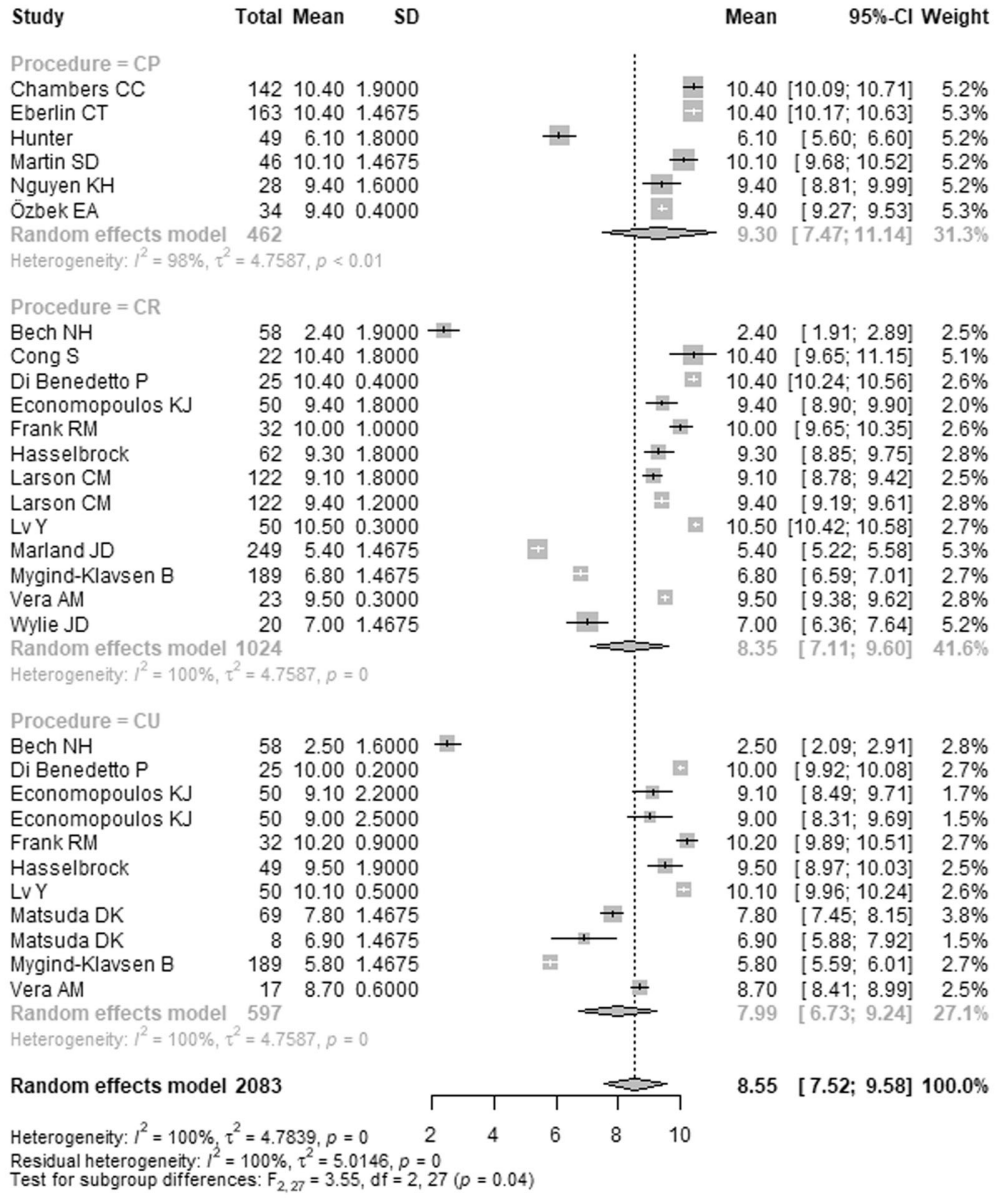
Forest plot of the functional MCID at 12 months postoperatively. CI, confidence interval; CP, capsule preserved; CR, capsule repaired; CU, capsule unrepaired; MCID, minimal clinically important difference; SD, standard deviation.

*Functional MCID at 12 months post‐operatively* was reported in 30 studies with 2083 hips (Table 7) (Figure 4). The pooled mean was 8.55 (95% CI: 7.52–9.58). The mean in the CP group was 9.30 (95% CI: 7.47–11.14), in the CR group 8.35 (95% CI: 7.11–9.60) and in the CU group 7.99 (95% CI: 6.73–9.24). The difference between groups was statistically significant (*p* = 0.0428).

*Functional MCID at 24 months postoperatively* was reported in 43 studies with 3888 hips (Table 7) (Figure S38). The pooled mean was 9.71 (95% CI: 9.22–10.20). The mean in the CP group was 9.82 (95% CI: 8.96–10.69), in the CR group 9.81 (95% CI: 9.28–10.34) and in the CU group 9.43 (95% CI: 8.84–10.03). The difference between groups was not significant (*p* = 0.2007).

*Pain MCID at last follow‐up* was reported in 40 studies with 4318 hips (Table 7) (Figure S39). The pooled mean was 1.02 (95% CI: 0.89–1.15). The mean in the CP group was 1.07 (95% CI: 0.84–1.29), in the CR group 0.99 (95% CI: 0.83–1.16) and in the CU group 1.03 (95% CI: 0.80–1.26). The difference between groups was not significant (*p* = 0.8416).

### Post‐operative complications

*Overall complications* were reported in 31 studies with 2219 hips (Table 8) (Figure S40). The pooled rate was 0.04 (95% CI: 0.03–0.08). The rate in the CP group was 0.07 (95% CI: 0.02–0.17), in the CR group 0.03 (95% CI: 0.02–0.07) and in the CU group 0.05 (95% CI: 0.02–0.10). The difference between groups was not significant (*p* = 0.3170).

**Table 8:** Summary of the post‐operative complications.

	Primary studies, *N*	Hips, *N*	Mean value	CIs	*τ* ^2^	*I* ^2^	Heterogenity *p*	Difference *p*
Overall complications								
Total	31	2219	0.04	0.03–0.08	1.16	0.73	<0.0001***	0.317
CP	5	583	0.07	0.02–0.17	1.07	0.91	<0.0001***	
CR	15	1152	0.03	0.02–0.07	1.07	0.19	0.2394	
CU	11	484	0.05	0.02–0.1	1.07	0.6	0.0052**	
DVT/PE								
Total	29	1953	0.01	0.01–0.02	0.00	0.00	0.9999	0.6784
CP	4	382	0.01	0.01–0.04	0.00	0.00	0.7339	
CR	14	1087	0.01	0–0.02	0.00	0.00	0.9898	
CU	11	484	0.01	0.01–0.03	0.00	0.00	0.9951	
Nerve injury								
Total	34	2553	0.03	0.02–0.05	1.15	0.75	<0.0001***	0.495
CP	5	583	0.03	0.01–0.11	1.14	0.92	<0.0001***	
CR	18	1486	0.02	0.01–0.04	1.14	0.19	0.2314	
CU	11	484	0.04	0.01–0.09	1.14	0.57	0.0090**	
Infection								
Total	32	2300	0.02	0.01–0.02	0.22	0	0.871	0.1677
CP	5	583	0.01	0–0.02	0.13	0	0.8729	
CR	17	1298	0.02	0.01–0.03	0.13	0	0.9766	
CU	10	419	0.03	0.01–0.06	0.13	0	0.476	
Haematoma								
Total	29	2089	0.01	0.01–0.02	0.00	0.00	0.999	0.2734
CP	5	583	0.01	0–0.02	0.00	0.00	0.8387	
CR	14	1087	0.01	0–0.02	0.00	0.00	0.9864	
CU	10	419	0.02	0.01–0.04	0.00	0.00	0.9938	
Revision								
Total	56	4925	0.04	0.02–0.06	1.24	0.73	<0.0001***	0.1686
CP	8	785	0.01	0–0.05	1.25	0.79	<0.0001***	
CR	30	3208	0.04	0.02–0.06	1.25	0.79	<0.0001***	
CU	18	932	0.05	0.03–0.08	1.25	0.47	0.0151*	
Converted to THA								
Total	42	4211	0.03	0.02–0.05	0.88	0.67	<0.0001***	0.4506
CP	8	789	0.03	0.01–0.07	0.91	0.57	0.0241*	
CR	20	2665	0.03	0.01–0.05	0.91	0.75	<0.0001***	
CU	14	757	0.04	0.02–0.08	0.91	0.52	0.0118*	

Abbreviations: CI, confidence interval; CP, capsule preserved; CR, capsule repaired; CU, capsule unrepaired; DVT, deep vein thrombosis; PE, pulmonary embolism; THA, total hip arthroplasty.

* Statistically significant.

** Very statistically significant.

*** Highly statistically significant.

*DVT/PE* was reported in 29 studies with 1953 hips (Table 8) (Figure S41). The pooled rate was 0.01 (95% CI: 0.01–0.02). The rate in the CP group was 0.01 (95% CI: 0.01–0.04), in the CR group 0.01 (95% CI: 0.00–0.02) and in the CU group 0.01 (95% CI: 0.01–0.03). The difference between groups was not significant (*p* = 0.6784).

*Nerve injury* was reported in 34 studies with 2553 hips (Table 8) (Figure S42). The pooled rate was 0.03 (95% CI: 0.02–0.05). The rate in the CP group was 0.03 (95% CI: 0.01–0.11), in the CR group 0.02 (95% CI: 0.01–0.04) and in the CU group 0.04 (95% CI: 0.01–0.09). The difference between groups was not significant (*p* = 0.4950).

*Infection* was reported in 32 studies with 2300 hips (Table 8) (Figure S43). The pooled rate was 0.02 (95% CI: 0.01–0.02). The rate in the CP group was 0.01 (95% CI: 0.00–0.02), in the CR group 0.02 (95% CI: 0.01–0.03) and in the CU group 0.03 (95% CI: 0.01–0.06). The difference between groups was not significant (*p* = 0.1677).

*Haematoma* was reported in 29 studies with 2089 hips (Table 8) (Figure S44). The pooled rate was 0.01 (95% CI: 0.01–0.02). The rate in the CP group was 0.01 (95% CI: 0.00–0.02), in the CR group 0.01 (95% CI: 0.00–0.02) and in the CU group 0.02 (95% CI: 0.01–0.04). The difference between groups was not significant (*p* = 0.2734).

*Revisions* were reported in 56 studies with 4925 hips (Table 8) (Figure S45). The pooled rate was 0.04 (95% CI: 0.02–0.06). The rate in the CP group was 0.01 (95% CI: 0.00–0.05), in the CR group 0.04 (95% CI: 0.02–0.06) and in the CU group 0.05 (95% CI: 0.03–0.08). The difference between groups was not significant (*p* = 0.1686).

*Conversion to THA* was reported in 42 studies with 4211 hips (Table 8) (Figure S46). The pooled rate was 0.03 (95% CI: 0.02–0.05). The rate in the CP group was 0.03 (95% CI: 0.01–0.07), in the CR group 0.03 (95% CI: 0.01–0.05) and in the CU group 0.04 (95% CI: 0.02–0.08). The difference between groups was not significant (*p* = 0.4506).

### Further results

There were no differences in preoperative functional scores (mHHS, iHOT, HOS‐ADL, HOS‐SSS and NAHS) or preoperative pain scores (VAS) between CP, CR and CU (Table S2) (Figures [Link], [Link], [Link], [Link], [Link], [Link]). Further detailed results of functional and pain scores at different post‐operative time points are provided in Table S2, with no relevant statistical differences observed between CP, CR and CU (Table S3) (Figures [Link], [Link], [Link], [Link], [Link], [Link], [Link], [Link], [Link], [Link], [Link], [Link], [Link], [Link], [Link], [Link], [Link], [Link], [Link], [Link], [Link]).

## DISCUSSION

This multilevel meta‐analysis included 47 studies and 7366 hips. It compared three capsular strategies (CP, CR and CU) during HAS for FAIS. Outcomes covered function, pain and safety. CP and CR showed better results than CU in selected domains: CR improved mHHS change most, while CP had the highest MCID rate at 12 months. Although limited to two parameters, these findings suggest that unrepaired capsules may delay recovery. Other outcomes showed no consistent group differences. This is the first analysis to compare all three strategies in one framework.

Overall, CP, CR and CU produced similar short‐ and mid‐term results for most outcomes. Yet CR achieved the greatest mHHS gain, and CP the best MCID rate. These signals indicate that CP and CR are more favourable than CU. Revision, THA conversion and complication rates were not significantly different. Thus, not all strategies are equivalent; CP and CR appear preferable when feasible. Between CP and CR, no consistent superiority emerged across outcomes. This suggests that the decision depends less on PROMs and more on surgical and patient factors. Exposure needs, technical feasibility, instability risk, tissue quality and capsule morphology should guide whether preservation or repair is chosen. Anatomical factors such as capsular thickness, morphology and tissue quality may further influence the feasibility and stability of preservation or repair. Because most included studies focused on FAIS in relatively young adults, generalizability to patients with dysplasia, generalized laxity or older populations remains limited.

The present findings align with earlier analyses by Liu et al. [59] and Lin et al. [58], who found no significant differences between capsular closure and non‐closure across core functional outcomes and revision risk. Lin et al. [58] also concluded that CR was not routinely necessary. However, later studies such as those by Looney et al. [61], Cohen et al. [18] and Dasari et al. [25] emphasized improved outcomes with CR. Carbone et al. similarly reported that repair improved patient‐reported outcomes and survivorship, although their analysis was limited by small numbers and non‐randomized designs [12]. Shen et al. confirmed superior results with repair in patients without dysplasia or generalized ligamentous laxity, but did not account for capsule‐preserving approaches [90]. These differences in interpretation may stem from variations in patient selection, methodological design, or analytical scope. Importantly, all prior reviews were restricted to repair versus non‐repair and did not include CP as a distinct comparator. In contrast, the present study explicitly incorporates CP alongside CR and CU and applies a multilevel model that accounts for both within‐ and between‐study variance. This broader approach revealed that CP and CR were associated with superior outcomes compared to CU, thereby providing a more differentiated and clinically relevant perspective on capsular management. This approach revealed that CP and CR were associated with superior outcomes compared to CU in selected parameters, offering a more differentiated view of capsular management in contemporary HAS.

These findings have direct clinical relevance. Both CP and CR are preferable to CU based on functional outcomes. If adequate access is possible with periportal approaches, CP may reduce operative time and tissue trauma without compromising results. In patients with wider portals or borderline dysplasia, adding closure during CP may improve stability. When a formal capsulotomy is needed, CR offers advantages over leaving it open. Because complication rates were low across groups, the choice between CP and CR should be tailored to anatomy, biomechanics, and procedure‐specific factors rather than a universal rule. Surgeons should consider instability risk, ligamentous laxity and capsule size, as well as technical factors and experience, when deciding between preservation and repair.

Future trials should directly compare CP, CR and CU in randomized settings, with stratification by instability risk, laxity or hypermobility. A formal network meta‐analysis using Bayesian and frequentist methods could rank strategies across outcomes. Short‐, mid‐, and long‐term effects should be analyzed separately, as current evidence is mostly early and mid‐term. Post‐operative MRI could help assess capsule continuity and thickness as markers of biomechanical restoration. Standardizing outcomes, surgical definitions and follow‐up intervals is essential to reduce heterogeneity and allow reliable comparisons.

Strengths of this study include: (i) a comprehensive search across multiple databases without language or date limits, (ii) a large aggregated sample of 47 studies and >7000 hips, (iii) inclusion of all three strategies (CP, CR and CU), (iv) the use of a multilevel model accounting for variance within and between studies and (v) restriction to short‐ and mid‐term outcomes. Limitations include: (i) substantial heterogeneity across studies, from RCTs to case series with variable patients and techniques. High *I* ^2^ values in some outcomes reduce the strength of pooled estimates despite robust modelling. Results should therefore be interpreted with caution, and future RCTs with standardized outcomes are needed. (ii) Most studies did not distinguish between partial and non‐repair. In order to ensure methodological rigour, we have consistently categorized partial repair cases within the CU group. Strictly speaking, a partially repaired capsule is still unrepaired. We acknowledge that this approach may mask potential differences, and therefore emphasize that future trials should report partial repairs and non‐repairs separately. (iii) The evidence base is dominated by retrospective cohorts, (iv) direct randomized comparisons of all three strategies are lacking and (v) reporting bias and inconsistent classification of capsular techniques may have affected comparability.

## CONCLUSION

CR and CP were consistently associated with better outcomes than leaving the CU. Both strategies appear safe and effective, and may be considered standard in contemporary HAS. Surgeons should avoid leaving the capsule unrepaired, while future trials will refine indications for CP versus CR.

## AUTHOR CONTRIBUTIONS

Nikolai Ramadanov, Maximilian Voss and Maximilian Heinz performed the literature search, the data extraction and the risk of bias assessment. Robert Hable and Nikolai Ramadanov conducted the statistical calculations. Nikolai Ramadanov created all figures and tables. Nikolai Ramadanov wrote the manuscript. Ingo J. Banke, Robert Prill and Roland Becker supervised the work.

## CONFLICT OF INTEREST STATEMENT

The authors declare no conflicts of interest.

## ETHICS STATEMENT

The ethics statement is not available.

## Supporting information

Suppl Figure 1 Funnel Plot mHHS postop total.

Suppl Figure 2 Funnel Plot iHOT postop total.

Suppl Figure 3 Funnel Plot HOS ADL postop total.

Suppl Figure 4 Funnel Plot HOS SSS postop total.

Suppl Figure 5 Funnel Plot NAHS postop total.

Suppl Figure 6 Funnel Plot VAS postop total.

Suppl Figure 7 Funnel Plot Change in mHHS.

Suppl Figure 8 Funnel Plot Change in iHOT.

Suppl Figure 9 Funnel Plot Change in HOS ADL.

Suppl Figure 10 Funnel Plot Change in HOS SSS.

Suppl Figure 11 Funnel Plot Change in NAHS.

Suppl Figure 12 Funnel Plot Change in VAS.

Suppl Figure 13 Funnel Plot MCID Function after 3 months.

Suppl Figure 14 Funnel Plot MCID Function after 6 months.

Suppl Figure 15 Funnel Plot MCID Function after 12 months.

Suppl Figure 16 Funnel Plot MCID Function after 24 months.

Suppl Figure 17 Funnel Plot MCID Pain last follow‐up.

Suppl Figure 18 Funnel Plot Overall complications.

Suppl Figure 19 Funnel Plot DVTPE.

Suppl Figure 20 Funnel Plot Nerve injury.

Suppl Figure 21 Funnel Plot Infection.

Suppl Figure 22 Funnel Plot Haematoma.

Suppl Figure 23 Funnel Plot Revision.

Suppl Figure 24 Funnel Plot Converted to THA.

Suppl Figure 25 Forestplot_mHHS postoperative total.

Suppl Figure 26 Forestplot_iHOT postoperative total.

Suppl Figure 27 Forestplot_HOS ADL postoperative total.

Suppl Figure 28 Forestplot_HOS SSS postoperative total.

Suppl Figure 29 Forestplot_NAHS postoperative total.

Suppl Figure 30 Forestplot_VAS postoperative total.

Suppl Figure 31 Forestplot_Change in iHOT.

Suppl Figure 32 Forestplot_Change in HOS ADL.

Suppl Figure 33 Forestplot_Change in HOS SSS.

Suppl Figure 34 Forestplot_Change in NAHS.

Suppl Figure 35 Forestplot_Change in VAS.

Suppl Figure 36 Forestplot_MCID Function after 3 months.

Suppl Figure 37 Forestplot_MCID Function after 6 months.

Suppl Figure 38 Forestplot_MCID Function after 24 months.

Suppl Figure 39 Forestplot_MCID Pain last follow up.

Suppl Figure 40 Forestplot_Overall complications.

Suppl Figure 41 Forestplot_DVT‐PE.

Suppl Figure 42 Forestplot_Nerve injury.

Suppl Figure 43 Forestplot_Infection.

Suppl Figure 44 Forestplot_Haematoma.

Suppl Figure 45 Forestplot_Revision.

Suppl Figure 46 Forestplot_Converted to THA.

Suppl Figure 47 Forestplot_mHHS preoperative.

Suppl Figure 48 Forestplot_iHOT preoperative.

Suppl Figure 49 Forestplot_HOS ADL preoperative.

Suppl Figure 50 Forestplot_HOS Sport preoperative.

Suppl Figure 51 Forestplot_NAHS preoperative.

Suppl Figure 52 Forestplot_VAS preoperative.

Suppl Figure 53 Forestplot_mHHS after 2‐12 months.

Suppl Figure 54 Forestplot_mHHS after 24 months.

Suppl Figure 55 Forestplot_HOS ADL after 3‐12 months.

Suppl Figure 56 Forestplot_HOS ADL after 24 months.

Suppl Figure 57 Forestplot_HOS Sport after 3 months.

Suppl Figure 58 Forestplot_HOS Sport after 6 months.

Suppl Figure 59 Forestplot_HOS Sport after 12 months.

Suppl Figure 60 Forestplot_HOS Sport after 24 months.

Suppl Figure 61 Forestplot_HOOS ADL after 6‐24 months.

Suppl Figure 62 Forestplot_HOOS Pain after 6‐24 months.

Suppl Figure 63 Forestplot_HOOS QoL after 6‐24 months.

Suppl Figure 64 Forestplot_HOOS Sport after 3‐24 months.

Suppl Figure 65 Forestplot_HOOS Symptoms after 6‐24 months.

Suppl Figure 66 Forestplot_HAGOS ADL after 3‐24 months.

Suppl Figure 67 Forestplot_HAGOS Pain after 3‐24 months.

Suppl Figure 68 Forestplot_HAGOS QoL after 3‐24 months.

Suppl Figure 69 Forestplot_HAGOS Sport after 3‐24 months.

Suppl Figure 70 Forestplot_HAGOS Symptoms after 3‐24 months.

Suppl Figure 71 Forestplot_VAS after 7 days ‐ 3 months.

Suppl Figure 72 Forestplot_VAS after 6‐12 months.

Suppl Figure 73 Forestplot_VAS after 24 months.

Suppl Table 1 PRISMA 2020 checklist.

Suppl Table 2 Preoperative.

Suppl Table 3 Additional outcomes.

Supmat.

## Data Availability

Available from the corresponding author upon reasonable request.
