# Supplementary figures and images for: Capsular management strategies in hip arthroscopy for femoroacetabular impingement syndrome: A multilevel meta‐analysis
Source: Knee Surg Sports Traumatol Arthrosc. 2025 Oct 17;34(1):284–308. doi: 10.1002/ksa.70094 (PMC12747625; doi:10.1002/ksa.70094)

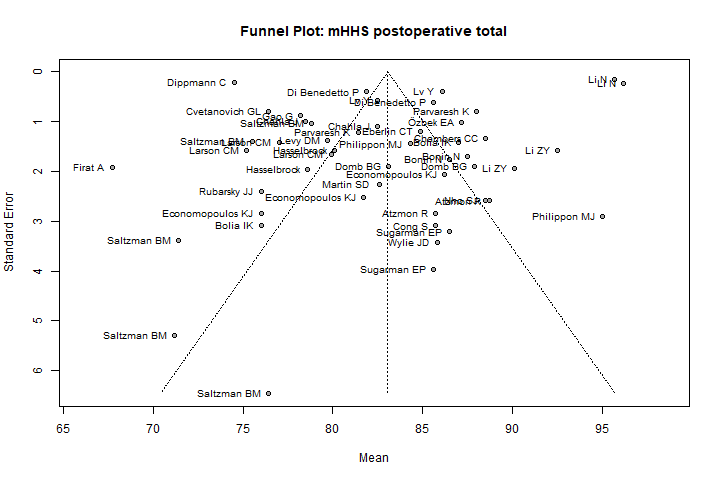

Supplement: Supplementary file 1 — Suppl Figure 1 Funnel Plot mHHS postop total. [file KSA-34-284-s072.png]

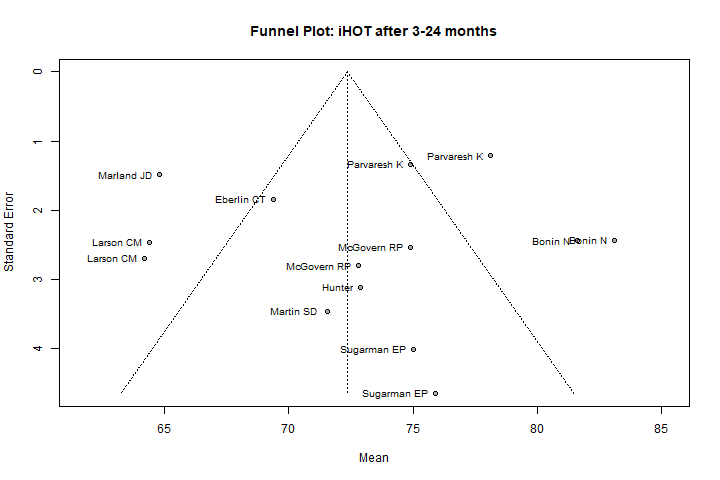

Supplement: Supplementary file 2 — Suppl Figure 2 Funnel Plot iHOT postop total. [file KSA-34-284-s057.png]

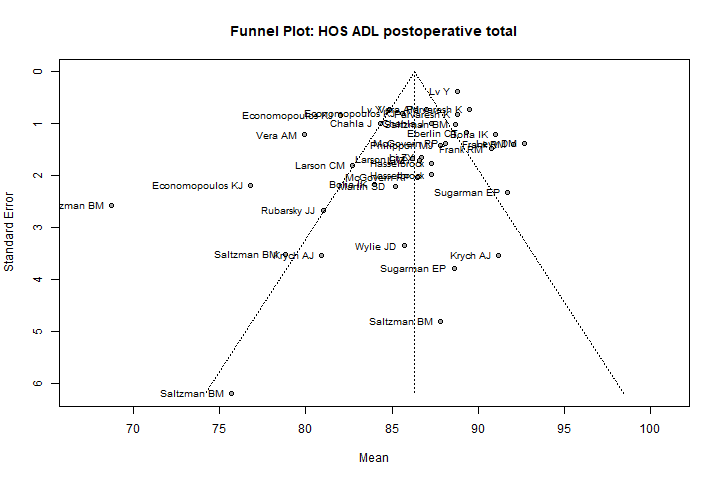

Supplement: Supplementary file 3 — Suppl Figure 3 Funnel Plot HOS ADL postop total. [file KSA-34-284-s060.png]

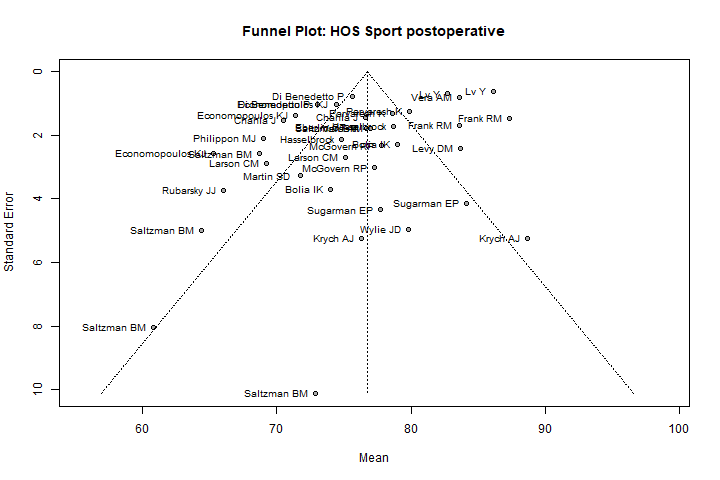

Supplement: Supplementary file 4 — Suppl Figure 4 Funnel Plot HOS SSS postop total. [file KSA-34-284-s043.png]

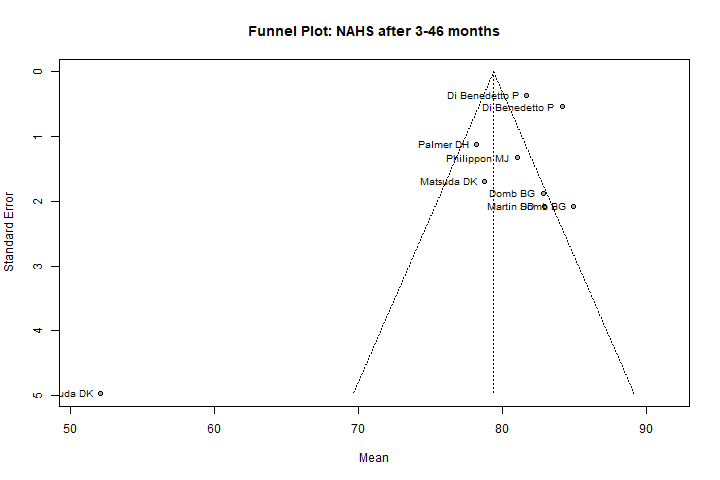

Supplement: Supplementary file 5 — Suppl Figure 5 Funnel Plot NAHS postop total. [file KSA-34-284-s007.png]

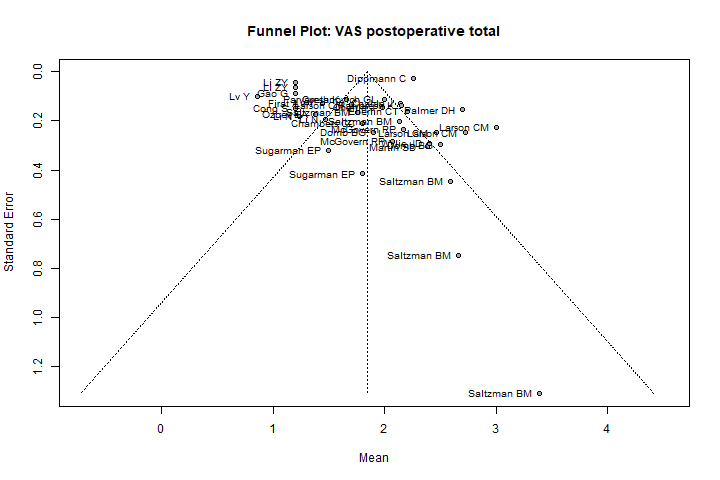

Supplement: Supplementary file 6 — Suppl Figure 6 Funnel Plot VAS postop total. [file KSA-34-284-s005.png]

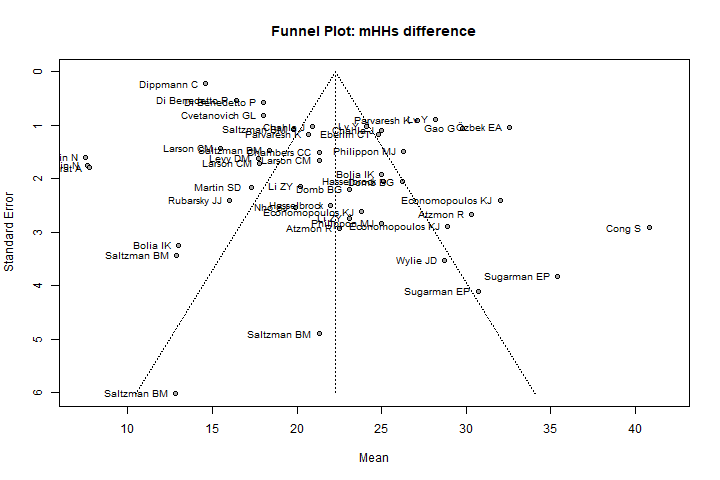

Supplement: Supplementary file 7 — Suppl Figure 7 Funnel Plot Change in mHHS. [file KSA-34-284-s053.png]

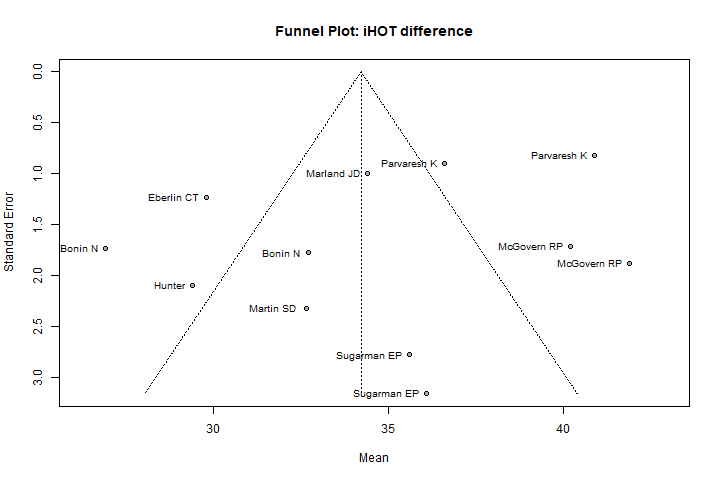

Supplement: Supplementary file 8 — Suppl Figure 8 Funnel Plot Change in iHOT. [file KSA-34-284-s039.png]

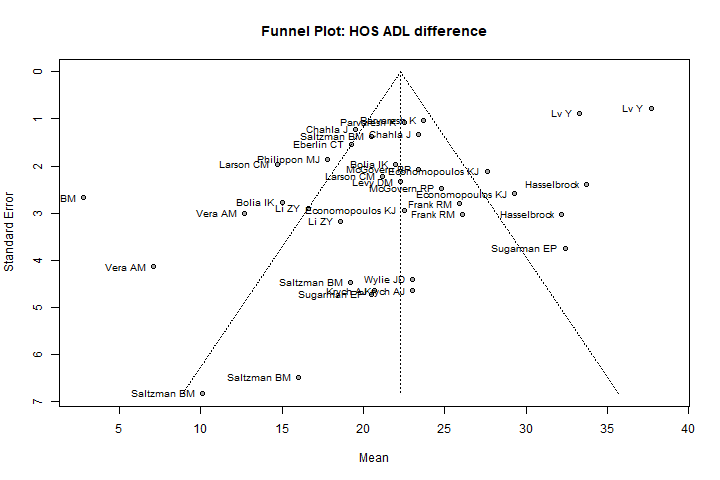

Supplement: Supplementary file 9 — Suppl Figure 9 Funnel Plot Change in HOS ADL. [file KSA-34-284-s075.png]

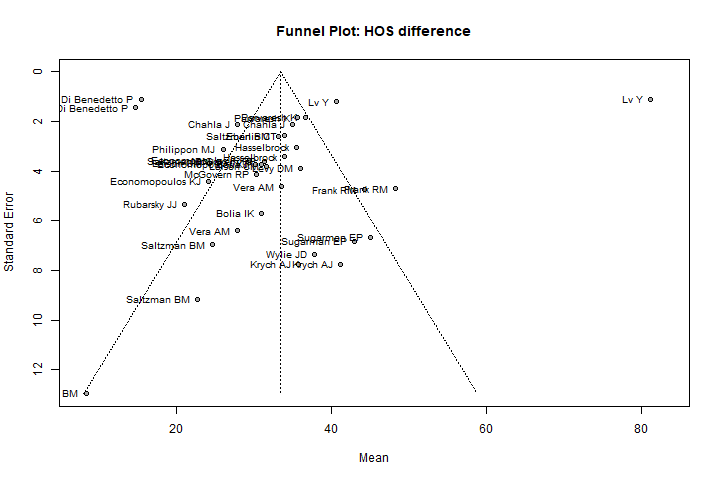

Supplement: Supplementary file 10 — Suppl Figure 10 Funnel Plot Change in HOS SSS. [file KSA-34-284-s020.png]

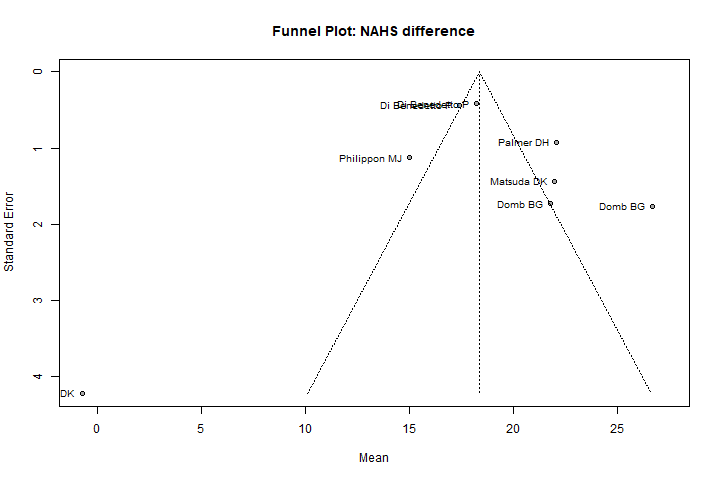

Supplement: Supplementary file 11 — Suppl Figure 11 Funnel Plot Change in NAHS. [file KSA-34-284-s025.png]

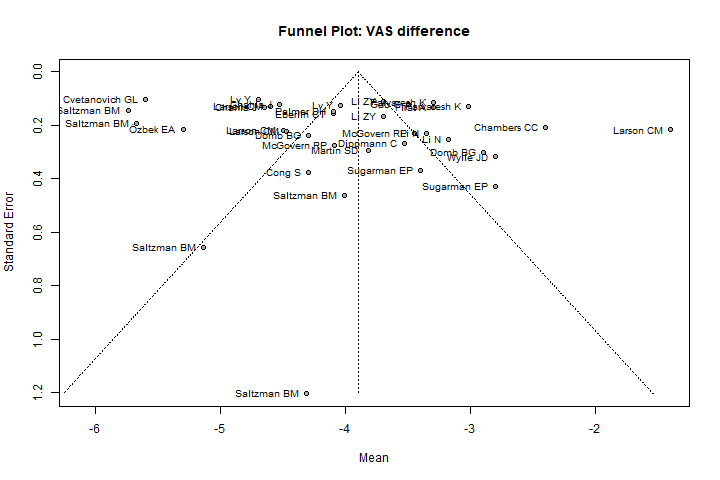

Supplement: Supplementary file 12 — Suppl Figure 12 Funnel Plot Change in VAS. [file KSA-34-284-s051.png]

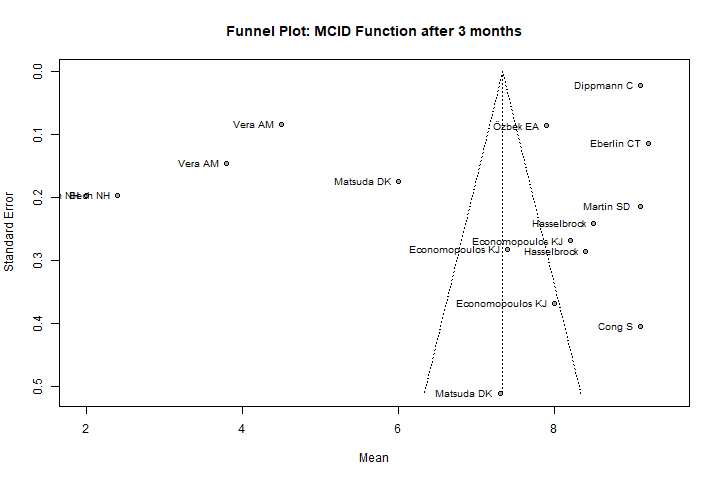

Supplement: Supplementary file 13 — Suppl Figure 13 Funnel Plot MCID Function after 3 months. [file KSA-34-284-s068.png]

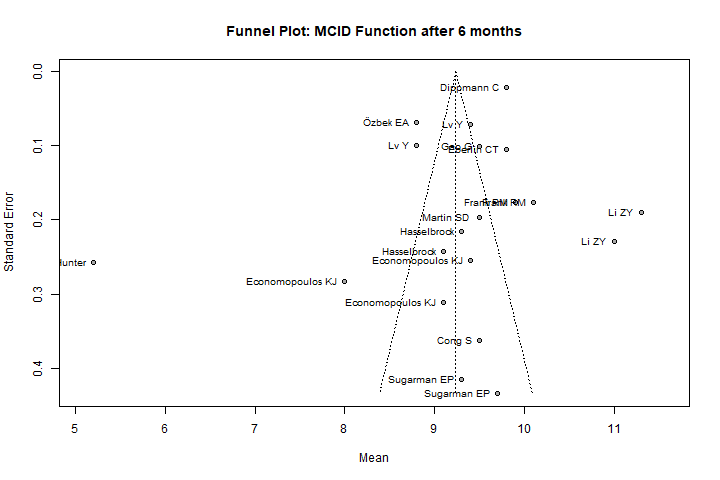

Supplement: Supplementary file 14 — Suppl Figure 14 Funnel Plot MCID Function after 6 months. [file KSA-34-284-s009.png]

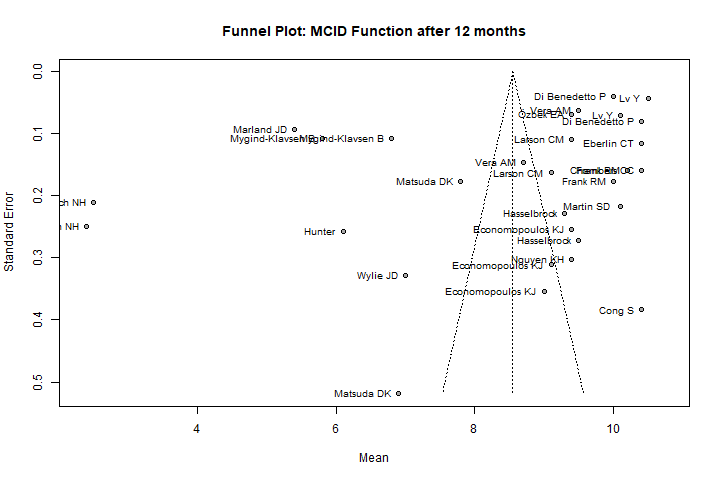

Supplement: Supplementary file 15 — Suppl Figure 15 Funnel Plot MCID Function after 12 months. [file KSA-34-284-s045.png]

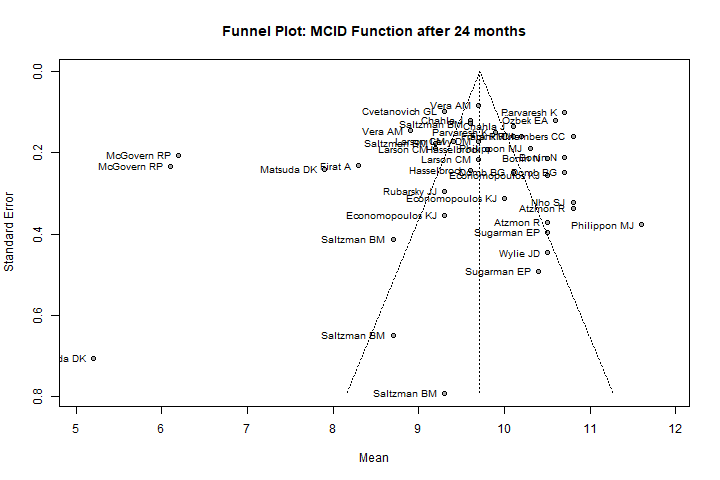

Supplement: Supplementary file 16 — Suppl Figure 16 Funnel Plot MCID Function after 24 months. [file KSA-34-284-s001.png]

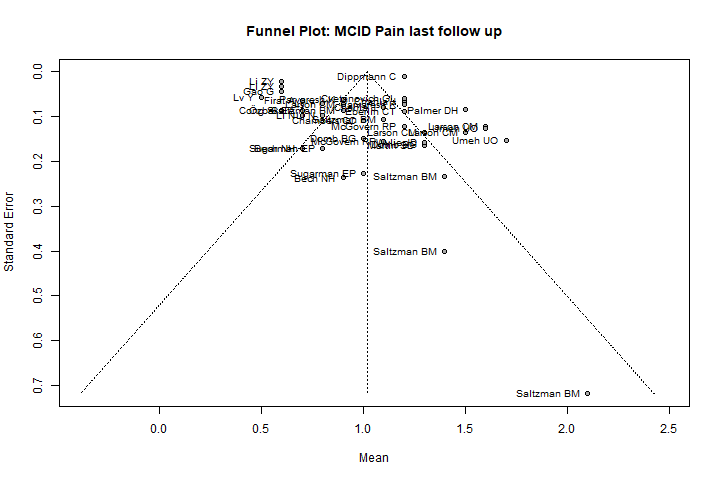

Supplement: Supplementary file 17 — Suppl Figure 17 Funnel Plot MCID Pain last follow‐up. [file KSA-34-284-s076.png]

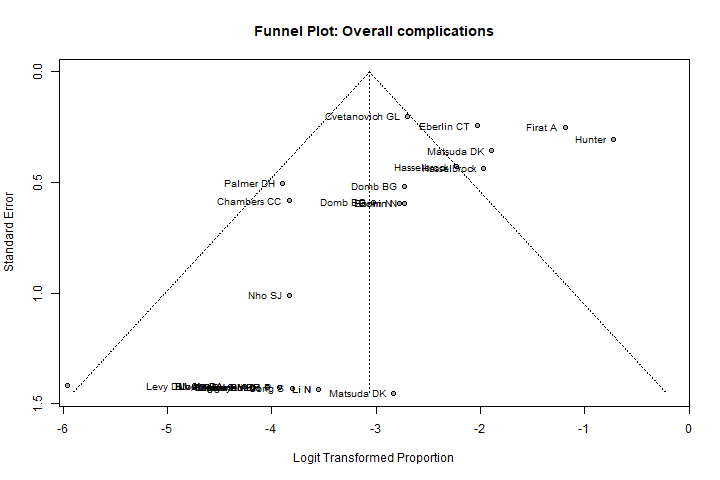

Supplement: Supplementary file 18 — Suppl Figure 18 Funnel Plot Overall complications. [file KSA-34-284-s038.png]

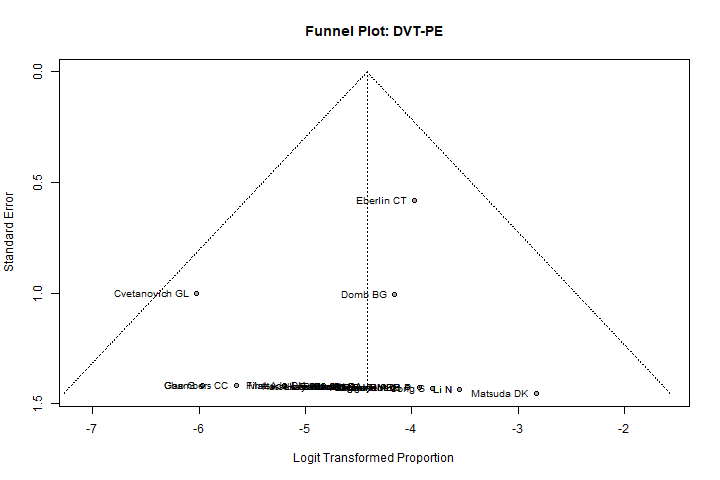

Supplement: Supplementary file 19 — Suppl Figure 19 Funnel Plot DVTPE. [file KSA-34-284-s059.png]

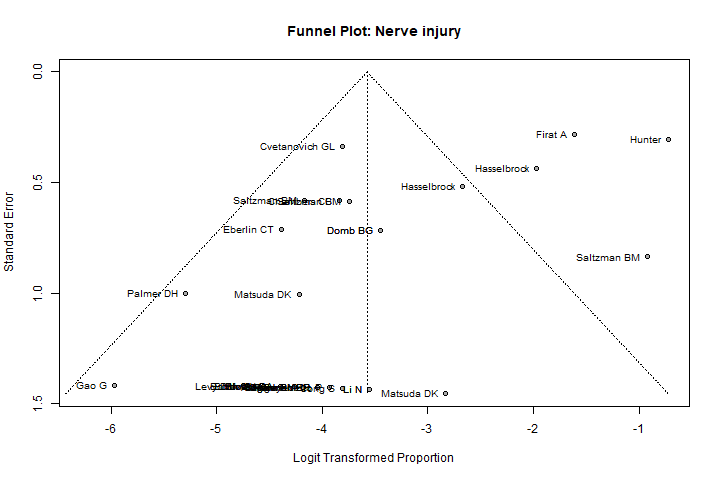

Supplement: Supplementary file 20 — Suppl Figure 20 Funnel Plot Nerve injury. [file KSA-34-284-s064.png]

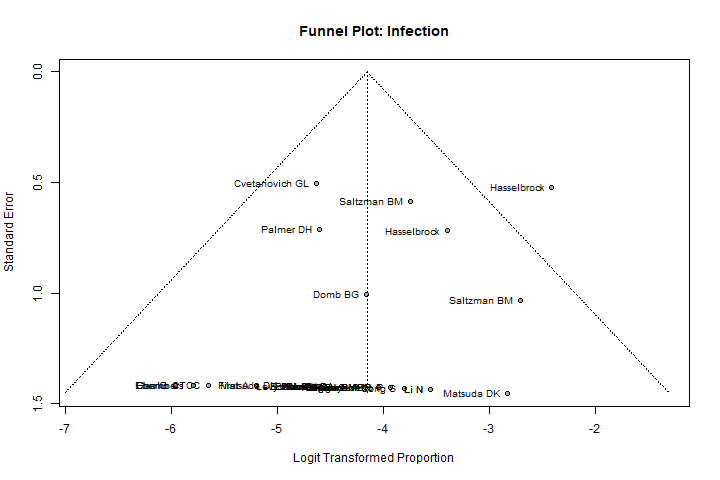

Supplement: Supplementary file 21 — Suppl Figure 21 Funnel Plot Infection. [file KSA-34-284-s066.png]

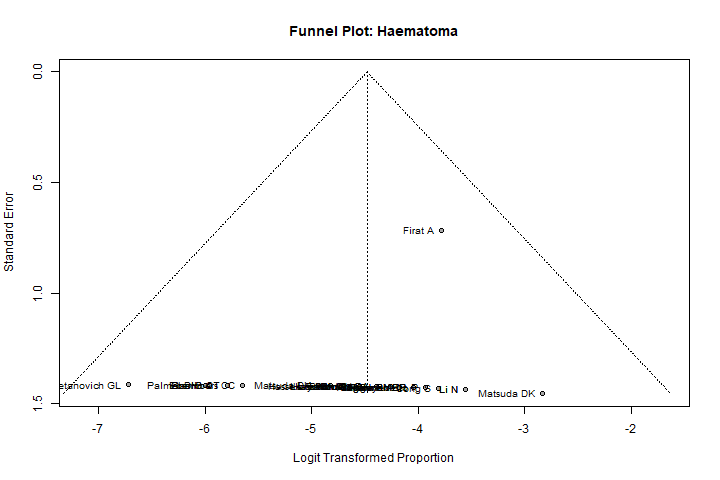

Supplement: Supplementary file 22 — Suppl Figure 22 Funnel Plot Haematoma. [file KSA-34-284-s067.png]

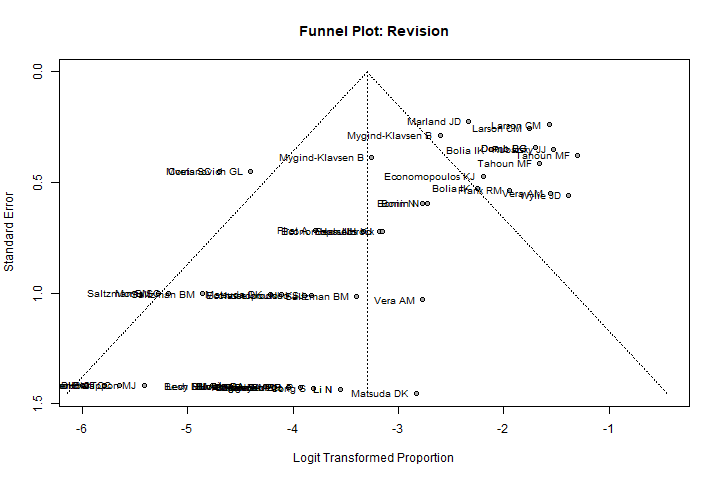

Supplement: Supplementary file 23 — Suppl Figure 23 Funnel Plot Revision. [file KSA-34-284-s029.png]

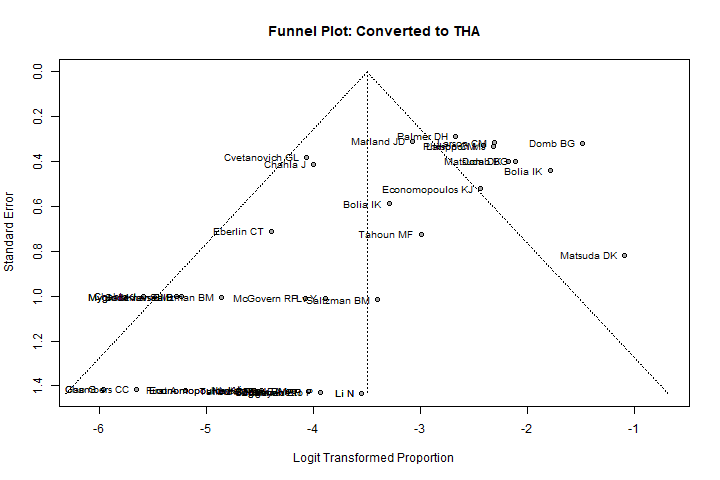

Supplement: Supplementary file 24 — Suppl Figure 24 Funnel Plot Converted to THA. [file KSA-34-284-s041.png]

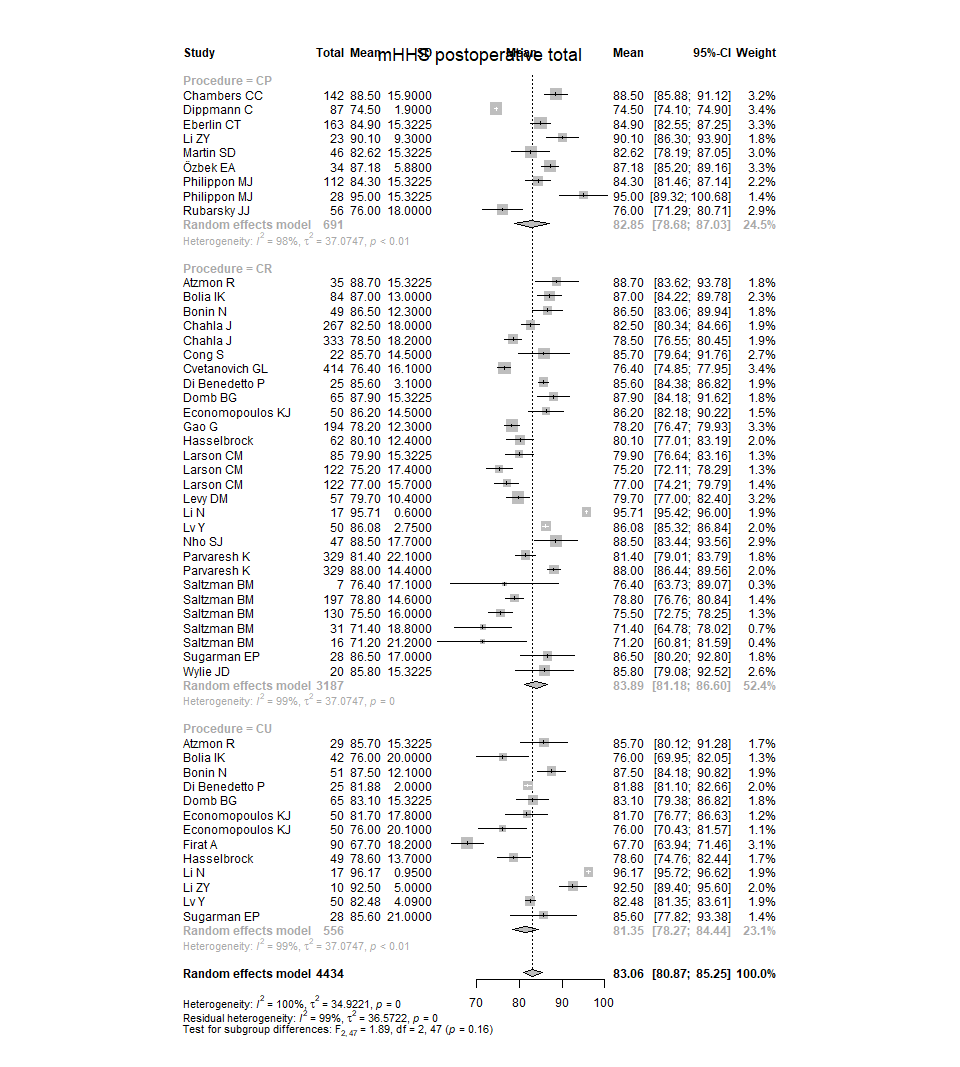

Supplement: Supplementary file 25 — Suppl Figure 25 Forestplot_mHHS postoperative total. [file KSA-34-284-s073.png]

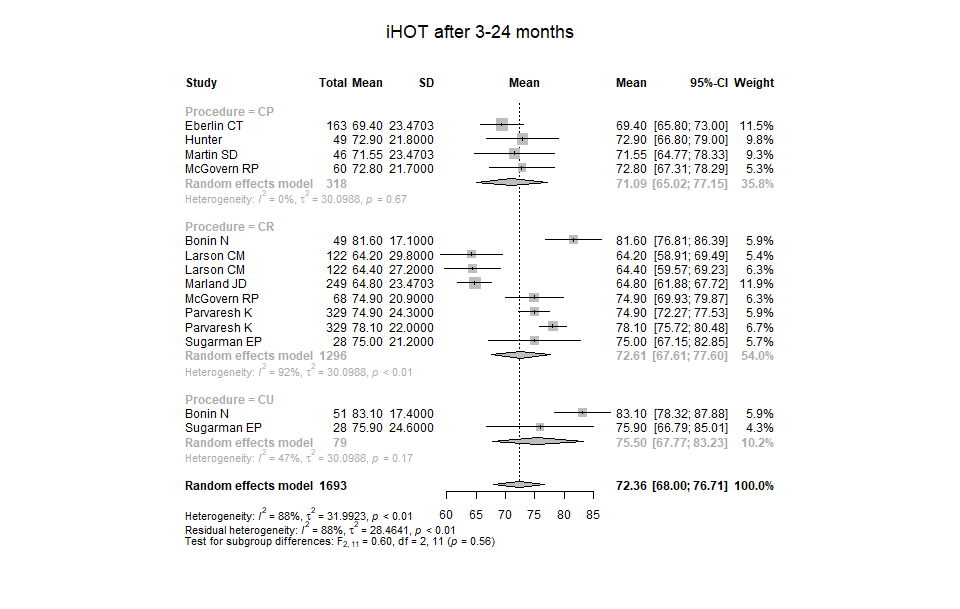

Supplement: Supplementary file 26 — Suppl Figure 26 Forestplot_iHOT postoperative total. [file KSA-34-284-s003.png]

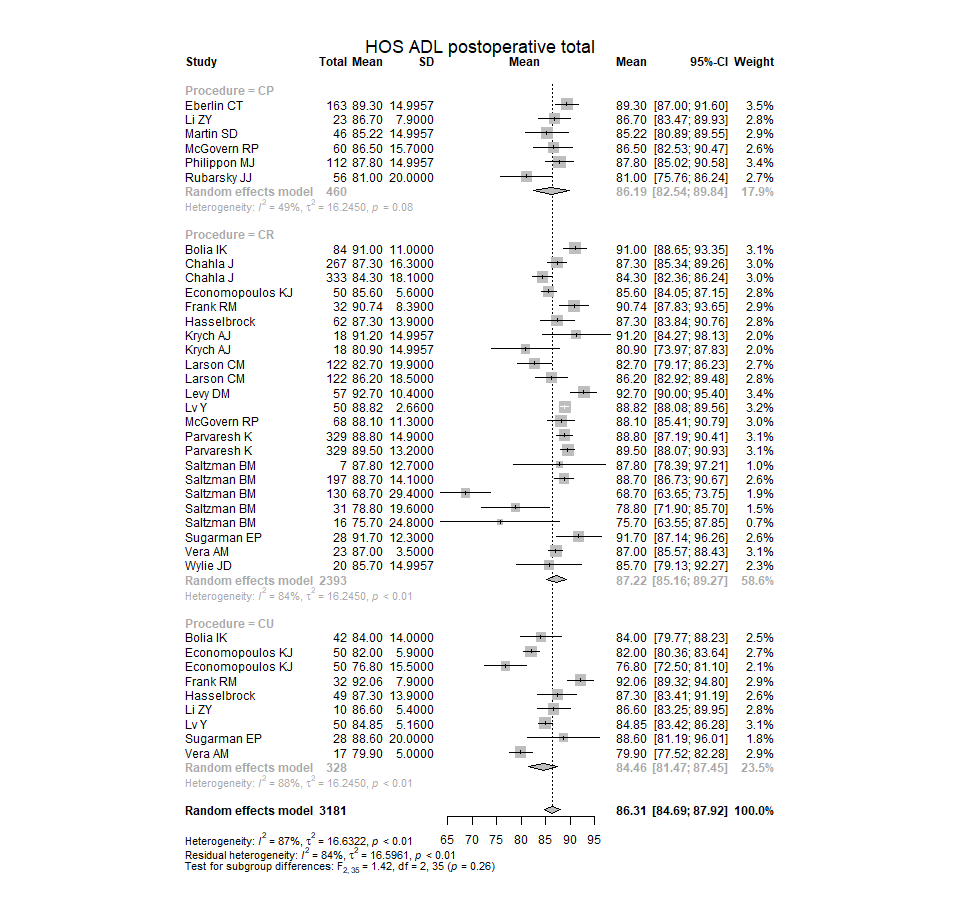

Supplement: Supplementary file 27 — Suppl Figure 27 Forestplot_HOS ADL postoperative total. [file KSA-34-284-s017.png]

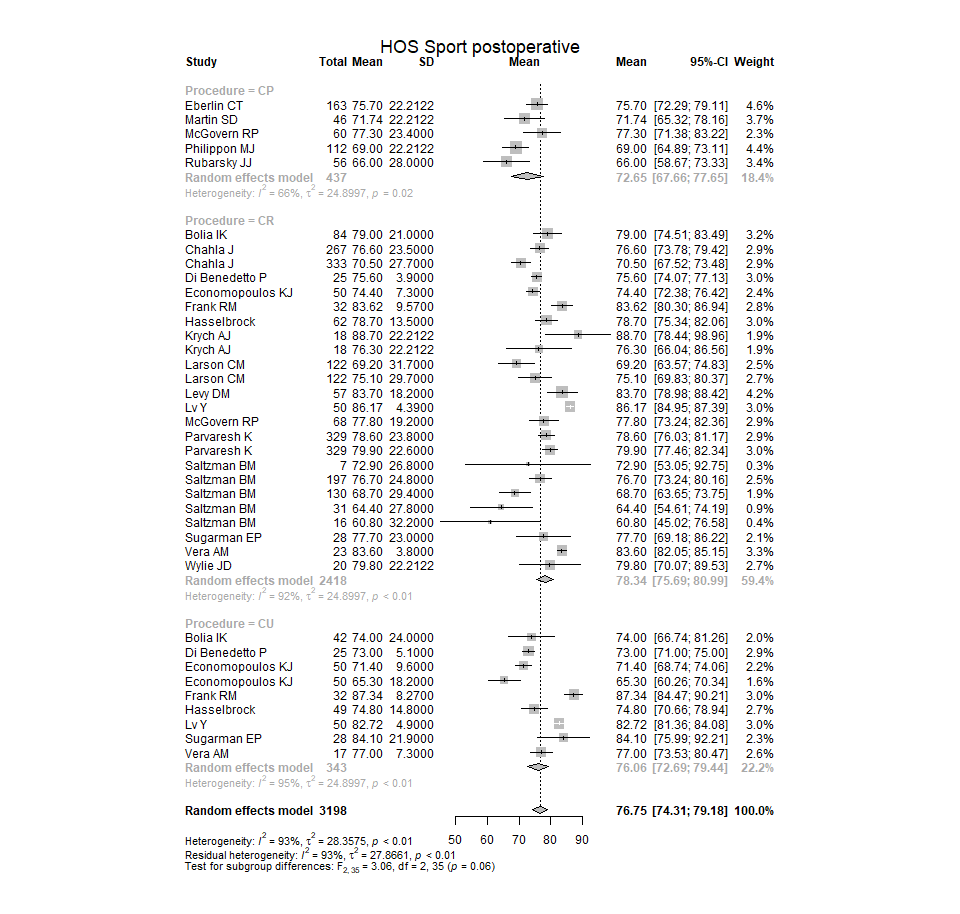

Supplement: Supplementary file 28 — Suppl Figure 28 Forestplot_HOS SSS postoperative total. [file KSA-34-284-s002.png]

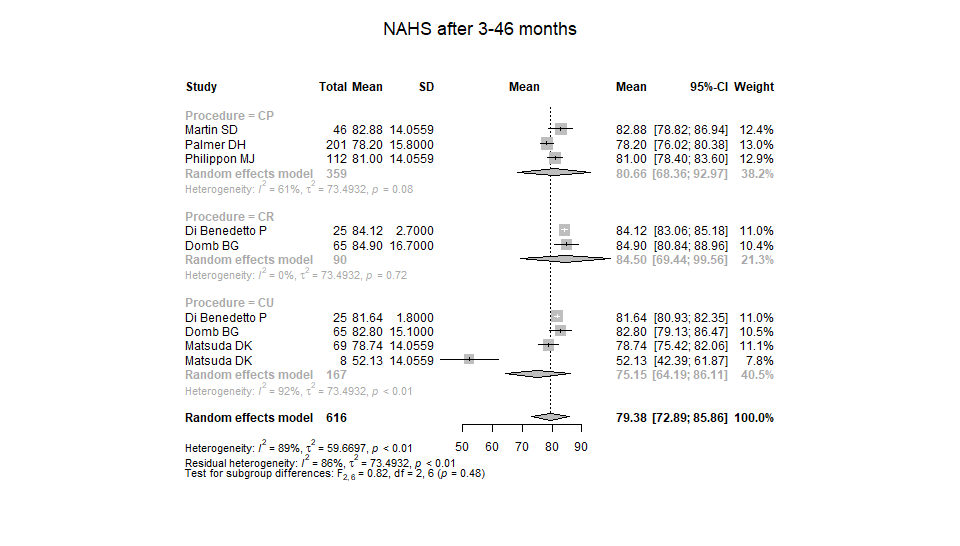

Supplement: Supplementary file 29 — Suppl Figure 29 Forestplot_NAHS postoperative total. [file KSA-34-284-s037.png]

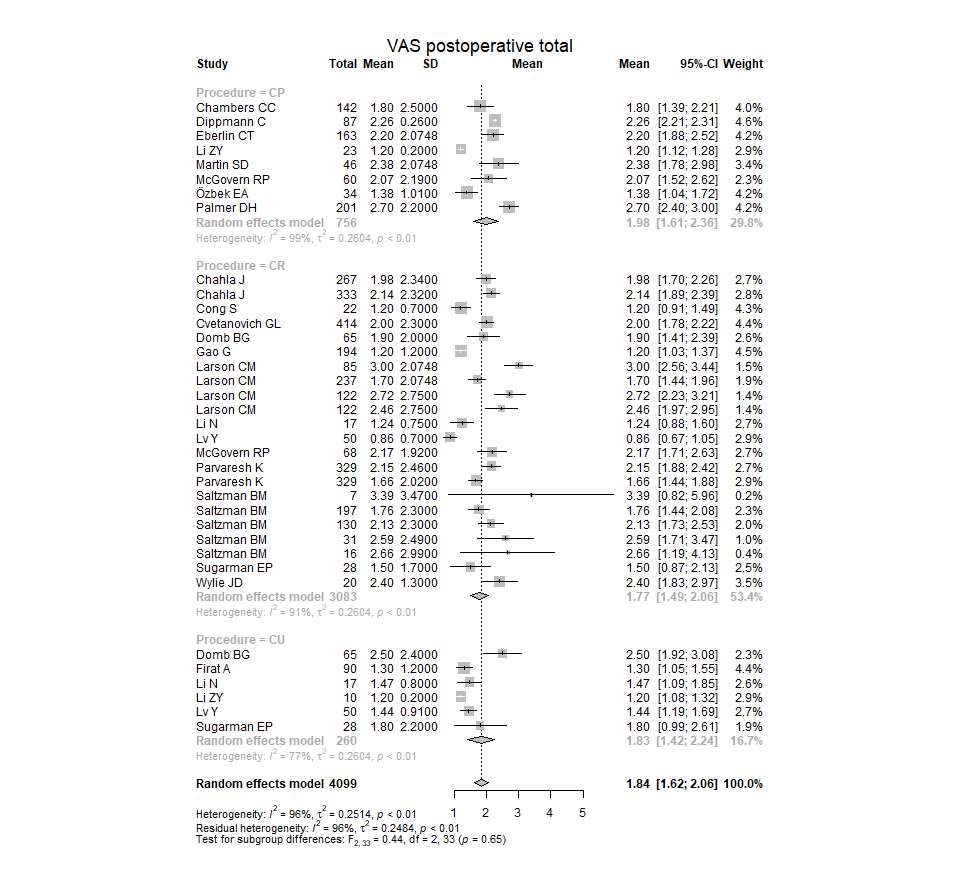

Supplement: Supplementary file 30 — Suppl Figure 30 Forestplot_VAS postoperative total. [file KSA-34-284-s013.png]

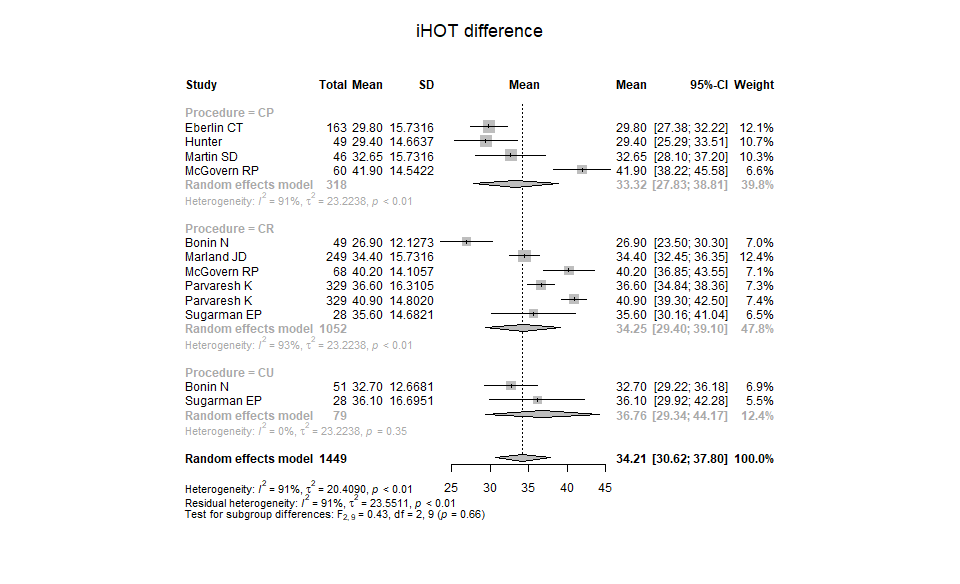

Supplement: Supplementary file 31 — Suppl Figure 31 Forestplot_Change in iHOT. [file KSA-34-284-s004.png]

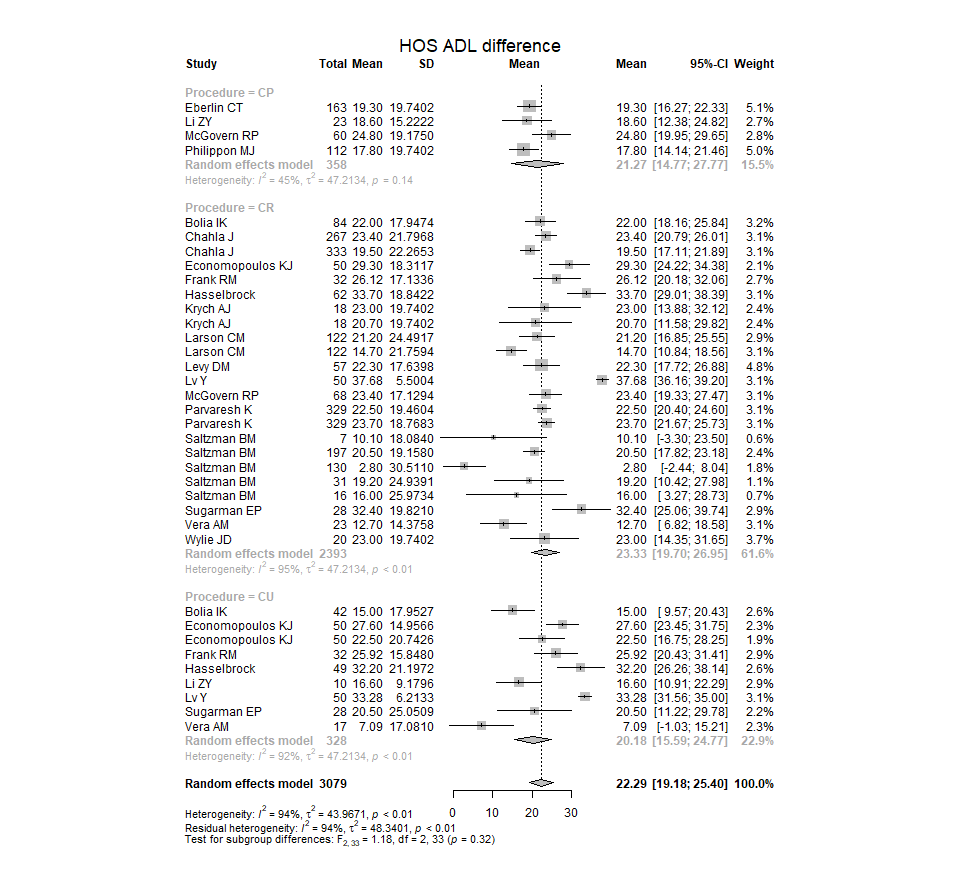

Supplement: Supplementary file 32 — Suppl Figure 32 Forestplot_Change in HOS ADL. [file KSA-34-284-s049.png]

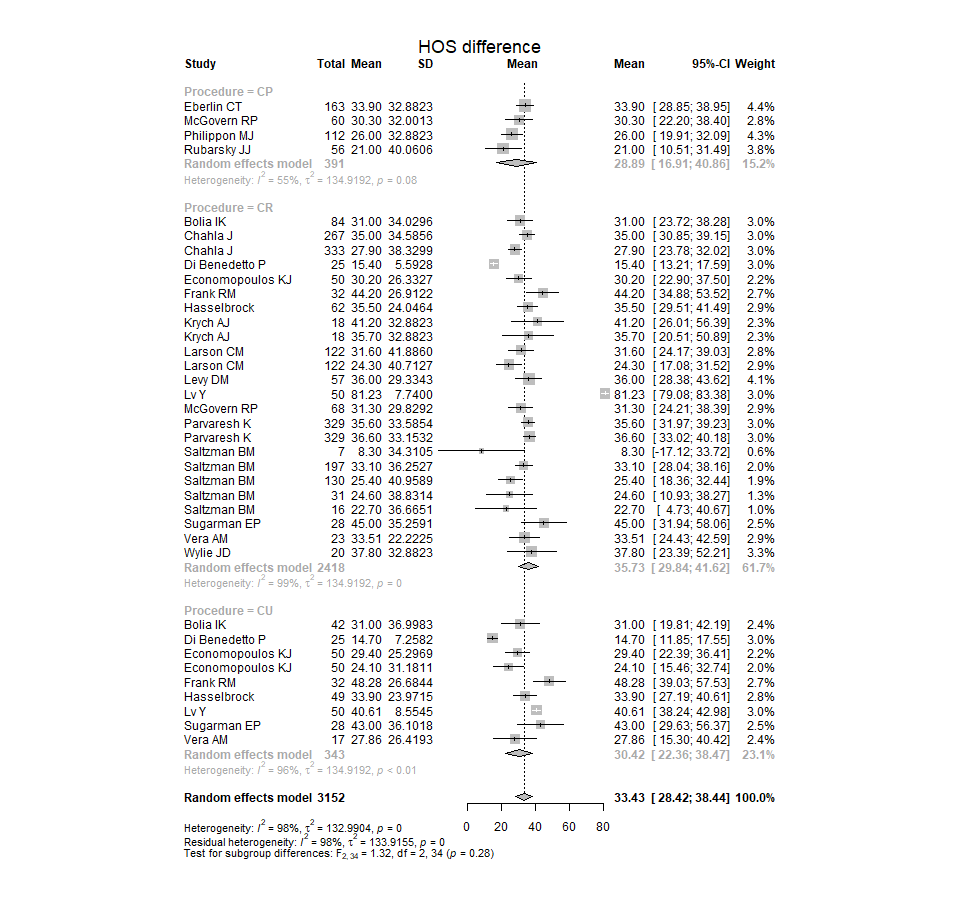

Supplement: Supplementary file 33 — Suppl Figure 33 Forestplot_Change in HOS SSS. [file KSA-34-284-s028.png]

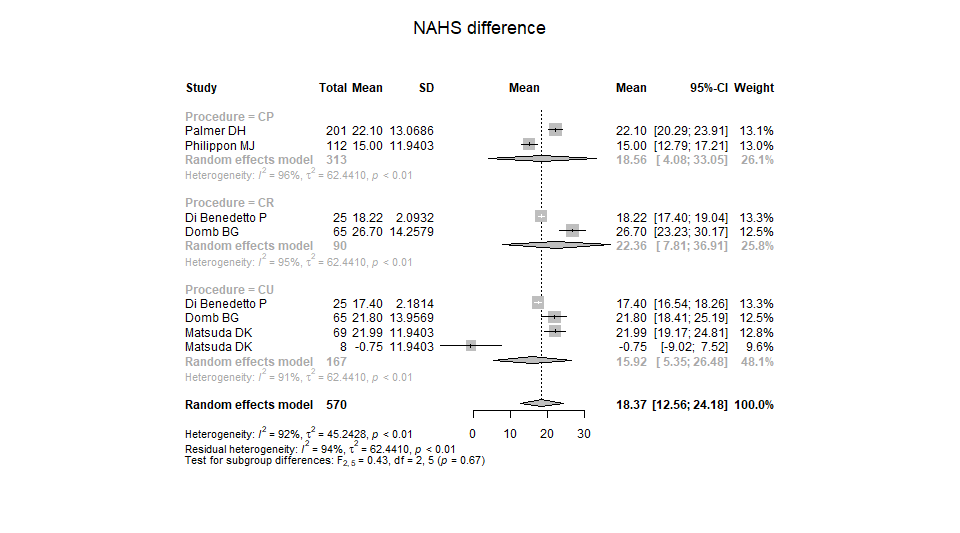

Supplement: Supplementary file 34 — Suppl Figure 34 Forestplot_Change in NAHS. [file KSA-34-284-s052.png]

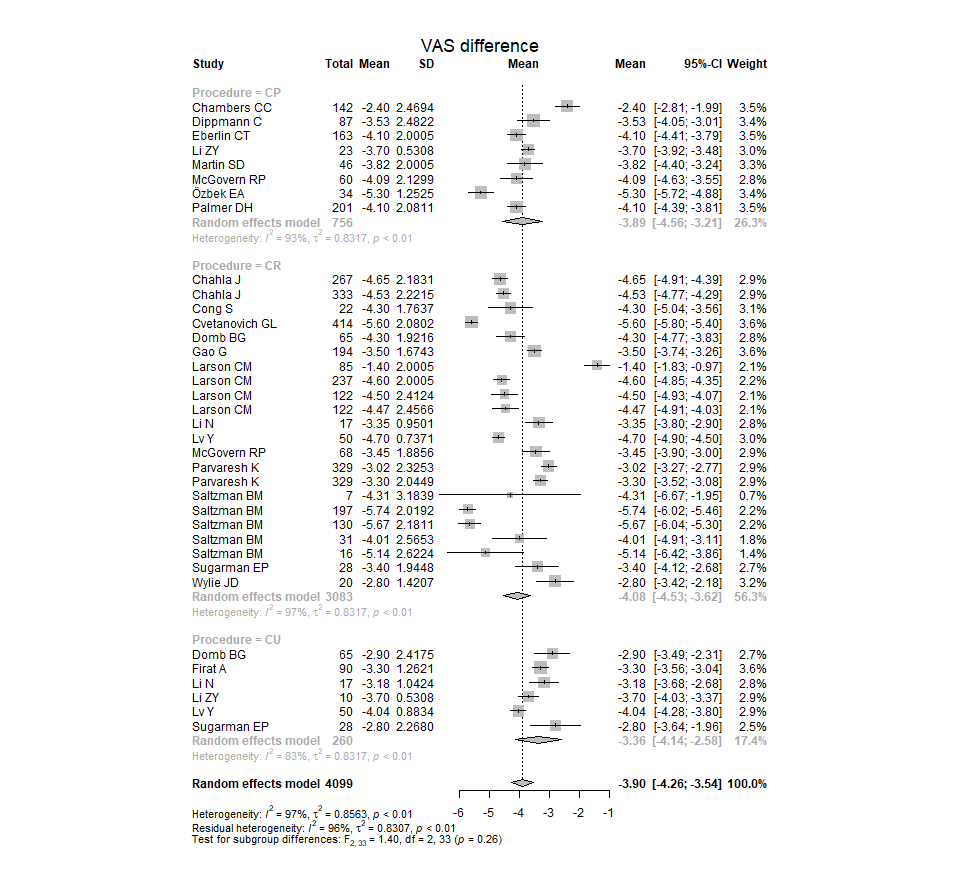

Supplement: Supplementary file 35 — Suppl Figure 35 Forestplot_Change in VAS. [file KSA-34-284-s022.png]

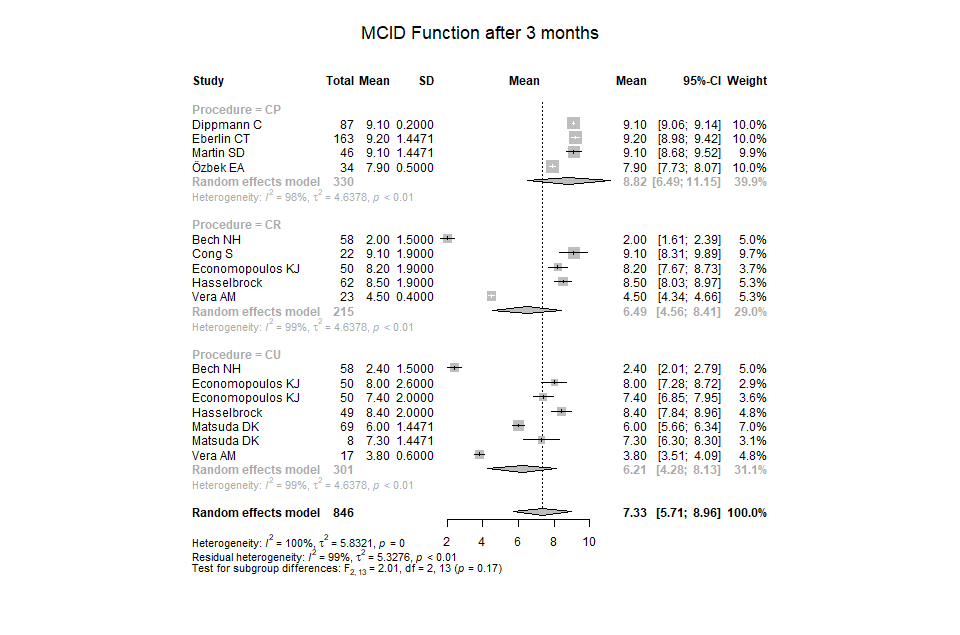

Supplement: Supplementary file 36 — Suppl Figure 36 Forestplot_MCID Function after 3 months. [file KSA-34-284-s030.png]

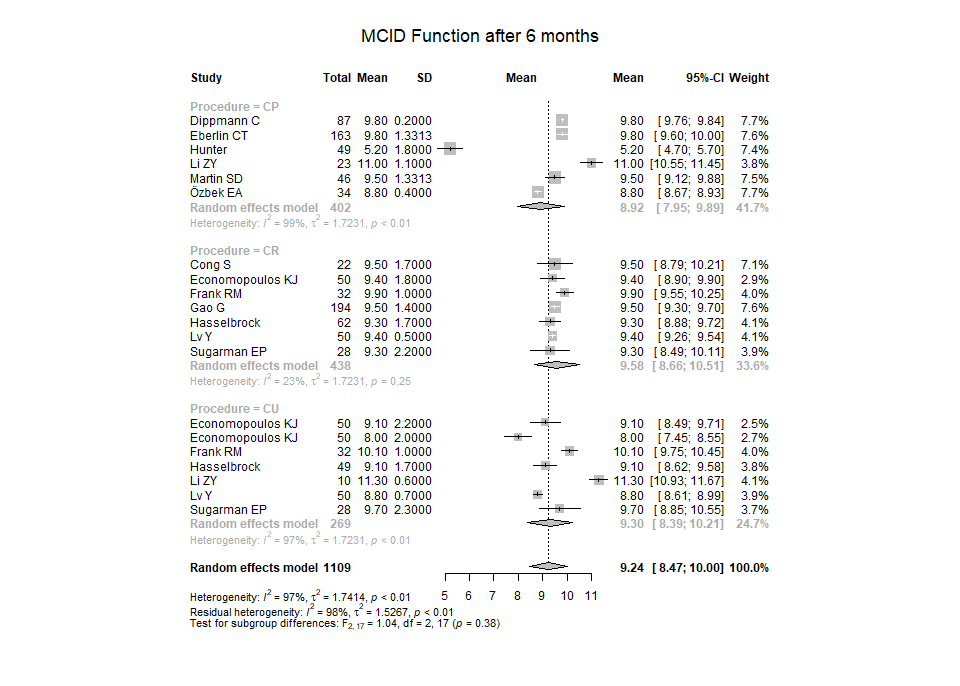

Supplement: Supplementary file 37 — Suppl Figure 37 Forestplot_MCID Function after 6 months. [file KSA-34-284-s036.png]

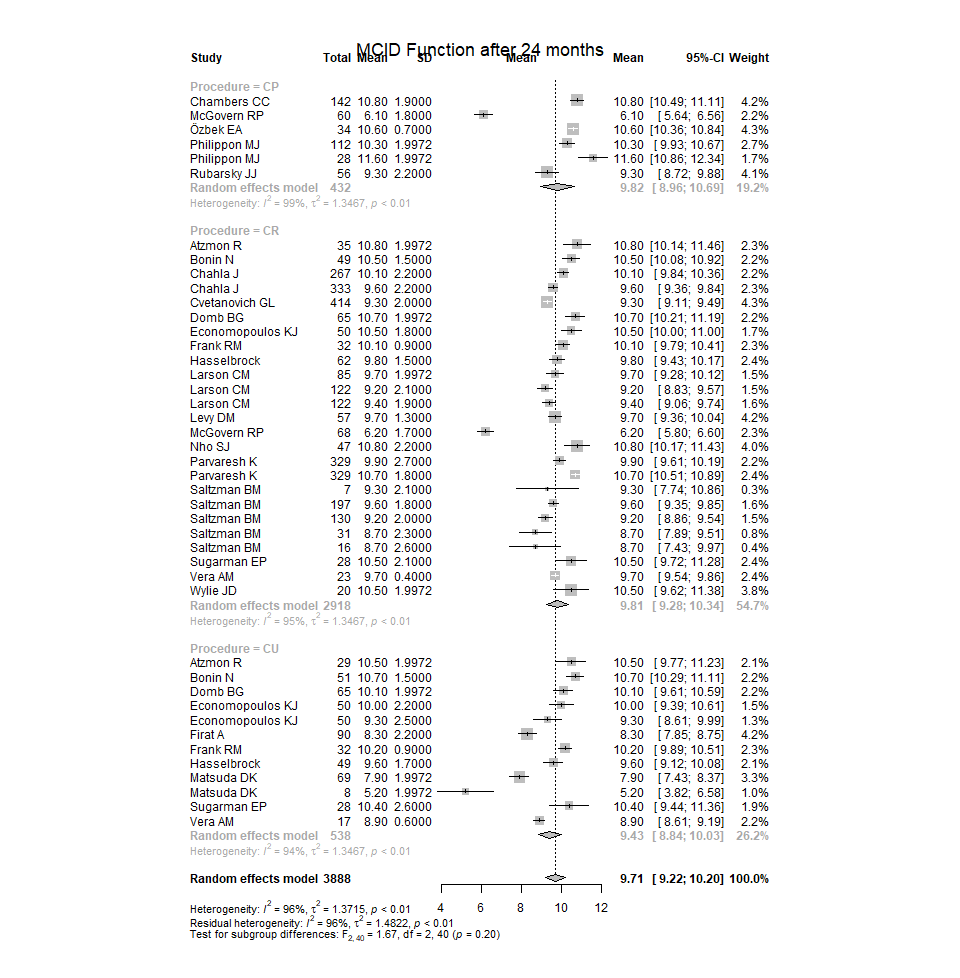

Supplement: Supplementary file 38 — Suppl Figure 38 Forestplot_MCID Function after 24 months. [file KSA-34-284-s033.png]

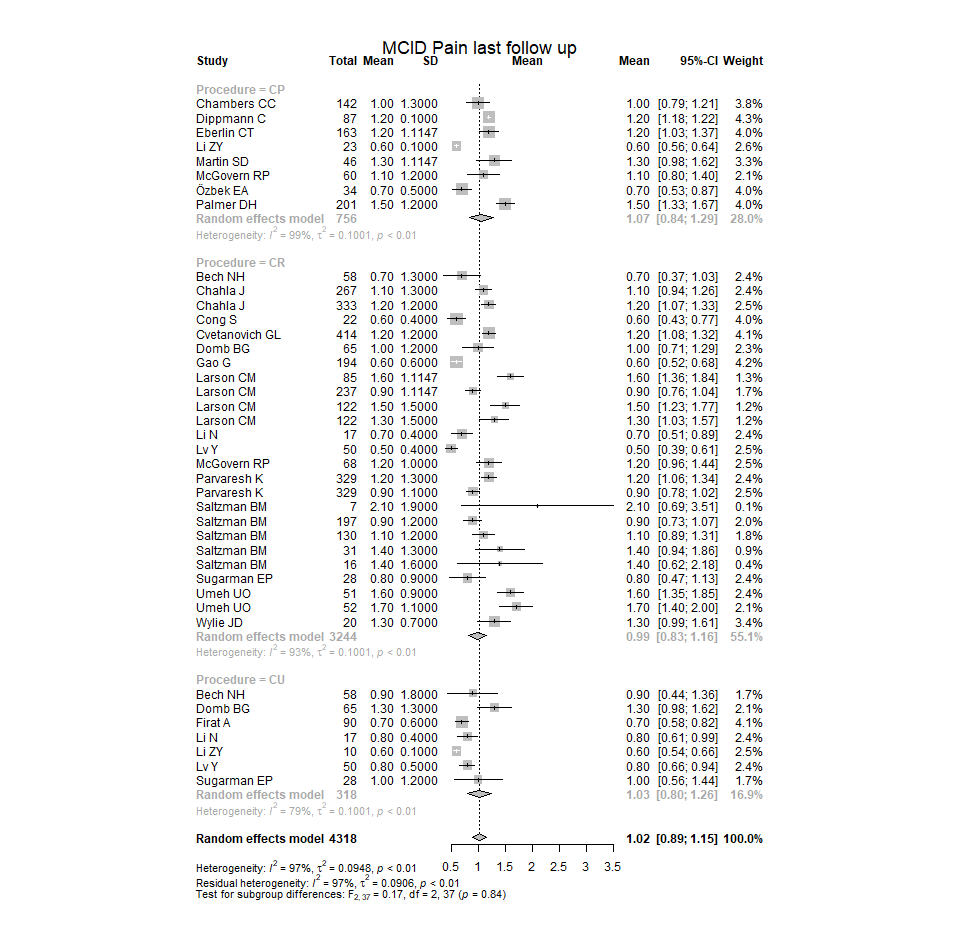

Supplement: Supplementary file 39 — Suppl Figure 39 Forestplot_MCID Pain last follow up. [file KSA-34-284-s019.png]

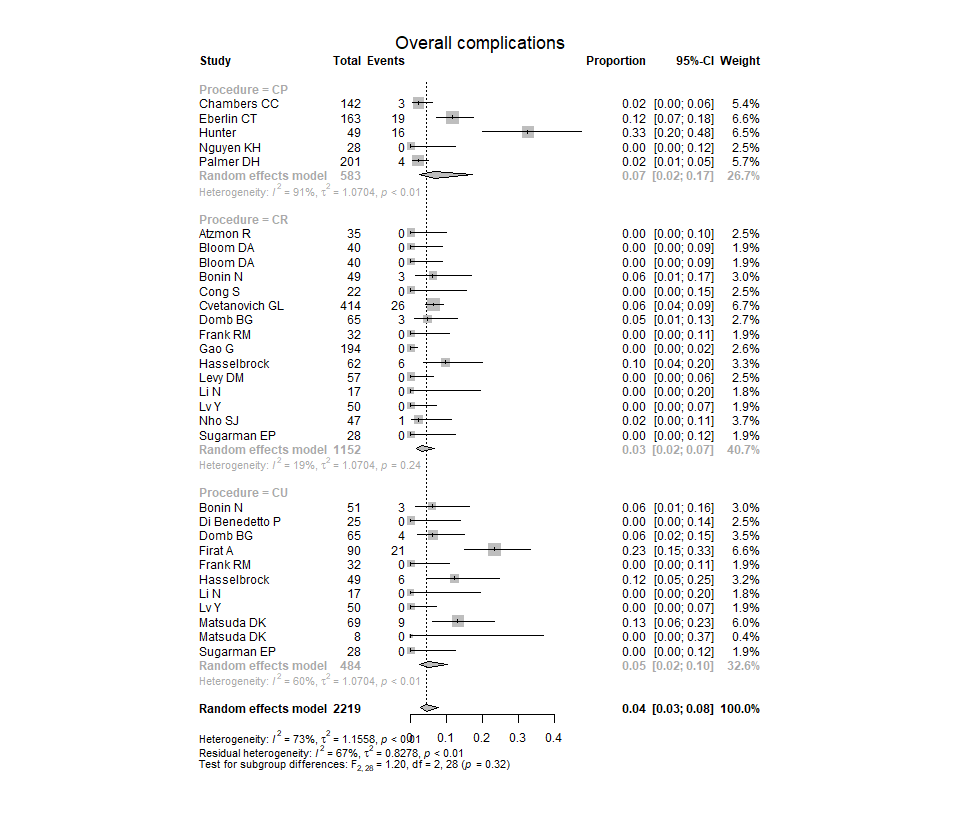

Supplement: Supplementary file 40 — Suppl Figure 40 Forestplot_Overall complications. [file KSA-34-284-s035.png]

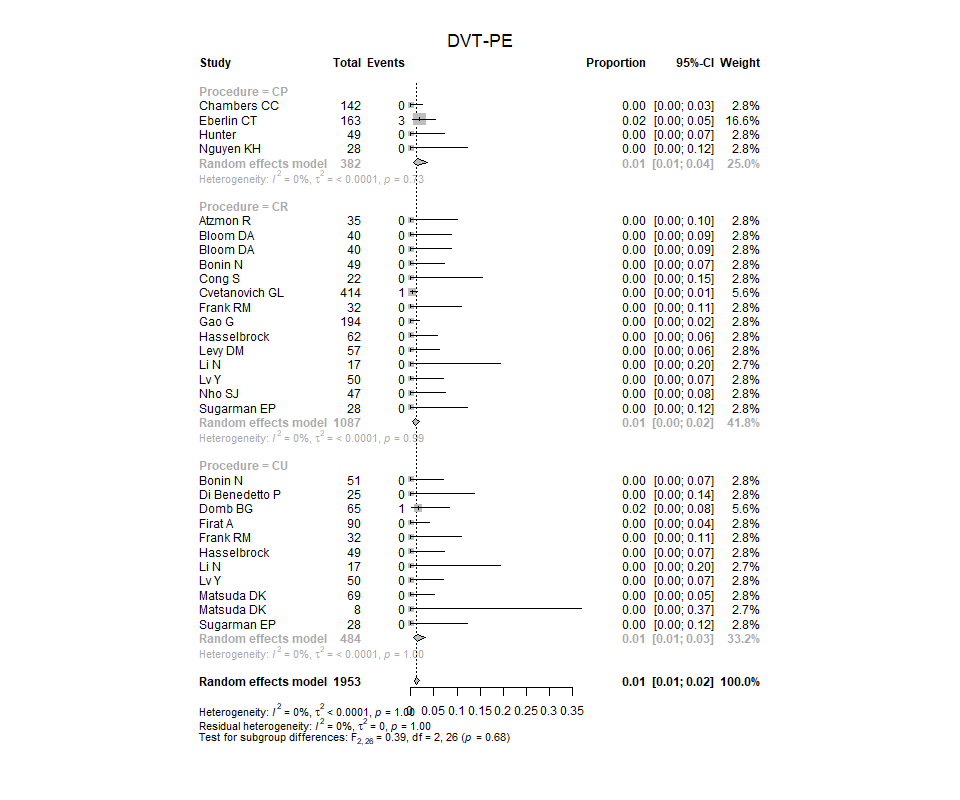

Supplement: Supplementary file 41 — Suppl Figure 41 Forestplot_DVT‐PE. [file KSA-34-284-s077.png]

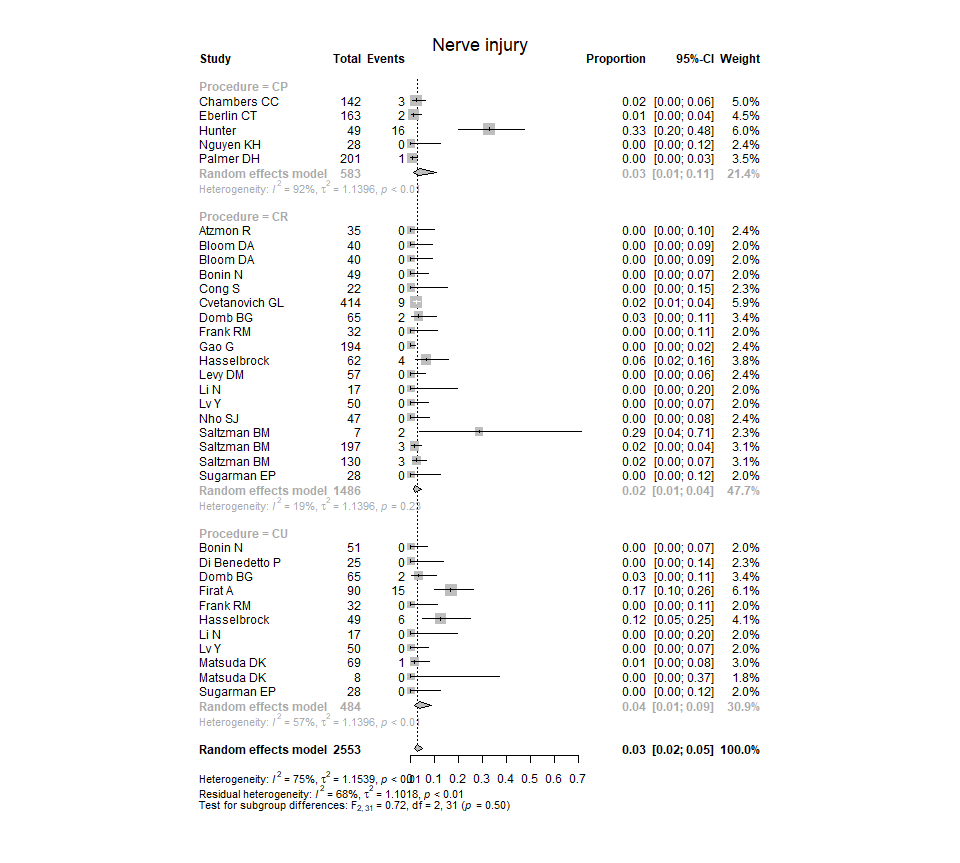

Supplement: Supplementary file 42 — Suppl Figure 42 Forestplot_Nerve injury. [file KSA-34-284-s016.png]

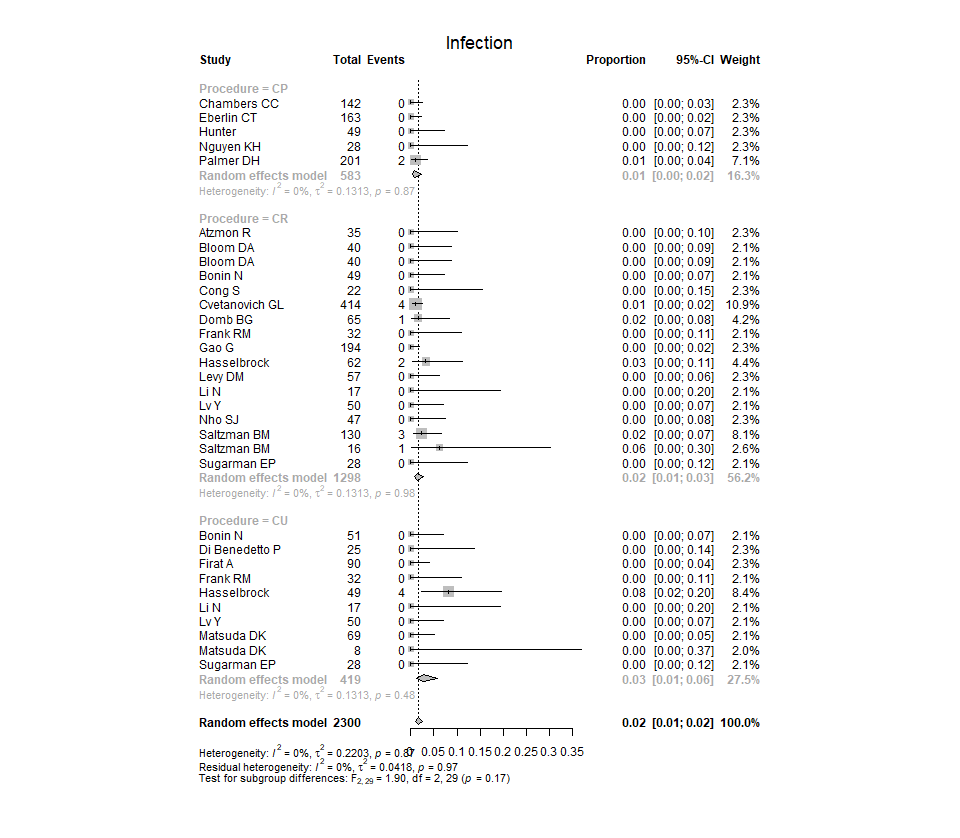

Supplement: Supplementary file 43 — Suppl Figure 43 Forestplot_Infection. [file KSA-34-284-s024.png]

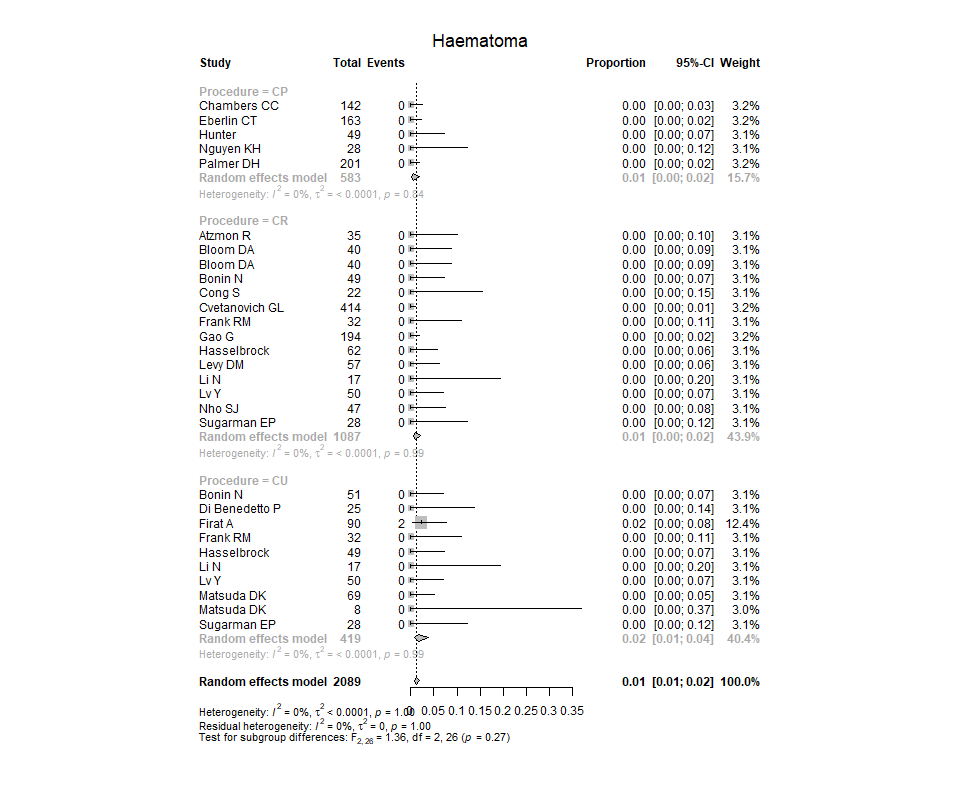

Supplement: Supplementary file 44 — Suppl Figure 44 Forestplot_Haematoma. [file KSA-34-284-s070.png]

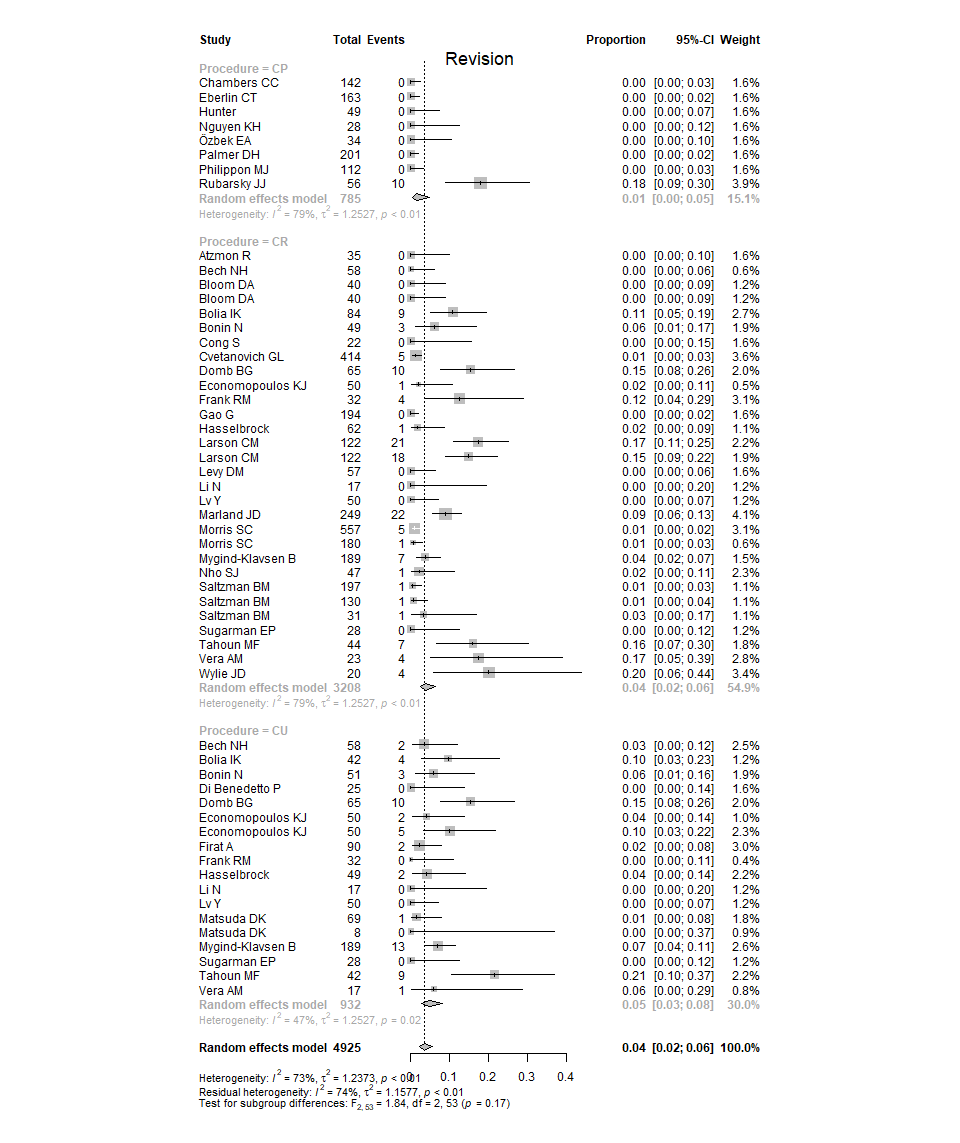

Supplement: Supplementary file 45 — Suppl Figure 45 Forestplot_Revision. [file KSA-34-284-s034.png]

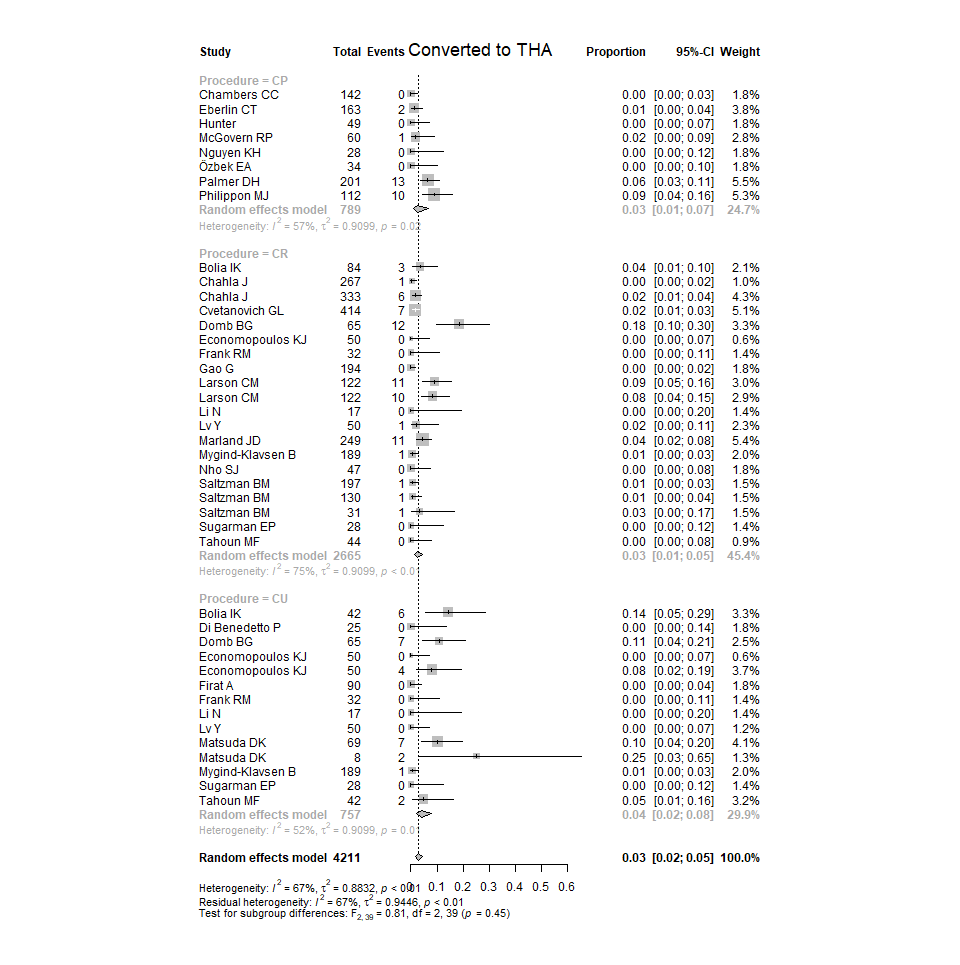

Supplement: Supplementary file 46 — Suppl Figure 46 Forestplot_Converted to THA. [file KSA-34-284-s015.png]

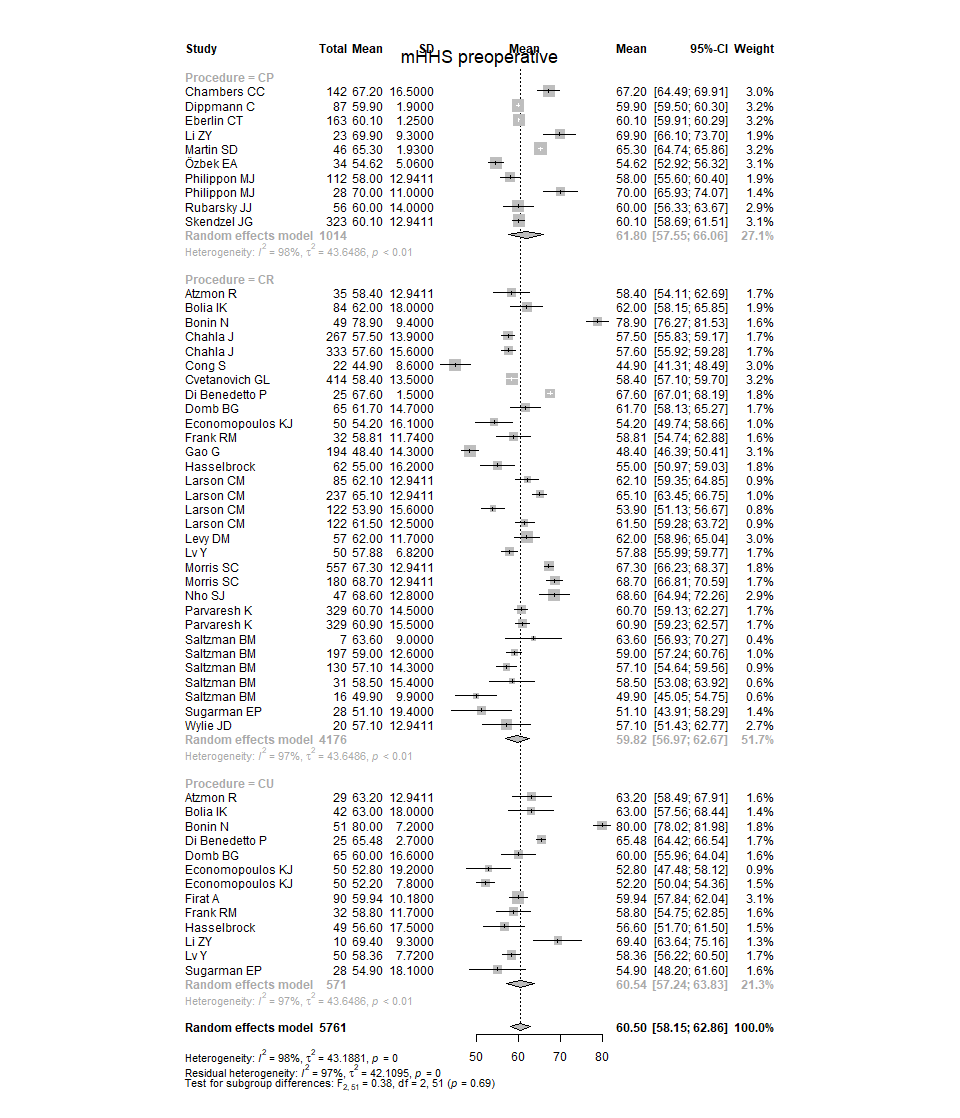

Supplement: Supplementary file 47 — Suppl Figure 47 Forestplot_mHHS preoperative. [file KSA-34-284-s074.png]

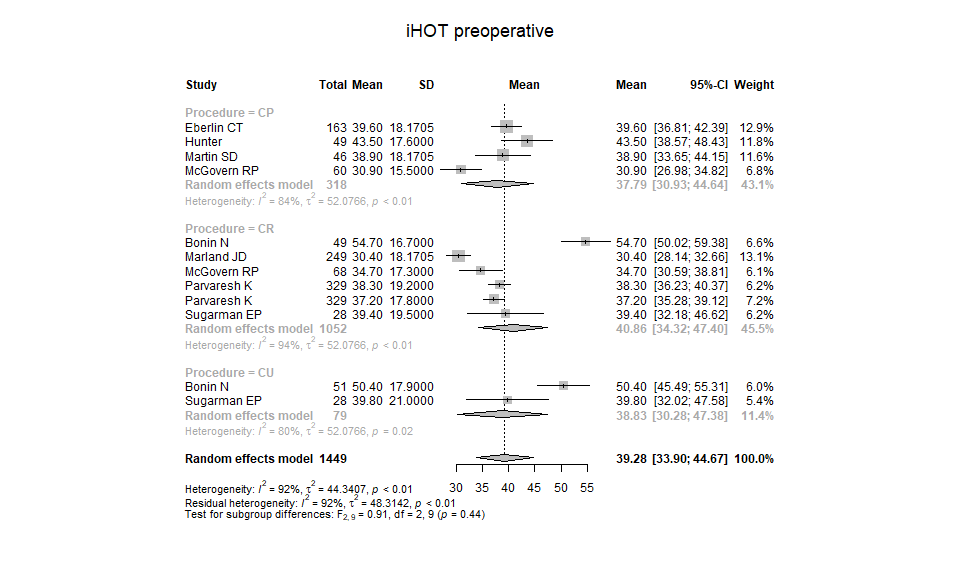

Supplement: Supplementary file 48 — Suppl Figure 48 Forestplot_iHOT preoperative. [file KSA-34-284-s056.png]

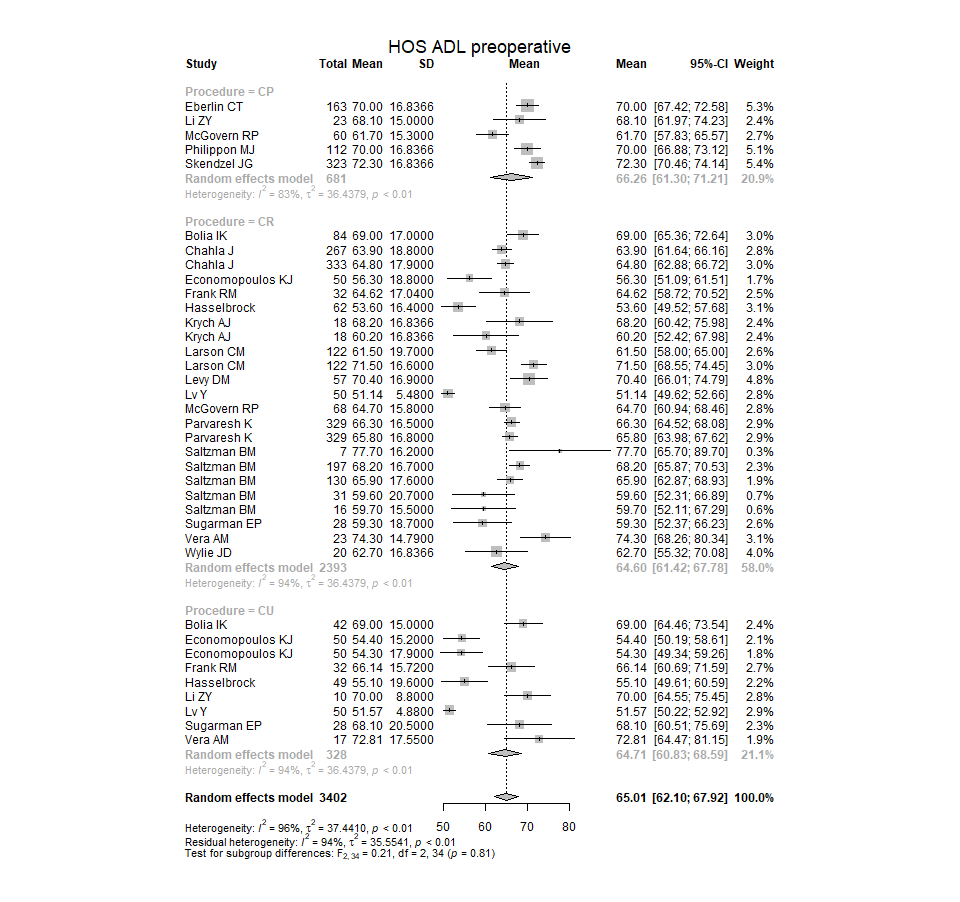

Supplement: Supplementary file 49 — Suppl Figure 49 Forestplot_HOS ADL preoperative. [file KSA-34-284-s008.png]

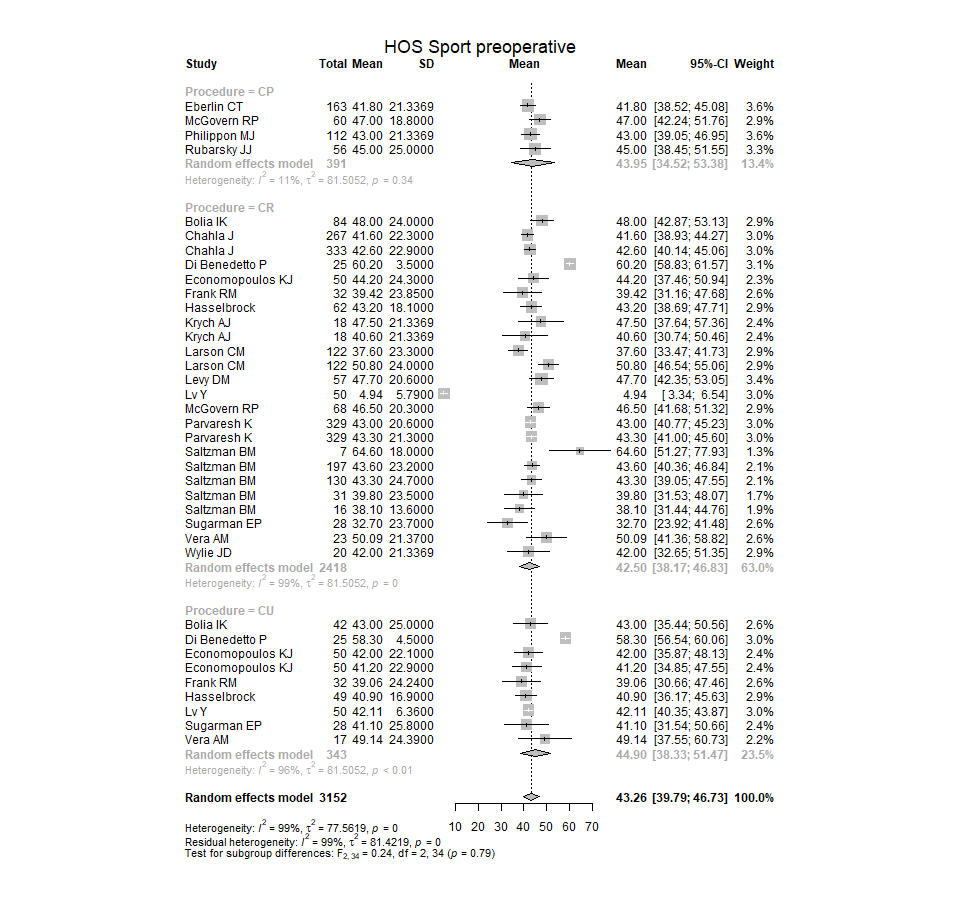

Supplement: Supplementary file 50 — Suppl Figure 50 Forestplot_HOS Sport preoperative. [file KSA-34-284-s032.png]

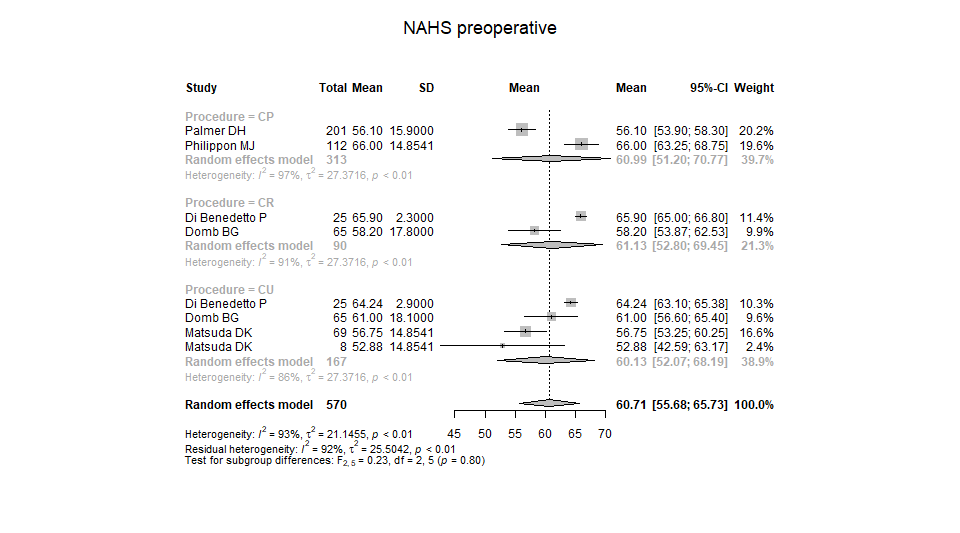

Supplement: Supplementary file 51 — Suppl Figure 51 Forestplot_NAHS preoperative. [file KSA-34-284-s048.png]

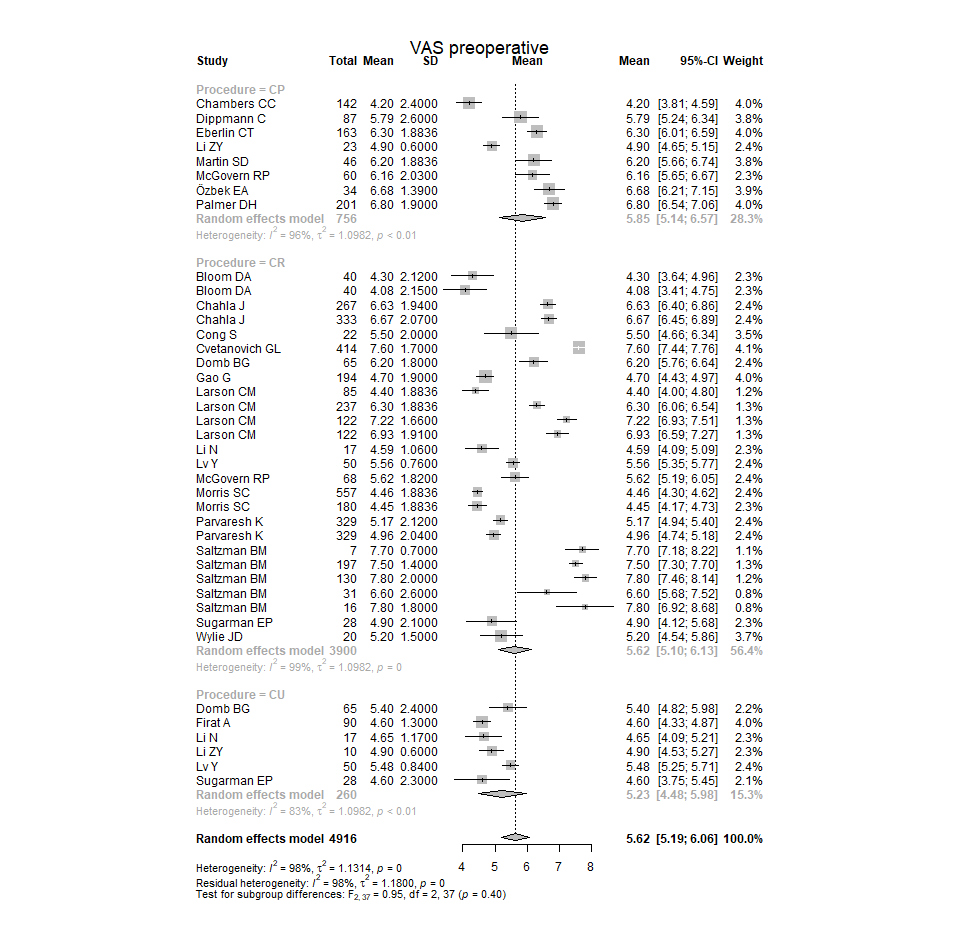

Supplement: Supplementary file 52 — Suppl Figure 52 Forestplot_VAS preoperative. [file KSA-34-284-s026.png]

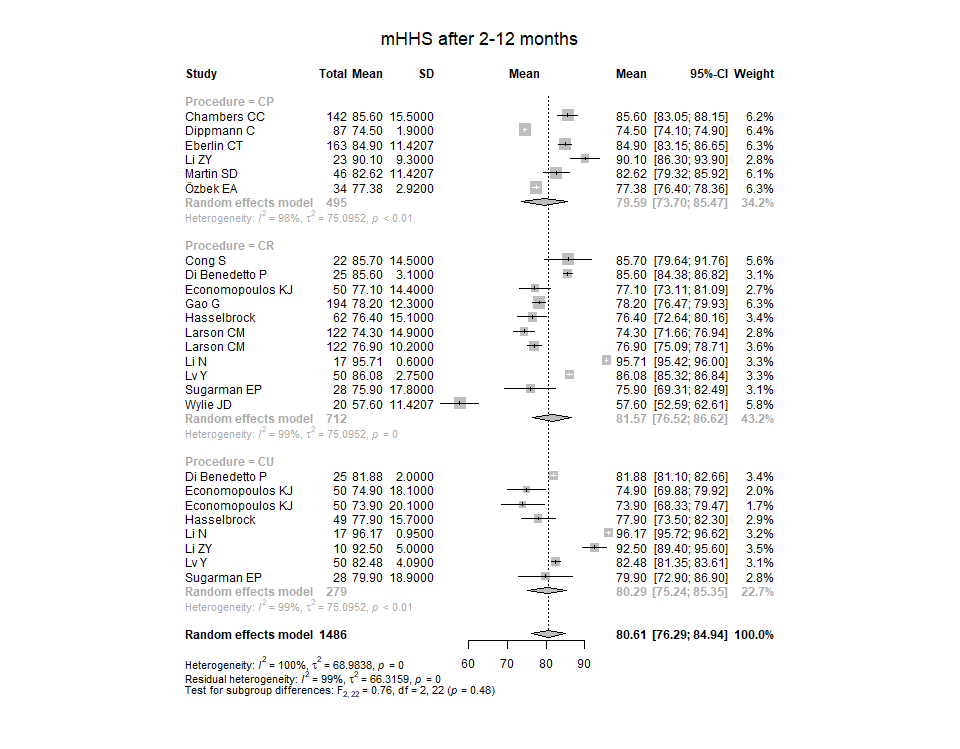

Supplement: Supplementary file 53 — Suppl Figure 53 Forestplot_mHHS after 2‐12 months. [file KSA-34-284-s006.png]

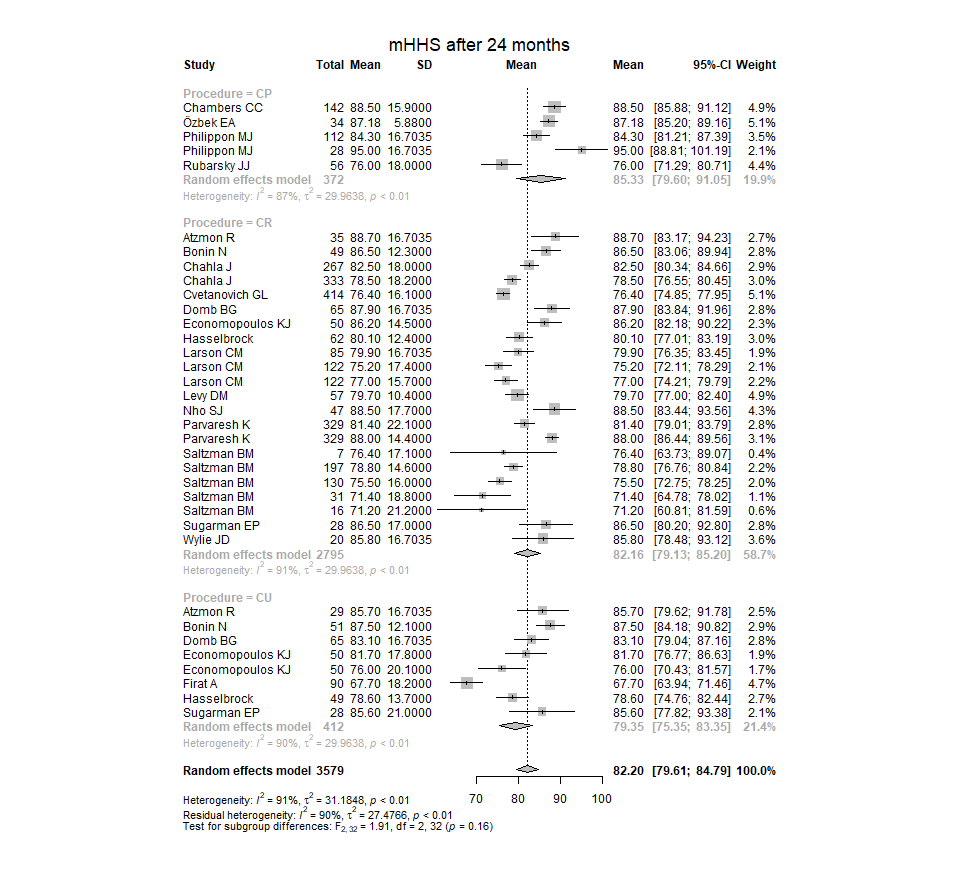

Supplement: Supplementary file 54 — Suppl Figure 54 Forestplot_mHHS after 24 months. [file KSA-34-284-s040.png]

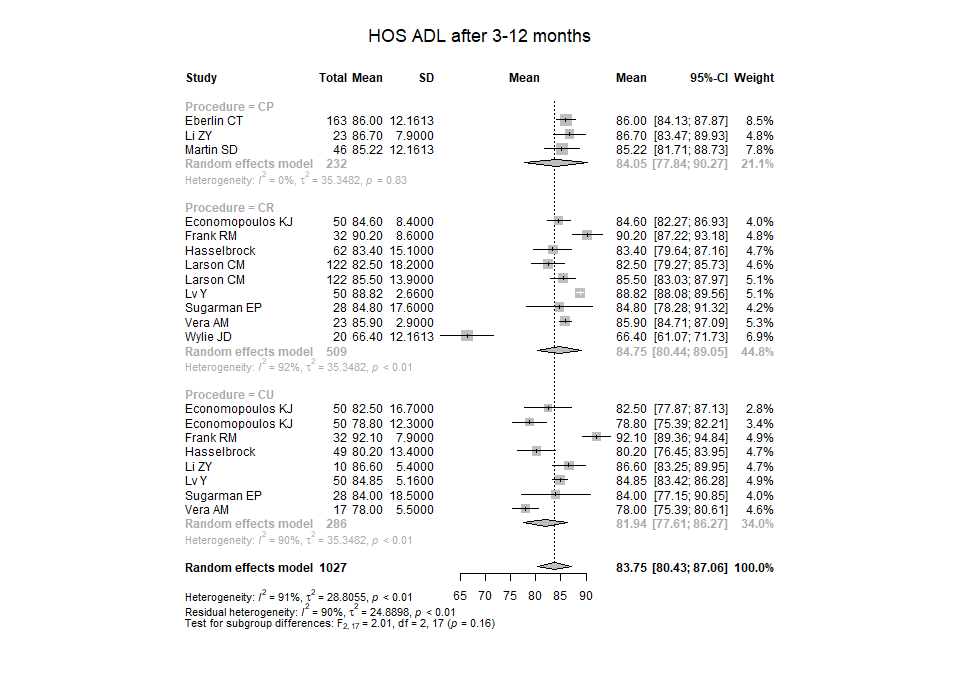

Supplement: Supplementary file 55 — Suppl Figure 55 Forestplot_HOS ADL after 3‐12 months. [file KSA-34-284-s050.png]

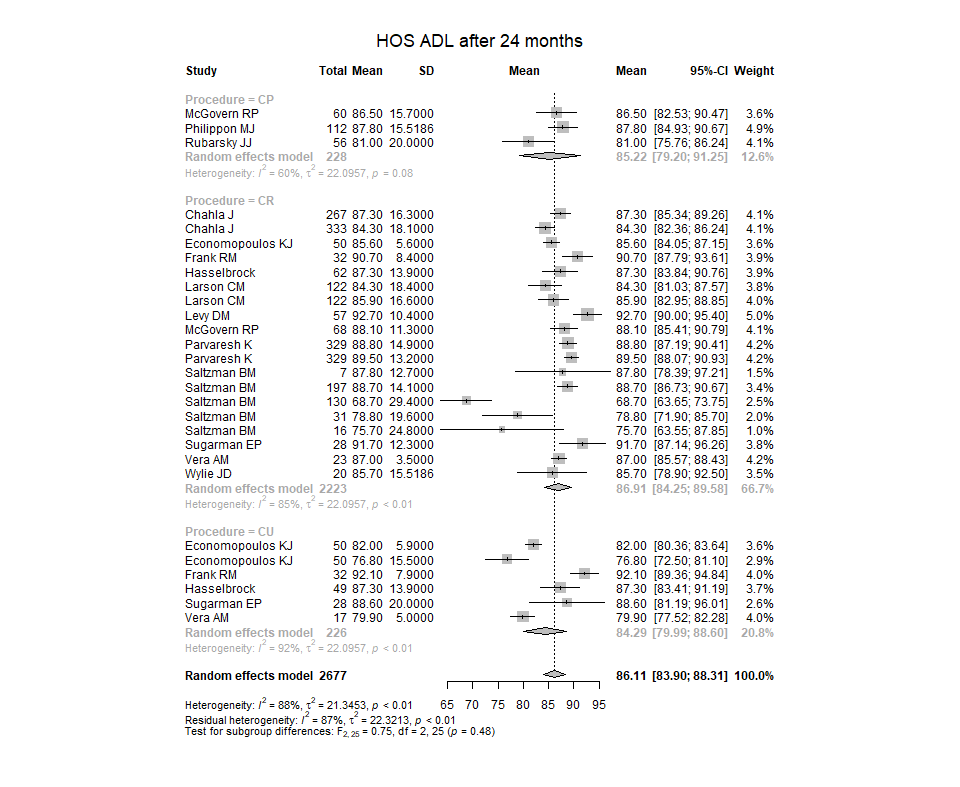

Supplement: Supplementary file 56 — Suppl Figure 56 Forestplot_HOS ADL after 24 months. [file KSA-34-284-s023.png]

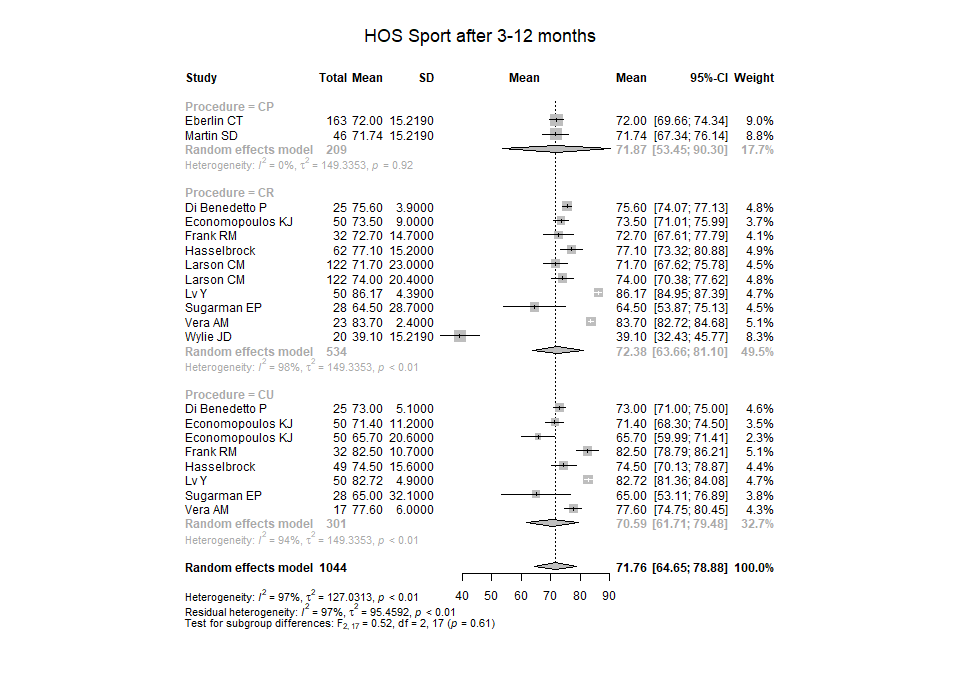

Supplement: Supplementary file 57 — Suppl Figure 57 Forestplot_HOS Sport after 3 months. [file KSA-34-284-s065.png]

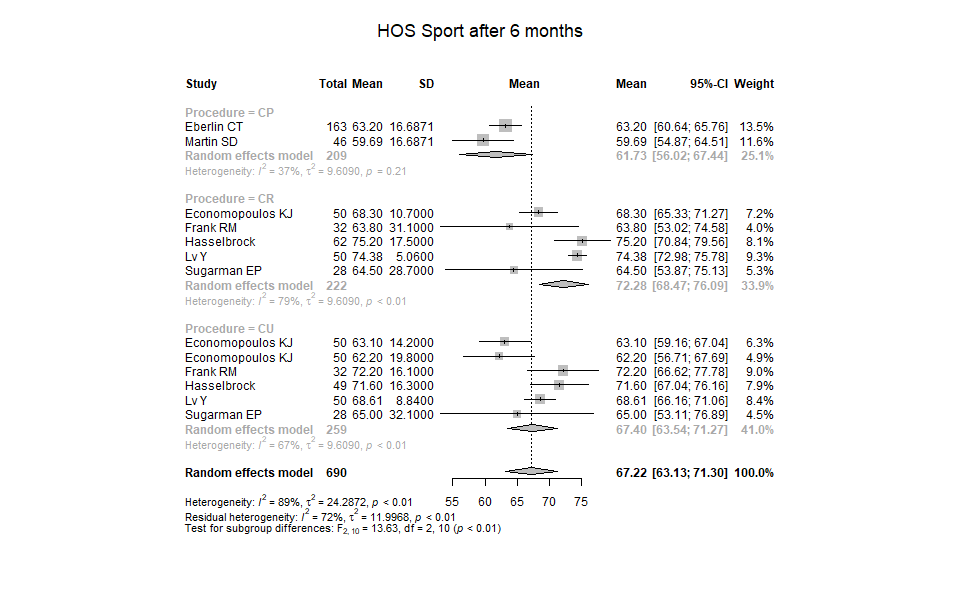

Supplement: Supplementary file 58 — Suppl Figure 58 Forestplot_HOS Sport after 6 months. [file KSA-34-284-s018.png]

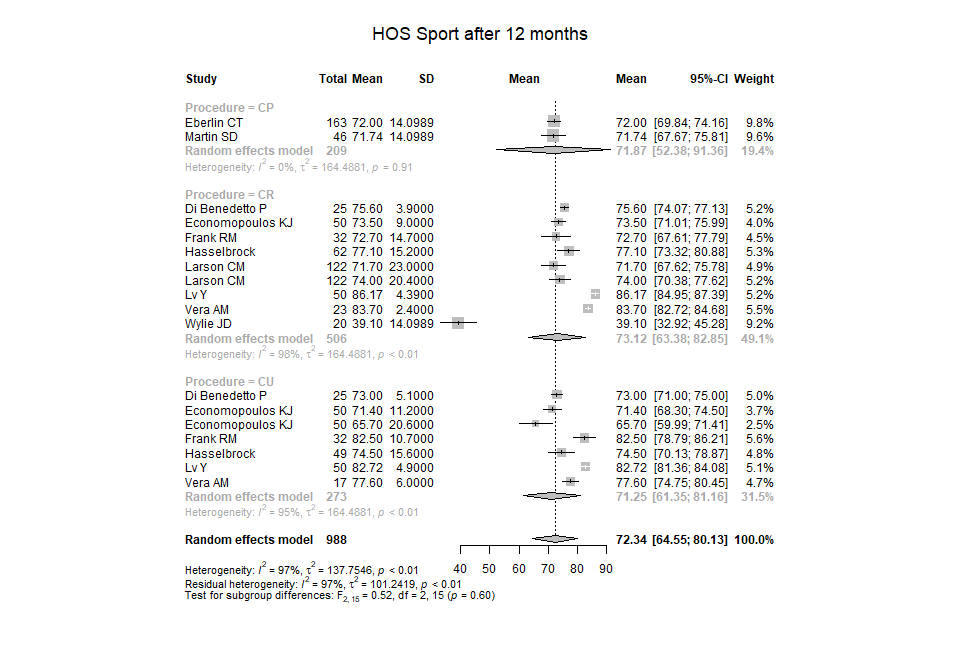

Supplement: Supplementary file 59 — Suppl Figure 59 Forestplot_HOS Sport after 12 months. [file KSA-34-284-s027.png]

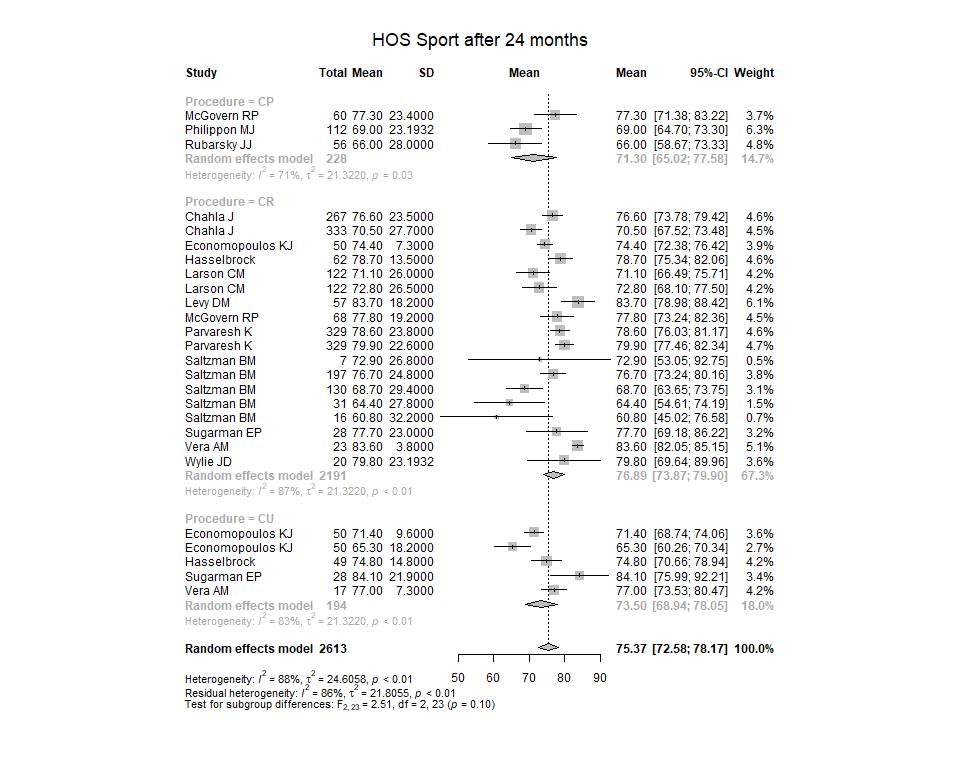

Supplement: Supplementary file 60 — Suppl Figure 60 Forestplot_HOS Sport after 24 months. [file KSA-34-284-s062.png]

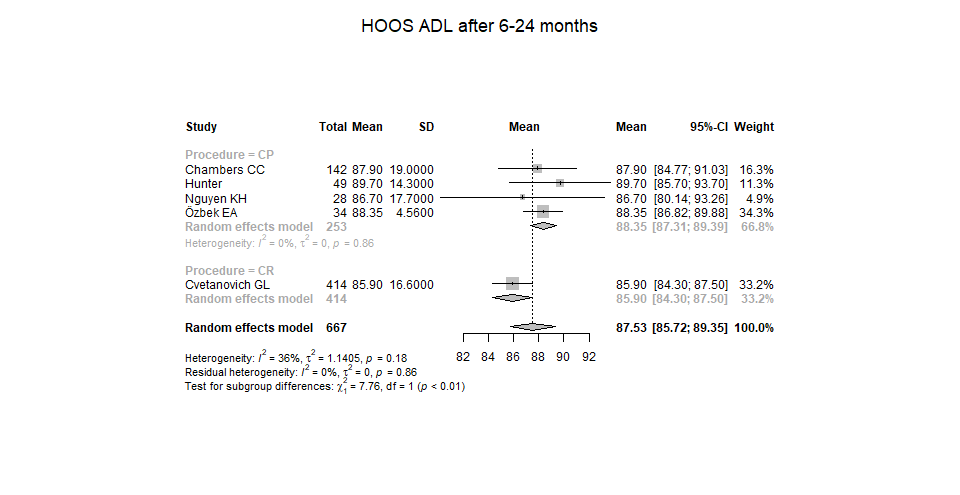

Supplement: Supplementary file 61 — Suppl Figure 61 Forestplot_HOOS ADL after 6‐24 months. [file KSA-34-284-s010.png]

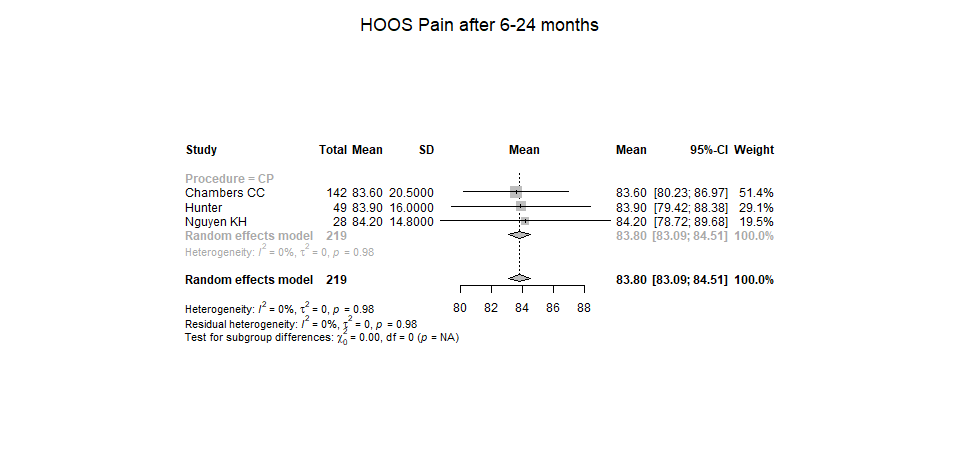

Supplement: Supplementary file 62 — Suppl Figure 62 Forestplot_HOOS Pain after 6‐24 months. [file KSA-34-284-s047.png]

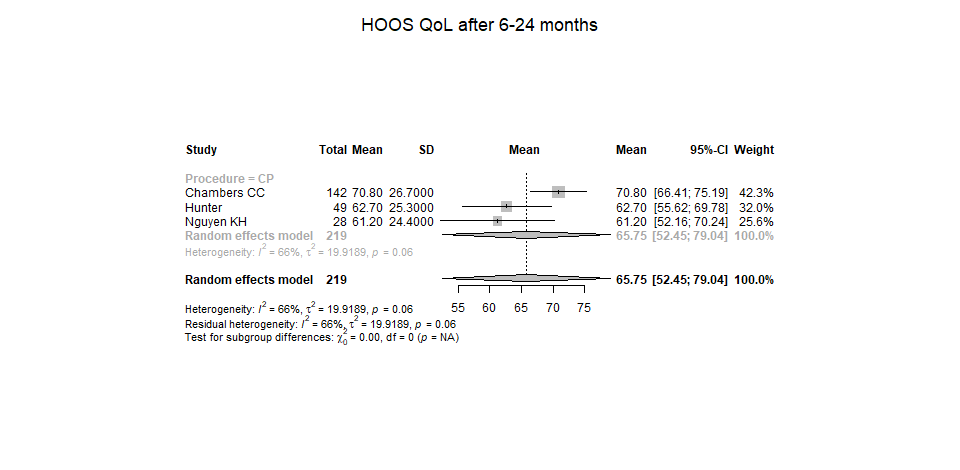

Supplement: Supplementary file 63 — Suppl Figure 63 Forestplot_HOOS QoL after 6‐24 months. [file KSA-34-284-s014.png]

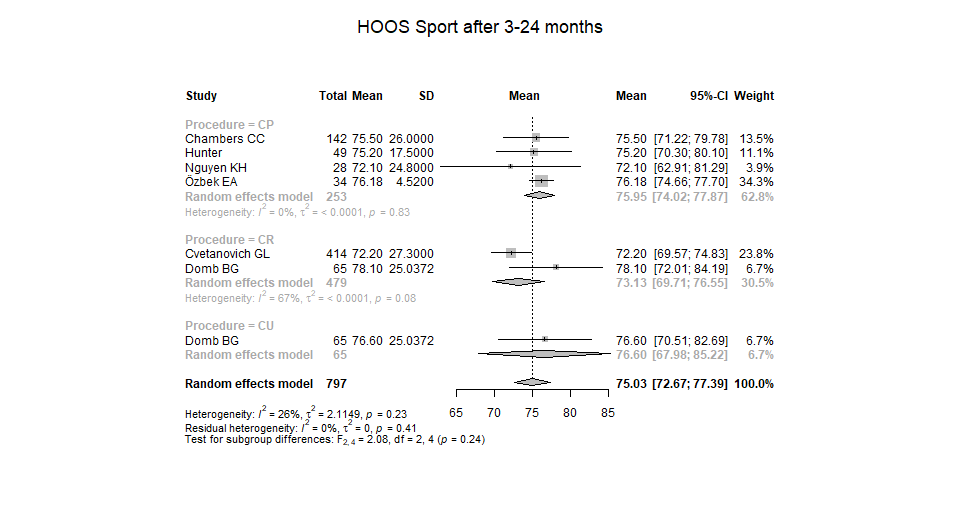

Supplement: Supplementary file 64 — Suppl Figure 64 Forestplot_HOOS Sport after 3‐24 months. [file KSA-34-284-s063.png]

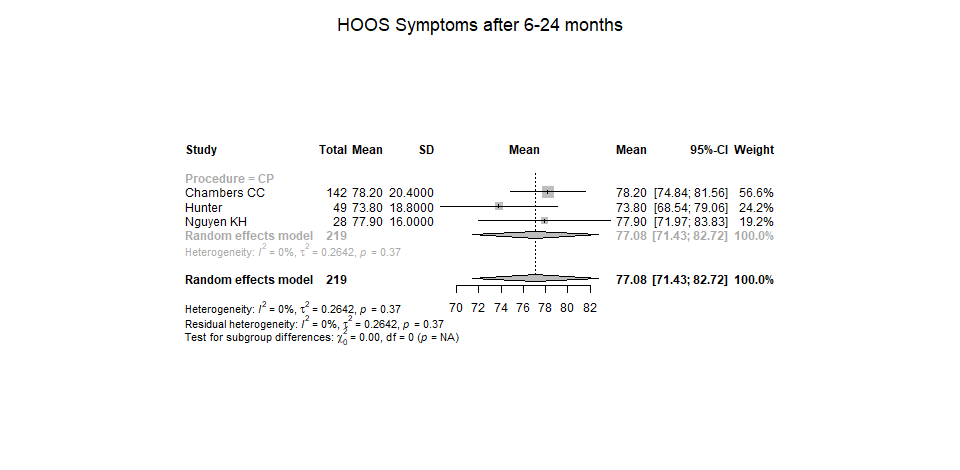

Supplement: Supplementary file 65 — Suppl Figure 65 Forestplot_HOOS Symptoms after 6‐24 months. [file KSA-34-284-s012.png]

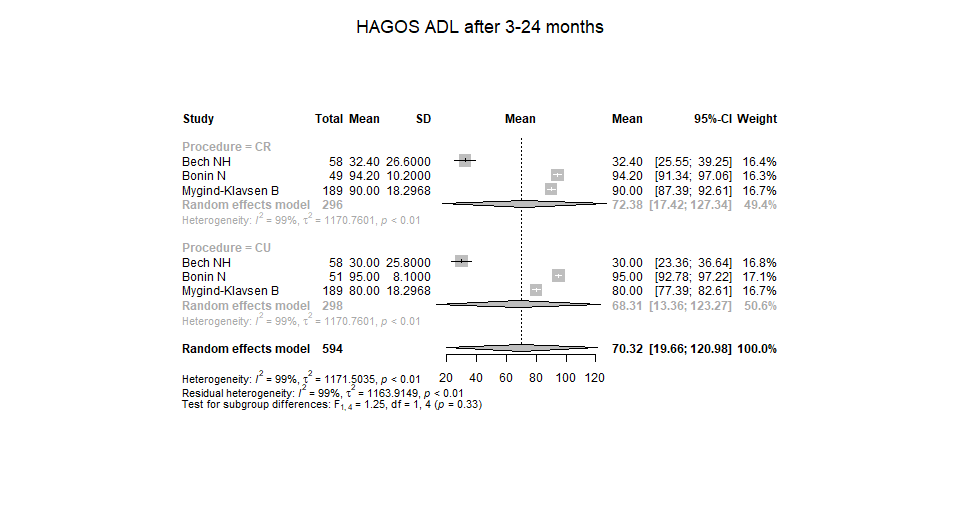

Supplement: Supplementary file 66 — Suppl Figure 66 Forestplot_HAGOS ADL after 3‐24 months. [file KSA-34-284-s011.png]

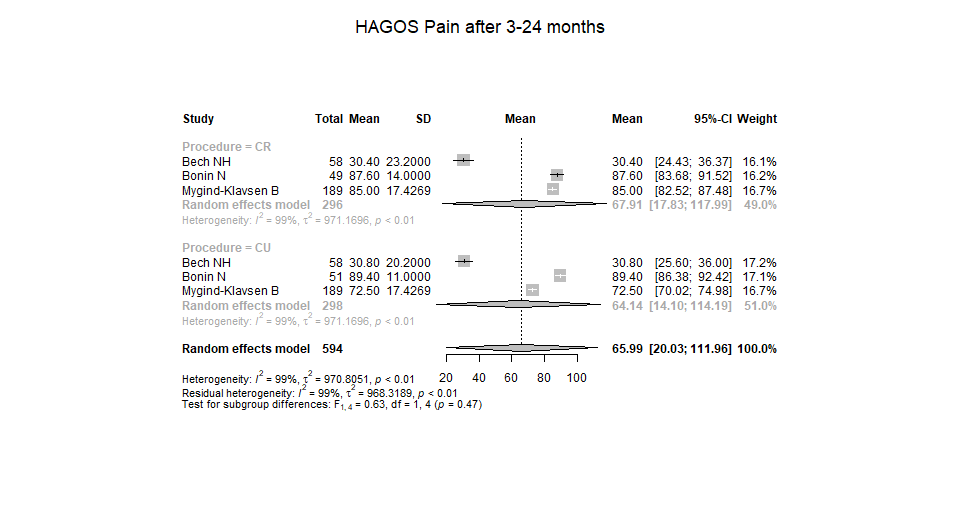

Supplement: Supplementary file 67 — Suppl Figure 67 Forestplot_HAGOS Pain after 3‐24 months. [file KSA-34-284-s044.png]

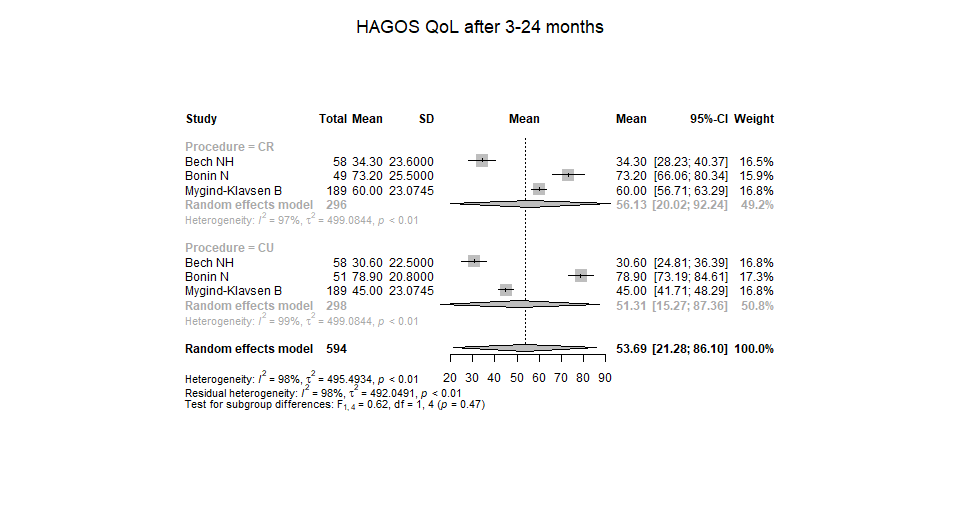

Supplement: Supplementary file 68 — Suppl Figure 68 Forestplot_HAGOS QoL after 3‐24 months. [file KSA-34-284-s069.png]

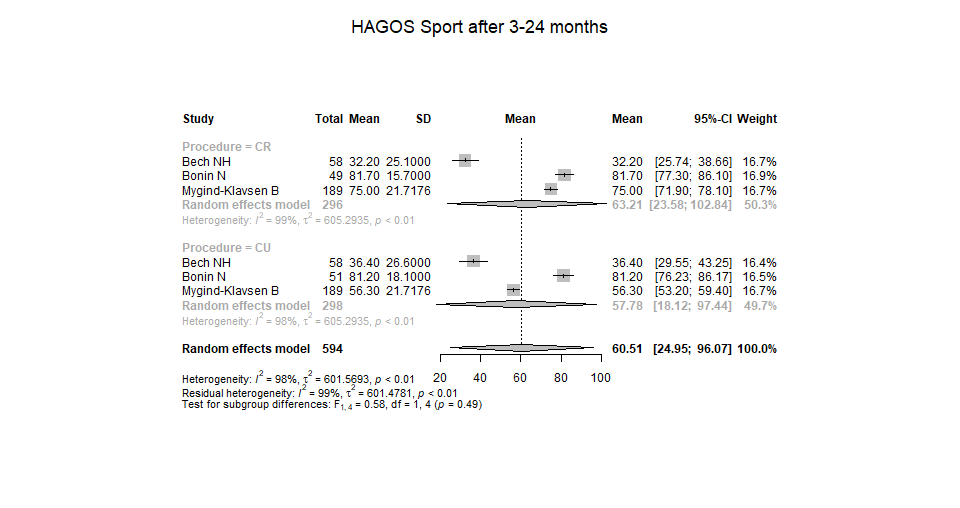

Supplement: Supplementary file 69 — Suppl Figure 69 Forestplot_HAGOS Sport after 3‐24 months. [file KSA-34-284-s031.png]

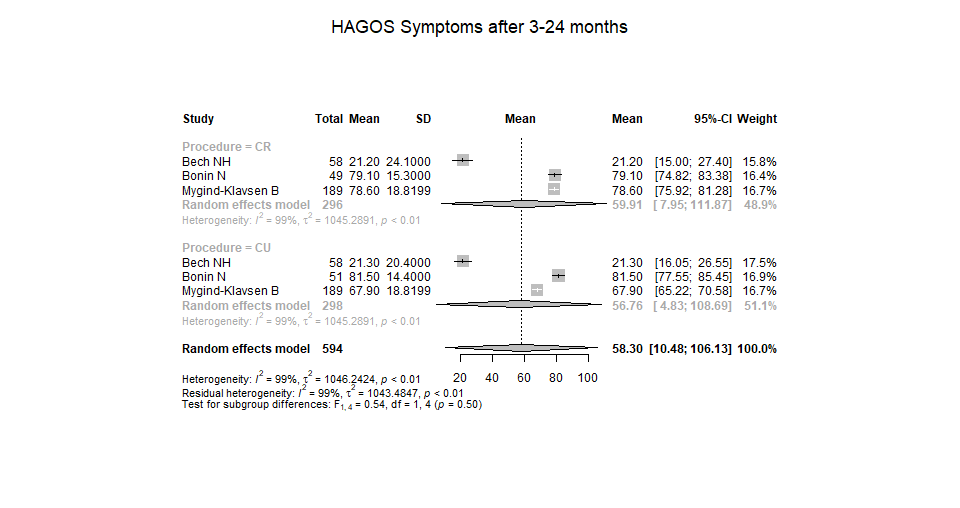

Supplement: Supplementary file 70 — Suppl Figure 70 Forestplot_HAGOS Symptoms after 3‐24 months. [file KSA-34-284-s042.png]

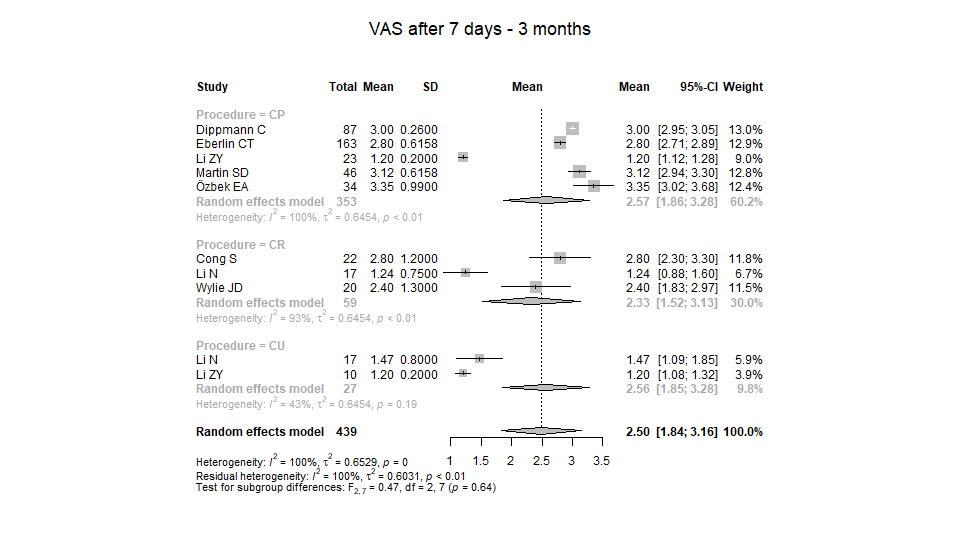

Supplement: Supplementary file 71 — Suppl Figure 71 Forestplot_VAS after 7 days ‐ 3 months. [file KSA-34-284-s061.png]

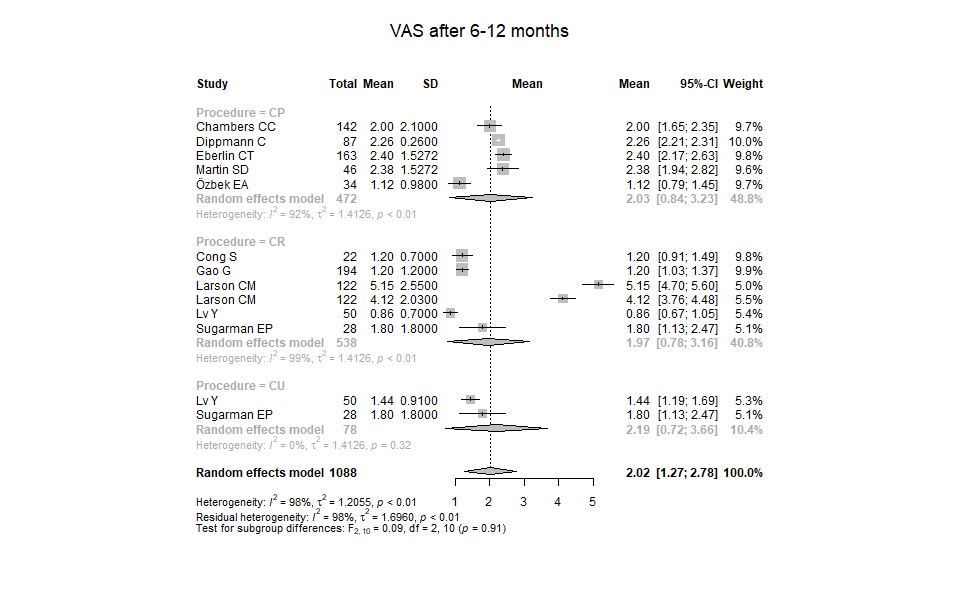

Supplement: Supplementary file 72 — Suppl Figure 72 Forestplot_VAS after 6‐12 months. [file KSA-34-284-s054.png]

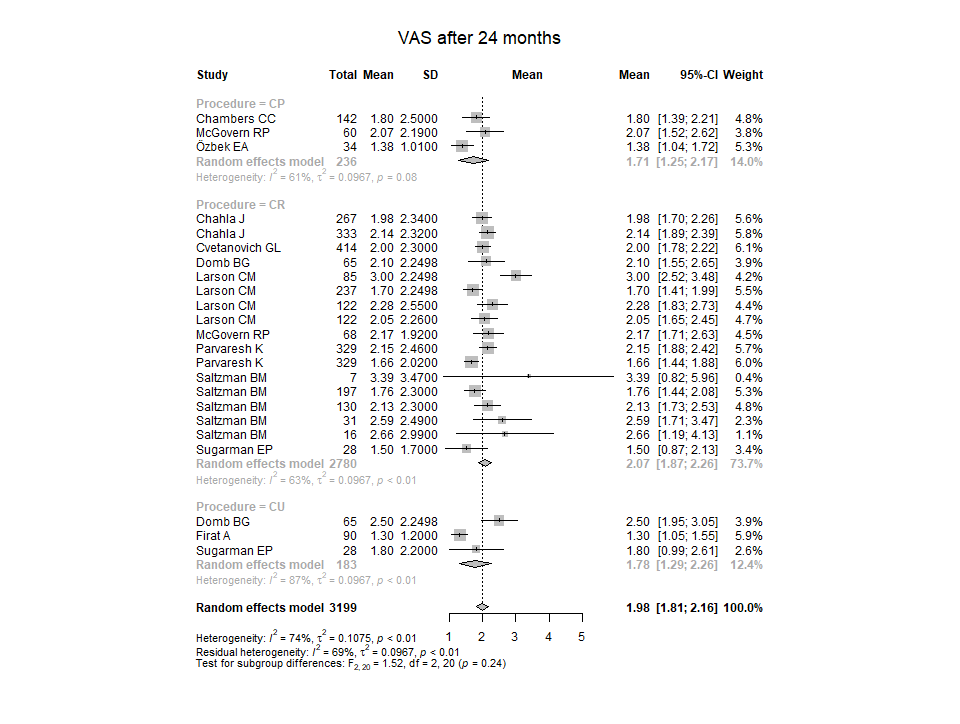

Supplement: Supplementary file 73 — Suppl Figure 73 Forestplot_VAS after 24 months. [file KSA-34-284-s021.png]
