# Supplementary material for: Capsular management strategies in hip arthroscopy for femoroacetabular impingement syndrome: A multilevel meta‐analysis
Source: Knee Surg Sports Traumatol Arthrosc. 2025 Oct 17;34(1):284–308. doi: 10.1002/ksa.70094 (PMC12747625; doi:10.1002/ksa.70094)
Supplement: Supplementary file 75 — Suppl Table 2 Preoperative. [file KSA-34-284-s058.docx]

|  | **Primary studies, N** | **Hips, N** | **Mean value** | **CIs** | **τ^2^** | **I^2^** | **Heterogenity p** | **Difference p** |
| --- | --- | --- | --- | --- | --- | --- | --- | --- |
| **Preoperative mHHS** | | | | | | | | |
| **Total** | 54 | 5761 | 60.50 | 58.15 ; 62.86 | 43.19 | 0.98 | < 0.0001 *** | 0.6883 |
| **CP** | 10 | 1014 | 61.80 | 57.55 ; 66.06 | 43.65 | 0.98 | < 0.0001 *** |  |
| **CR** | 31 | 4176 | 59.82 | 56.97 ; 62.67 | 43.65 | 0.97 | < 0.0001 *** |  |
| **CU** | 13 | 571 | 60.54 | 57.24 ; 63.83 | 43.65 | 0.97 | < 0.0001 *** |  |
| **Preoperative iHOT** | | | | | | | | |
| **Total** | 12 | 1449 | 39.28 | 33.9 ; 44.67 | 44.34 | 0.92 | < 0.0001 *** | 0.4354 |
| **CP** | 4 | 318 | 37.79 | 30.93 ; 44.64 | 52.08 | 0.84 | 0.0003 *** |  |
| **CR** | 6 | 1052 | 40.86 | 34.32 ; 47.4 | 52.08 | 0.94 | < 0.0001 *** |  |
| **CU** | 2 | 79 | 38.83 | 30.28 ; 47.38 | 52.08 | 0.80 | 0.0239 * |  |
| **Preoperative HOS ADL** | | | | | | | | |
| **Total** | 37 | 3402 | 65.01 | 62.1 ; 67.92 | 37.44 | 0.96 | < 0.0001 *** | 0.8117 |
| **CP** | 5 | 681 | 66.26 | 61.3 ; 71.21 | 36.44 | 0.83 | < 0.0001 *** |  |
| **CR** | 23 | 2393 | 64.60 | 61.42 ; 67.78 | 36.44 | 0.94 | < 0.0001 *** |  |
| **CU** | 9 | 328 | 64.71 | 60.83 ; 68.59 | 36.44 | 0.94 | < 0.0001 *** |  |
| **Preoperative HOS SSS** | | | | | | | | |
| **Total** | 37 | 3152 | 43.26 | 39.79 ; 46.73 | 77.56 | 0.99 | < 0.0001 *** | 0.7893 |
| **CP** | 4 | 391 | 43.95 | 34.52 ; 53.38 | 81.51 | 0.11 | 0.337 |  |
| **CR** | 24 | 2418 | 42.50 | 38.17 ; 46.83 | 81.51 | 0.99 | < 0.0001 *** |  |
| **CU** | 9 | 343 | 44.90 | 38.33 ; 51.47 | 81.51 | 0.96 | < 0.0001 *** |  |
| **Preoperative NAHS** | | | | | | | | |
| **Total** | 8 | 570 | 60.71 | 55.68 ; 65.73 | 21.15 | 0.93 | < 0.0001 *** | 0.7988 |
| **CP** | 2 | 313 | 60,99 | 51.2 ; 70.77 | 27.37 | 0.97 | < 0.0001 *** |  |
| **CR** | 2 | 90 | 61.13 | 52.8 ; 69.45 | 27.37 | 0.91 | 0.0006 *** |  |
| **CU** | 4 | 167 | 60.13 | 52.07 ; 68.19 | 27.37 | 0.86 | 0.0001 *** |  |
| **Preoperative VAS** | | | | | | | | |
| **Total** | 40 | 4916 | 5.62 | 5.19 ; 6.06 | 1.13 | 0.98 | < 0.0001 *** | 0.395 |
| **CP** | 8 | 756 | 5.85 | 5.14 ; 6.57 | 1.10 | 0.96 | < 0.0001 *** |  |
| **CR** | 26 | 3900 | 5.62 | 5.1 ; 6.13 | 1.10 | 0.99 | < 0.0001 *** |  |
| **CU** | 6 | 260 | 5.23 | 4.48 ; 5.98 | 1.10 | 0.83 | < 0.0001 *** |  |
