# Supplementary material for: Capsular management strategies in hip arthroscopy for femoroacetabular impingement syndrome: A multilevel meta‐analysis
Source: Knee Surg Sports Traumatol Arthrosc. 2025 Oct 17;34(1):284–308. doi: 10.1002/ksa.70094 (PMC12747625; doi:10.1002/ksa.70094)
Supplement: Supplementary file 76 — Suppl Table 3 Additional outcomes. [file KSA-34-284-s071.docx]

|  | **Primary studies, N** | | **Hips, N** | | **Mean value** | | **CIs** | | **τ^2^** | | **I^2^** | **Heterogenity p** | | **Difference p** |
| --- | --- | --- | --- | --- | --- | --- | --- | --- | --- | --- | --- | --- | --- | --- |
| **mHHS 2-12 months postoperatively** | | | | | | | | | | | | | | |
| **Total** | 25 | | 1486 | | 80.61 | | 76.29 ; 84.94 | | 68.98 | | 1.00 | < 0.0001 *** | | 0.4818 |
| **CP** | 6 | | 495 | | 79.59 | | 73.7 ; 85.47 | | 75.10 | | 0.98 | < 0.0001 *** | |  |
| **CR** | 11 | | 712 | | 81.57 | | 76.52 ; 86.62 | | 75.10 | | 0.99 | < 0.0001 *** | |  |
| **CU** | 8 | | 279 | | 80.29 | | 75.24 ; 85.35 | | 75.10 | | 0.99 | < 0.0001 *** | |  |
| **mHHS 24 months postoperatively** | | | | | | | | | | | | | | |
| **Total** | 35 | | 3579 | | 82.20 | | 79.61 ; 84.79 | | 31.18 | | 0.91 | < 0.0001 *** | | 0.1649 |
| **CP** | 5 | | 372 | | 85.33 | | 79.6 ; 91.05 | | 29.96 | | 0.87 | < 0.0001 *** | |  |
| **CR** | 22 | | 2795 | | 82.16 | | 79.13 ; 85.2 | | 29.96 | | 0.91 | < 0.0001 *** | |  |
| **CU** | 8 | | 412 | | 79.35 | | 75.35 ; 83.35 | | 29.96 | | 0.90 | < 0.0001 *** | |  |
| **HOS ADL 3-12 months postoperatively** | | | | | | | | | | | | | | |
| **Total** | 20 | | 1027 | | 83.75 | | 80.43 ; 87.06 | | 28.81 | | 0.91 | < 0.0001 *** | | 0.1644 |
| **CP** | 3 | | 232 | | 84.05 | | 77.84 ; 90.27 | | 35.35 | | 0.00 | 0.8312 | |  |
| **CR** | 9 | | 509 | | 84.75 | | 80.44 ; 89.05 | | 35.35 | | 0.92 | < 0.0001 *** | |  |
| **CU** | 8 | | 286 | | 81.94 | | 77.61 ; 86.27 | | 35.35 | | 0.90 | < 0.0001 *** | |  |
| **HOS ADL 24 months postoperatively** | | | | | | | | | | | | | | |
| **Total** | 28 | | 2677 | | 86.11 | | 83.9 ; 88.31 | | 21.35 | | 0.88 | < 0.0001 *** | | 0.4834 |
| **CP** | 3 | | 228 | | 85.22 | | 79.2 ; 91.25 | | 22.10 | | 0.60 | 0.0825 | |  |
| **CR** | 19 | | 2223 | | 86.91 | | 84.25 ; 89.58 | | 22.10 | | 0.85 | < 0.0001 *** | |  |
| **CU** | 6 | | 226 | | 84.29 | | 79.99 ; 88.6 | | 22.10 | | 0.92 | < 0.0001 *** | |  |
| **HOS SSS 3-12 months postoperatively** | | | | | | | | | | | | | | |
| **Total** | 20 | | 1044 | | 71.76 | | 64.65 ; 78.88 | | 127.03 | | 0.97 | < 0.0001 *** | | 0.6052 |
| **CP** | 2 | | 209 | | 71.87 | | 53.45 ; 90.3 | | 149.34 | | 0 | 0.9185 | |  |
| **CR** | 10 | | 534 | | 72.38 | | 63.66 ; 81.1 | | 149.34 | | 0.98 | < 0.0001 *** | |  |
| **CU** | 8 | | 301 | | 70.59 | | 61.71 ; 79.48 | | 149.34 | | 0.94 | < 0.0001 *** | |  |
| **HOS SSS 6 months postoperatively** | | | | | | | | | | | | | | |
| **Total** | 13 | | 690 | | 67.22 | | 63.13 ; 71.3 | | 24.29 | | 0.89 | < 0.0001 *** | | 0.0014 ** |
| **CP** | 2 | | 209 | | 61.73 | | 56.02 ; 67.44 | | 9.61 | | 0.37 | 0.2077 | |  |
| **CR** | 5 | | 222 | | 72.28 | | 68.47 ; 76.09 | | 9.61 | | 0.79 | 0.0006 *** | |  |
| **CU** | 6 | | 259 | | 67.40 | | 63.54 ; 71.27 | | 9.61 | | 0.67 | 0.0098 ** | |  |
| **HOS SSS 12 months postoperatively** | | | | | | | | | | | | | | |
| **Total** | 18 | | 988 | | 72.34 | | 64.55 ; 80.13 | | 137.75 | | 0.97 | < 0.0001 *** | | 0.6041 |
| **CP** | 2 | | 209 | | 71.87 | | 52.38 ; 91.36 | | 164.49 | | 0.00 | 0.912 | |  |
| **CR** | 9 | | 506 | | 73.12 | | 63.38 ; 82.85 | | 164.49 | | 0.98 | < 0.0001 *** | |  |
| **CU** | 7 | | 273 | | 71.25 | | 61.35 ; 81.16 | | 164.49 | | 0.95 | < 0.0001 *** | |  |
| **HOS SSS 24 months postoperatively** | | | | | | | | | | | | | | |
| **Total** | 26 | | 2613 | | 75.37 | | 72.58 ; 78.17 | | 24.61 | | 0.88 | < 0.0001 *** | | 0.1035 |
| **CP** | 3 | | 228 | | 71.30 | | 65.02 ; 77.58 | | 21.32 | | 0.71 | 0.0310 * | |  |
| **CR** | 18 | | 2191 | | 76.89 | | 73.87 ; 79.9 | | 21.32 | | 0.87 | < 0.0001 *** | |  |
| **CU** | 5 | | 194 | | 73.50 | | 68.94 ; 78.05 | | 21.32 | | 0.83 | 0.0001 *** | |  |
| **HOOS SSS 3-24 months postoperatively** | | | | | | | | | | | | | | |
| **Total** | | 7 | | 797 | | 75.03 | | 72.67 ; 77.39 | | 2.11 | 0.26 | | 0.2315 | 0.2407 |
| **CP** | | 4 | | 253 | | 75.95 | | 74.02 ; 77.87 | | 0.00 | 0.00 | | 0.8265 |  |
| **CR** | | 2 | | 479 | | 73.13 | | 69.71 ; 76.55 | | 0.00 | 0.67 | | 0.0812 |  |
| **CU** | | 1 | | 65 | | 76.60 | | 67.98 ; 85.22 | | 0.00 |  | | - |  |
| **VAS 7 days – 3 months postoperatively** | | | | | | | | | | | | | | |
| **Total** | | 10 | | 439 | | 2.50 | | 1.84 ; 3.16 | | 0.65 | 1.00 | | < 0.0001 *** | 0.6428 |
| **CP** | | 5 | | 353 | | 2.57 | | 1.86 ; 3.28 | | 0.65 | 1.00 | | < 0.0001 *** |  |
| **CR** | | 3 | | 59 | | 2.33 | | 1.52 ; 3.13 | | 0.65 | 0.93 | | < 0.0001 *** |  |
| **CU** | | 2 | | 27 | | 2.56 | | 1.85 ; 3.28 | | 0.65 | 0.43 | | 0.1858 |  |
| **VAS 6-12 months postoperatively** | | | | | | | | | | | | | | |
| **Total** | | 13 | | 1088 | | 2.02 | | 1.27 ; 2.78 | | 1.21 | 0.98 | | < 0.0001 *** | 0.9108 |
| **CP** | | 5 | | 472 | | 2.03 | | 0.84 ; 3.23 | | 1.41 | 0.92 | | < 0.0001 *** |  |
| **CR** | | 6 | | 538 | | 1.97 | | 0.78 ; 3.16 | | 1.41 | 0.99 | | < 0.0001 *** |  |
| **CU** | | 2 | | 78 | | 2.19 | | 0.72 ; 3.66 | | 1.41 | 0.00 | | 0.3223 |  |
| **VAS 24 months postoperatively** | | | | | | | | | | | | | | |
| **Total** | | 23 | | 3199 | | 1.98 | | 1.81 ; 2.16 | | 0.11 | 0.74 | | < 0.0001 *** | 0.2429 |
| **CP** | | 3 | | 236 | | 1.71 | | 1.25 ; 2.17 | | 0.10 | 0.61 | | 0.0763 |  |
| **CR** | | 17 | | 2780 | | 2.07 | | 1.87 ; 2.26 | | 0.10 | 0.63 | | 0.0002 *** |  |
| **CU** | | 3 | | 183 | | 1.78 | | 1.29 ; 2.26 | | 0.10 | 0.87 | | 0.0004 *** |  |
