# Supplementary material for: Capsular management strategies in hip arthroscopy for femoroacetabular impingement syndrome: A multilevel meta‐analysis
Source: Knee Surg Sports Traumatol Arthrosc. 2025 Oct 17;34(1):284–308. doi: 10.1002/ksa.70094 (PMC12747625; doi:10.1002/ksa.70094)
Supplement: Supplementary file 77 — Supmat. [file KSA-34-284-s046.docx]

**SUPPLEMENTARY TABLES**

Supplementary Table 1: PRISMA Checklist. *PRISMA: Preferred Reporting Items for Systematic Reviews and Meta-Analyses;*

Supplementary Table 2: Summary of the preoperative outcome parameters. *Abbreviations: CI, confidence interval; CP: capsule preserved; CR: capsule repaired; CU: capsule unrepaired; mHHS: modified Harris Hip Score; iHOT:* *International Hip Outcome Tool; HOS‐ADL: Hip Outcome Score – Activities of Daily Living; HOS‐SSS: Hip Outcome Score ‐ Sports Subscale; NAHS: Non‐Arthritic Hip Score; VAS: visual analogue score; *Statistically significant. **Very statistically significant. ***Highly statistically significant.*

Supplementary Table 3: Summary of additional postoperative outcome parameters. *Abbreviations: CI, confidence interval; CP: capsule preserved; CR: capsule repaired; CU: capsule unrepaired; mHHS: modified Harris Hip Score; HOS‐ADL: Hip Outcome Score – Activities of Daily Living; HOS‐SSS: Hip Outcome Score ‐ Sports Subscale; VAS: visual analogue score; *Statistically significant. **Very statistically significant. ***Highly statistically significant.*

**SUPPLEMENTARY FIGURES**

Supplementary Figure 1: Funnel plot of the total postoperative mHHS. *mHHS: modified Harris Hip Score;*

Supplementary Figure 2: Funnel plot of the total postoperative iHOT. *iHOT: International Hip Outcome Tool;*

Supplementary Figure 3: Funnel plot of the total postoperative HOS ADL. *HOS‐ADL: Hip Outcome Score – Activities of Daily Living;*

Supplementary Figure 4: Funnel plot of the total postoperative HOS SSS. *HOS‐SSS: Hip Outcome Score ‐ Sports Subscale;*

Supplementary Figure 5: Funnel plot of the total postoperative NAHS. *NAHS: Non‐Arthritic Hip Score;*

Supplementary Figure 6: Funnel plot of the total postoperative VAS. *VAS: visual analogue score;*

Supplementary Figure 7: Funnel plot of the change in mHHS. *iHOT: International Hip Outcome Tool;*

Supplementary Figure 8: Funnel plot of the change in iHOT. *iHOT: International Hip Outcome Tool;*

Supplementary Figure 9: Funnel plot of the change in HOS ADL. *HOS‐ADL: Hip Outcome Score – Activities of Daily Living;*

Supplementary Figure 10: Funnel plot of the change in HOS SSS. *HOS‐SSS: Hip Outcome Score ‐ Sports Subscale;*

Supplementary Figure 11: Funnel plot of the change in NAHS. *NAHS: Non‐Arthritic Hip Score;*

Supplementary Figure 12: Funnel plot of the change in VAS. *VAS: visual analogue score;*

Supplementary Figure 13: Funnel plot of the functional MCID 3 months postoperatively. *MCID: minimal clinically important difference;*

Supplementary Figure 14: Funnel plot of the functional MCID 6 months postoperatively. *MCID: minimal clinically important difference;*

Supplementary Figure 15: Funnel plot of the functional MCID 12 months postoperatively. *MCID: minimal clinically important difference;*

Supplementary Figure 16: Funnel plot of the functional MCID 24 months postoperatively. *MCID: minimal clinically important difference;*

Supplementary Figure 17: Funnel plot of the pain MCID last follow-up. *MCID: minimal clinically important difference;*

Supplementary Figure 18: Funnel plot of the overall complications rate.

Supplementary Figure 19: Funnel plot of the DVT/PE rate. *DVT: deep vein thrombosis; PE: pulmonary embolism;*

Supplementary Figure 20: Funnel plot of the nerve injury rate.

Supplementary Figure 21: Funnel plot of the infection rate.

Supplementary Figure 22: Funnel plot of the haematoma rate.

Supplementary Figure 23: Funnel plot of the revision rate.

Supplementary Figure 24: Funnel plot of the THA conversion rate. *THA: total hip arthroplasty;*

Supplementary Figure 25: Forest plot of the total postoperative mHHS. *SD: standard deviation; mHHS: modified Harris Hip Score; CI: confidence interval; CP: capsule preserved; CR: capsule repaired; CU: capsule unrepaired;*

Supplementary Figure 26: Forest plot of the total postoperative iHOT. *SD: standard deviation; iHOT: International Hip Outcome Tool; CI: confidence interval; CP: capsule preserved; CR: capsule repaired; CU: capsule unrepaired;*

Supplementary Figure 27: Forest plot of the total postoperative HOS ADL. *SD: standard deviation; HOS‐ADL: Hip Outcome Score – Activities of Daily Living; CI: confidence interval; CP: capsule preserved; CR: capsule repaired; CU: capsule unrepaired;*

Supplementary Figure 28: Forest plot of the total postoperative HOS SSS. *SD: standard deviation; HOS‐SSS: Hip Outcome Score ‐ Sports Subscale; CI: confidence interval; CP: capsule preserved; CR: capsule repaired; CU: capsule unrepaired;*

Supplementary Figure 29: Forest plot of the total postoperative NAHS. *SD: standard deviation; NAHS: Non‐Arthritic Hip Score; CI: confidence interval; CP: capsule preserved; CR: capsule repaired; CU: capsule unrepaired;*

Supplementary Figure 30: Forest plot of the total postoperative VAS. *SD: standard deviation; VAS: visual analogue score; CI: confidence interval; CP: capsule preserved; CR: capsule repaired; CU: capsule unrepaired;*

Supplementary Figure 31: Forest plot of the change in iHOT. *SD: standard deviation; iHOT: International Hip Outcome Tool; CI: confidence interval; CP: capsule preserved; CR: capsule repaired; CU: capsule unrepaired;*

Supplementary Figure 32: Forest plot of the change in HOS ADL. *SD: standard deviation; HOS‐ADL: Hip Outcome Score – Activities of Daily Living; CI: confidence interval; CP: capsule preserved; CR: capsule repaired; CU: capsule unrepaired;*

Supplementary Figure 33: Forest plot of the change in HOS SSS. *SD: standard deviation; HOS‐SSS: Hip Outcome Score ‐ Sports Subscale; CI: confidence interval; CP: capsule preserved; CR: capsule repaired; CU: capsule unrepaired;*

Supplementary Figure 34: Forest plot of the change in NAHS. *SD: standard deviation; NAHS: Non‐Arthritic Hip Score; CI: confidence interval; CP: capsule preserved; CR: capsule repaired; CU: capsule unrepaired;*

Supplementary Figure 35: Forest plot of the change in VAS. *SD: standard deviation; VAS: visual analogue score; CI: confidence interval; CP: capsule preserved; CR: capsule repaired; CU: capsule unrepaired;*

Supplementary Figure 36: Forest plot of the functional MCID 3 months postoperatively. *SD: standard deviation; MCID: minimal clinically important difference; CI: confidence interval; CP: capsule preserved; CR: capsule repaired; CU: capsule unrepaired;*

Supplementary Figure 37: Forest plot of the functional MCID 6 months postoperatively. *SD: standard deviation; MCID: minimal clinically important difference; CI: confidence interval; CP: capsule preserved; CR: capsule repaired; CU: capsule unrepaired;*

Supplementary Figure 38: Forest plot of the functional MCID 24 months postoperatively. *SD: standard deviation; MCID: minimal clinically important difference; CI: confidence interval; CP: capsule preserved; CR: capsule repaired; CU: capsule unrepaired;*

Supplementary Figure 39: Forest plot of the pain MCID last follow-up. *SD: standard deviation; MCID: minimal clinically important difference; CI: confidence interval; CP: capsule preserved; CR: capsule repaired; CU: capsule unrepaired;*

Supplementary Figure 40: Forest plot of the overall complications rate. *CI: confidence interval; CP: capsule preserved; CR: capsule repaired; CU: capsule unrepaired;*

Supplementary Figure 41: Forest plot of the DVT/PE rate. *DVT: deep vein thrombosis; PE: pulmonary embolism; CI: confidence interval; CP: capsule preserved; CR: capsule repaired; CU: capsule unrepaired;*

Supplementary Figure 42: Forest plot of the nerve injury rate. *CI: confidence interval; CP: capsule preserved; CR: capsule repaired; CU: capsule unrepaired;*

Supplementary Figure 43: Forest plot of the infection rate. *CI: confidence interval; CP: capsule preserved; CR: capsule repaired; CU: capsule unrepaired;*

Supplementary Figure 44: Forest plot of the haematoma rate. *CI: confidence interval; CP: capsule preserved; CR: capsule repaired; CU: capsule unrepaired;*

Supplementary Figure 45: Forest plot of the revision rate. *CI: confidence interval; CP: capsule preserved; CR: capsule repaired; CU: capsule unrepaired;*

Supplementary Figure 46: Forest plot of the THA conversion rate. *THA: total hip arthroplasty; CI: confidence interval; CP: capsule preserved; CR: capsule repaired; CU: capsule unrepaired;*

Supplementary Figure 47: Forest plot of the preoperative mHHS. *SD: standard deviation; mHHS: modified Harris Hip Score; CI: confidence interval; CP: capsule preserved; CR: capsule repaired; CU: capsule unrepaired;*

Supplementary Figure 48: Forest plot of the preoperative iHOT. *SD: standard deviation; iHOT: International Hip Outcome Tool; CI: confidence interval; CP: capsule preserved; CR: capsule repaired; CU: capsule unrepaired;*

Supplementary Figure 49: Forest plot of the preoperative HOS ADL. *SD: standard deviation; HOS‐ADL: Hip Outcome Score – Activities of Daily Living; CI: confidence interval; CP: capsule preserved; CR: capsule repaired; CU: capsule unrepaired;*

Supplementary Figure 50: Forest plot of the preoperative HOS SSS. *SD: standard deviation; HOS‐SSS: Hip Outcome Score ‐ Sports Subscale; CI: confidence interval; CP: capsule preserved; CR: capsule repaired; CU: capsule unrepaired;*

Supplementary Figure 51: Forest plot of the preoperative NAHS. *SD: standard deviation; NAHS: Non‐Arthritic Hip Score; CI: confidence interval; CP: capsule preserved; CR: capsule repaired; CU: capsule unrepaired;*

Supplementary Figure 52: Forest plot of the preoperative VAS. *SD: standard deviation; VAS: visual analogue score; CI: confidence interval; CP: capsule preserved; CR: capsule repaired; CU: capsule unrepaired;*

Supplementary Figure 53: Forest plot of the mHHS 2-12 months postoperatively. *SD: standard deviation; mHHS: modified Harris Hip Score; CI: confidence interval; CP: capsule preserved; CR: capsule repaired; CU: capsule unrepaired;*

Supplementary Figure 54: Forest plot of the mHHS 24 months postoperatively. *SD: standard deviation; mHHS: modified Harris Hip Score; CI: confidence interval; CP: capsule preserved; CR: capsule repaired; CU: capsule unrepaired;*

Supplementary Figure 55: Forest plot of the HOS ADL 3-12 months postoperatively. *SD: standard deviation; HOS‐ADL: Hip Outcome Score – Activities of Daily Living; CI: confidence interval; CP: capsule preserved; CR: capsule repaired; CU: capsule unrepaired;*

Supplementary Figure 56: Forest plot of the HOS ADL 24 months postoperatively. *SD: standard deviation; HOS‐ADL: Hip Outcome Score – Activities of Daily Living; CI: confidence interval; CP: capsule preserved; CR: capsule repaired; CU: capsule unrepaired;*

Supplementary Figure 57: Forest plot of the HOS SSS 3 months postoperatively. *SD: standard deviation; HOS‐SSS: Hip Outcome Score ‐ Sports Subscale; CI: confidence interval; CP: capsule preserved; CR: capsule repaired; CU: capsule unrepaired;*

Supplementary Figure 58: Forest plot of the HOS SSS 6 months postoperatively. *SD: standard deviation; HOS‐SSS: Hip Outcome Score ‐ Sports Subscale; CI: confidence interval; CP: capsule preserved; CR: capsule repaired; CU: capsule unrepaired;*

Supplementary Figure 59: Forest plot of the HOS SSS 12 months postoperatively. *SD: standard deviation; HOS‐SSS: Hip Outcome Score ‐ Sports Subscale; CI: confidence interval; CP: capsule preserved; CR: capsule repaired; CU: capsule unrepaired;*

Supplementary Figure 60: Forest plot of the HOS SSS 24 months postoperatively. *SD: standard deviation; HOS‐SSS: Hip Outcome Score ‐ Sports Subscale; CI: confidence interval; CP: capsule preserved; CR: capsule repaired; CU: capsule unrepaired;*

Supplementary Figure 61: Forest plot of the HOOS ADL 6-24 months postoperatively. *SD: standard deviation; HOOS ADL: Hip disability and Osteoarthritis Outcome Score – Activities of Daily Living; CI: confidence interval; CP: capsule preserved; CR: capsule repaired; CU: capsule unrepaired;*

Supplementary Figure 62: Forest plot of the HOOS Pain 6-24 months postoperatively. *SD: standard deviation; HOOS: Hip disability and Osteoarthritis Outcome Score; CI: confidence interval; CP: capsule preserved; CR: capsule repaired; CU: capsule unrepaired;*

Supplementary Figure 63: Forest plot of the HOOS QoL 6-24 months postoperatively. *SD: standard deviation; HOOS QoL: Hip disability and Osteoarthritis Outcome Score – Quality of Life; CI: confidence interval; CP: capsule preserved; CR: capsule repaired; CU: capsule unrepaired;*

Supplementary Figure 64: Forest plot of the HOOS Sport 3-24 months postoperatively. *SD: standard deviation; HOOS: Hip disability and Osteoarthritis Outcome Score; CI: confidence interval; CP: capsule preserved; CR: capsule repaired; CU: capsule unrepaired;*

Supplementary Figure 65: Forest plot of the HOOS Symptoms 6-24 months postoperatively. *SD: standard deviation; HOOS: Hip disability and Osteoarthritis Outcome Score; CI: confidence interval; CP: capsule preserved; CR: capsule repaired; CU: capsule unrepaired;*

Supplementary Figure 66: Forest plot of the HAGOS ADL 3-24 months postoperatively. *SD: standard deviation; HAGOS ADL: Copenhagen Hip and Groin Outcome Score – Activities of Daily Living; CI: confidence interval; CP: capsule preserved; CR: capsule repaired; CU: capsule unrepaired;*

Supplementary Figure 67: Forest plot of the HAGOS Pain 3-24 months postoperatively. *SD: standard deviation; HAGOS: Copenhagen Hip and Groin Outcome Score; CI: confidence interval; CP: capsule preserved; CR: capsule repaired; CU: capsule unrepaired;*

Supplementary Figure 68: Forest plot of the HAGOS QoL 3-24 months postoperatively. *SD: standard deviation; HAGOS QoL: Copenhagen Hip and Groin Outcome Score Hip disability and Osteoarthritis Outcome Score – Quality of Life; CI: confidence interval; CP: capsule preserved; CR: capsule repaired; CU: capsule unrepaired;*

Supplementary Figure 69: Forest plot of the HAGOS Sport 3-24 months postoperatively. *SD: standard deviation; HAGOS: Copenhagen Hip and Groin Outcome Score; CI: confidence interval; CP: capsule preserved; CR: capsule repaired; CU: capsule unrepaired;*

Supplementary Figure 70: Forest plot of the HAGOS Symptoms 3-24 months postoperatively. *SD: standard deviation; HAGOS: Copenhagen Hip and Groin Outcome Score; CI: confidence interval; CP: capsule preserved; CR: capsule repaired; CU: capsule unrepaired;*

Supplementary Figure 71: Forest plot of the VAS 7 day – 3 months postoperatively. *SD: standard deviation; VAS: visual analogue score; CI: confidence interval; CP: capsule preserved; CR: capsule repaired; CU: capsule unrepaired;*

Supplementary Figure 72: Forest plot of the VAS 6-12 months postoperatively. *SD: standard deviation; VAS: visual analogue score; CI: confidence interval; CP: capsule preserved; CR: capsule repaired; CU: capsule unrepaired;*

Supplementary Figure 73: Forest plot of the VAS 24 months postoperatively. *SD: standard deviation; VAS: visual analogue score; CI: confidence interval; CP: capsule preserved; CR: capsule repaired; CU: capsule unrepaired;*
